# IL-37d suppresses Rheb-mTORC1 axis independently of TCS2 to alleviate alcoholic liver disease

**DOI:** 10.1038/s42003-024-06427-8

**Published:** 2024-06-21

**Authors:** Nuo Chen, Xiaoyu Wang, Yaxin Guo, Ming Zhao, Baihui Cao, Bing Zhan, Yubin Li, Tian Zhou, Faliang Zhu, Chun Guo, Yongyu Shi, Qun Wang, Lining Zhang, Yan Li

**Affiliations:** 1 Department of Immunology, School of Basic Medical Science, Shandong University, Jinan, China; 2 Department of Pathogen Biology, School of Basic Medical Science, Shandong University, Jinan, China

**Keywords:** Alcoholic liver disease, Transcriptomics

## Abstract

Tuberous sclerosis complex 2 (TSC2) crucially suppresses Rheb activity to prevent mTORC1 activation. However, mutations in TSC genes lead to mTORC1 overactivation, thereby causing various developmental disorders and cancer. Therefore, the discovery of novel Rheb inhibitors is vital to prevent mTOR overactivation. Here, we reveals that the anti-inflammatory cytokine IL-37d can bind to lysosomal Rheb and suppress its activity independent of TSC2, thereby preventing mTORC1 activation. The binding of IL-37d to Rheb switch-II subregion destabilizes the Rheb-mTOR and mTOR-S6K interactions, further halting mTORC1 signaling. Unlike TSC2, IL-37d is reduced under ethanol stimulation, which results in mitigating the suppression of lysosomal Rheb-mTORC1 activity. Consequently, the recombinant human IL-37d protein (rh-IL-37d) with a TAT peptide greatly improves alcohol-induced liver disorders by hindering Rheb-mTORC1 axis overactivation in a TSC2- independent manner. Together, IL-37d emerges as a novel Rheb suppressor independent of TSC2 to terminate mTORC1 activation and improve abnormal lipid metabolism in the liver.

## Abstract (web summary)

In this study, IL-37d has been evidenced to bind lysosomal Rheb and suppress its activation in mTORC1 independent of TSC2. In hepatocyte, IL-37d is responsively decreased by ethanol and the recombinant TAT-rh-IL-37d protein improves alcohol-induced liver disorders via Rheb-mTORC1 axis.

## Introduction

Rheb binds directly to and activates the mechanistic (or mammalian) target of rapamycin complex 1 (mTORC1) to promote cell size, growth, and proliferation^1^. Although several lines of evidence have shown that the activated mTORC1 can be found on endosomes, autosomes, Golgi, the endoplasmic reticulum (ER), cytoplasm, and nucleus, the lysosomal surface is broadly accepted as the site where GTP-bound Rheb, rather than that of GDP-binding, targets mTOR for activation in the presence of nutrient or insulin^2,3^. As such, the spatial and subcellular regulation of Rheb is important for triggering mTORC1 activation. For human Rheb (1–184aa) lysosomal translocation, the cysteine residue at 181 within its CAAX motif (181–184aa) of the C-terminal requires farnesylation, which can increase the hydrophobicity to facilitate the membrane binding of the Rheb C-terminus^4,5^. Then the farnesylated Rheb acquires the ability to localize on the lysosome surface and thereby get access to mTOR, which is the core and catalytic component of mTORC1 containing mTOR, Raptor, mLst8, DEPTOR, and PRAS40^6^. The binding of Rheb to mTOR causes a conformational change to expose the mTOR kinase domain to substrate, such as PS6K and 4EBP1, thereby increasing the substrate accessibility of mTORC1^7^. Based on these findings, Rheb farnesylation-mediated lysosome translocation and thereafter the direct binding of mTOR are two important steps for mTORC1 activation.

Similar to all small GTPase, Rheb has two forms of inactive GDP-bound and active GTP-bound states, which enables Rheb as a switch modulator of mTORC1^8^. Rheb regulates mTORC1 activation is largely dependent on its GTPase activity. The TSC complex (tuberous sclerosis complex), including TSC1, TSC2, and TBC1 domain family, member 7 (TBC1D7), can serve as the GTPase activating protein (GAP) towards Rheb and suppress its activity by converting it to the inactive Rheb-GDP-bound form in a TSC2-dependent manner. Structural analysis confirms TSC2-GAP-Rheb complimentary interactions. In the TSC2-GAP-Rheb complex, an asparagine thumb (N1643) of TSC2 stabilizes γ-phosphate of GTP to enhance GTP hydrolysis of Rheb and thus accelerate its activity termination^9^. Reciprocally, TSC2 inhibition of Rheb activity can be relieved by growth factors and insulin via disassociating Rheb from mTORC1 in the lysosome surface, which is induced by AKT-mediated TSC2 phosphorylation in response to insulin^3^. However, mutations of *TSC1* or *TSC2* genes often occur, and their mutations can cause mTORC1 hyperactivation to facilitate tuberous sclerosis, which is an autosomal dominant genetic disease with severe neurological impairments and manifestations, such as epilepsy, intellectual disability, and autism^10–12^. Recently, mTORC1 overactivation has also been evidenced to crucially induce alcoholic liver disease (ALD), which is a leading cause of chronic liver diseases worldwide to induce more than 3 million deaths per year^13,14^. More importantly, TSC1 is decreased in ALD patients and mouse models. The persistent activation of mTORC1 signaling caused by the reduced TSC1 further promotes liver injury in alcoholic hepatitis^15^. Regarding this, Rheb activity needs to be tightly controlled. Furthermore, the critical role of mTORC1 overactivation in multiple diseases, including ALD, requires searching for novel negative regulators of Rheb independent of TSC2.

IL-37 is an important anti-inflammation cytokine in preventing a variety of diseases^16^. It has five different isoforms, IL-37a-e. Among these different splicing products,

IL-37b is the longest isoform and is encoded by exons 1, 2, and 4–6. Compared to IL-37b, IL-37d lacks exon 2 and contains 1 and 4–6 exon transcripts. Many reports have demonstrated that both IL-37b and IL-37d possess biological activities^17,18^. Both of them have the beta-fold barrel structure encoded by exon 4–6, which is required for their biological activity, while the peptides of exons 1 and 2 may be extracellularly cleaved by unknown proteases^19^. The anti-inflammation function of IL-37 depends on either extracellular (receptor-mediated) or intracellular (nuclear function) pathways. Extracellular IL-37 directly binds cell surface IL-18 receptor a (IL-18Ra) and IL-1 receptor 8 (IL-1R8) to transduce anti-inflammatory signals. Intracellular IL-37 can interact with samd3 and translocate to the nucleus in dampening pro-inflammatory genes transcription^16^. Besides, IL-37 also reverses the metabolic cost of inflammation, enhances oxidative respiration, and regulates AMPK-mTORC1pathway^20^. We previously reported that cytoplasmic IL-37 can localize on microtubes to inhibit Rac1 GTPase activity and, thus, tumor metastasis^21^. Other spatial and subcellular distribution of IL-37 requires further investigation. Despite as an inducible anti-inflammation cytokine by various pro-inflammatory stimulus, such as LPS, TNFa, and IL-1β, low levels of IL-37 are constitutively expressed in multiple tissues, including lymph nodes, thymus, bone marrow, brain, intestines, adipose, uterus, testis, heart, kidney, prostate, liver, and breast^21,22^. This characteristic profile of IL-37 abundance strongly indicates its other important physiological function in addition to anti-inflammation action.

Herein, we discover that IL-37d is able to localize on the lysosome membrane and directly bind to Rheb. By analyzing their interaction face, the β-trefoil fold 7, 8, and 9 encoded by exon 5 and 6 of IL-37d is required for Rheb binding and switch II subdomain of Rheb are targeted for IL-37d interaction. Lysosomal IL-37d can suppress Rheb activity independently of TSC2, disrupt Rheb-mTOR interaction, and inhibit interactions of mTOR and its substrate, S6K, thereby terminating mTORC1 activation. Physiological IL-37d is responsively reduced by alcohol, and the reduced IL-37d can abolish the suppression of mTORC1 activation via Rheb. Consistently, the rh-IL37d recombinant protein showed a prominent efficacy in improving abnormal lipid metabolism and liver injury in ALD via downregulating the Rheb-mTORC1 axis independent of TSC2. To conclude, we support that IL-37d acts as a novel negative regulator of Rheb to terminate mTOR activation and thus may be a promising target to treat Rheb-mTORC1 axis overactivation related multiple diseases.

## Results

### IL-37d negatively regulates mTORC1 activity by Rheb

Although prior reports demonstrate that IL-37b can activate AMPK and thus inhibit mTOR activity, whether IL-37d directly regulates mTORC1 remains vague. We previously found that intracellular mature IL-37b can bind directly to and inactivate Rac1 by targeting its CAAX motif of the C-terminus, thereby inhibiting tumor cell migration and tumor metastasis^21^. Considering that Rac1 and Rheb share the same CAAX motif, we hypothesized that IL-37d may suppress mTORC1 activation via Rheb.

**Fig. 1:**
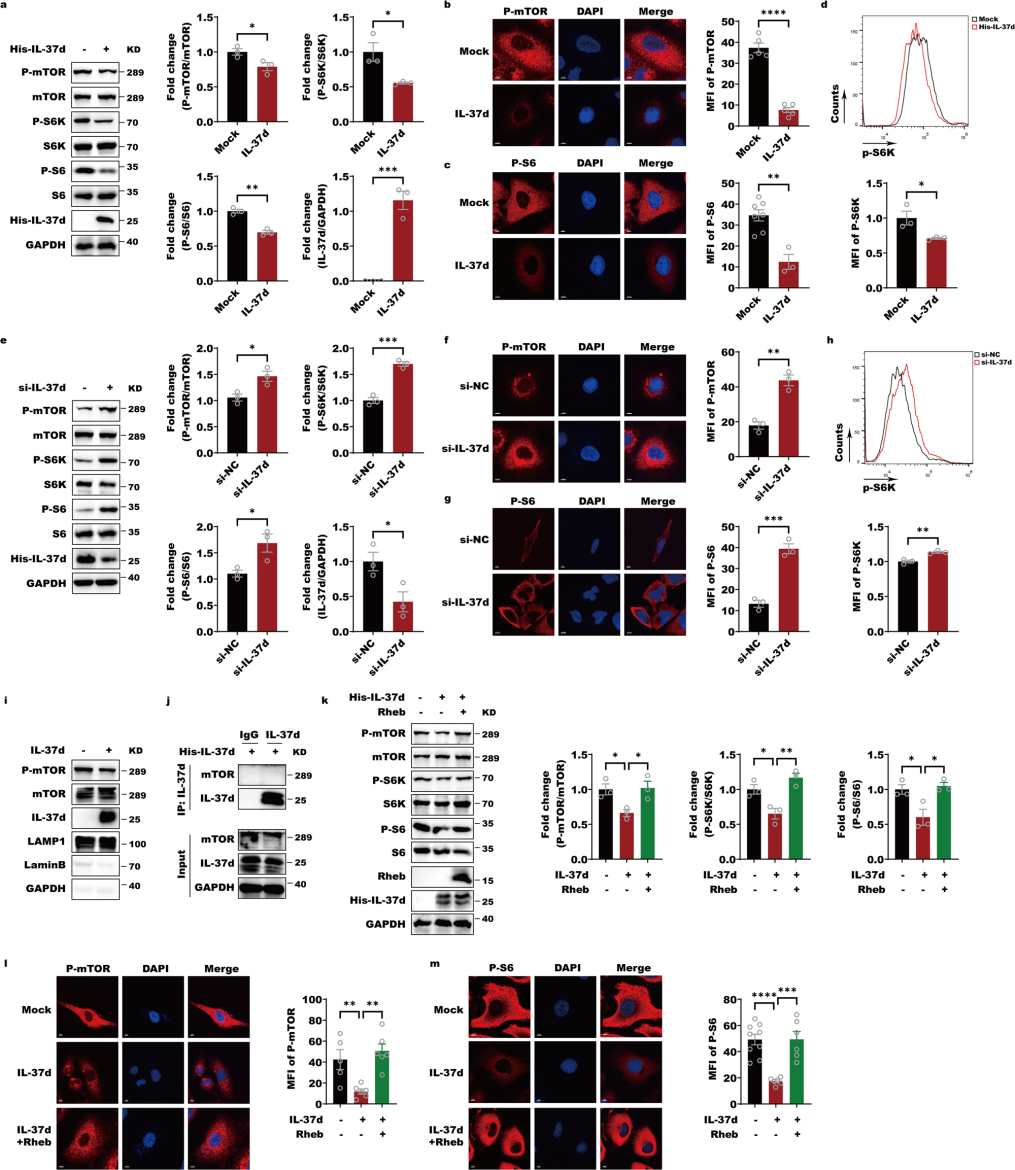
IL-37d negatively regulates mTORC1 activity by Rheb. **a** A549 cells were transfected with IL-37d expressing plasmids and the protein levels of p-S6, p-S6K, and p-mTOR were then evaluated by western blot with indicated antibodies. The quantitative analyses of p-S6, p-S6K, and p-mTOR were performed. Data were shown as mean ± SEM (*n* = 3 biological replicates) and were analyzed using unpaired two-tailed Student’s *t*-test, *P < 0.05, **P < 0.01, ***P < 0.001. **b** A549 cells were transfected with IL-37d expressing plasmids and at 24 h transfection, cells were fixed and immuno-stained with antibody against p-mTOR and DAPI. The quantitative analyse of p-mTOR was performed. Data were shown as mean ± SEM (*n* = 5 biological replicates) and were analyzed using unpaired two-tailed Student’s *t*-test, ****P < 0.0001. **c** A549 cells were transfected with IL-37d expressing plasmids and at 24 h transfection, cells were fixed and immuno-stained with antibody against p-S6 and DAPI. The quantitative analyses of p-S6 were performed. Data were shown as mean ± SEM (*n* = 3–7 biological replicates) and were analyzed using unpaired two-tailed Student’s *t*-test, **P < 0.01. **d** A549 cells were transfected with IL-37d plasmid or control plasmid, respectively. The cells were transfection for 24 h, followed by p-S6K staining for flow cytometry analysis at the excitation wavelength 594. The quantitative analyses of p-S6K were performed. Data were shown as mean ± SEM (*n* = 3 biological replicates) and were analyzed using unpaired two-tailed Student’s *t*-test, *P < 0.05. **e** HepG2 cells were infected by small RNAs against control (si-NC) or IL-37d (si-IL-37d), respectively. And the protein levels of p-S6, p-S6K, and p-mTOR were then evaluated by western blot with indicated antibodies. The quantitative analyses of p-S6, p-S6K, and p-mTOR were performed. Data were shown as mean ± SEM (*n* = 3 biological replicates) and were analyzed using unpaired two-tailed Student’s *t*-test, *P < 0.05, ***P < 0.001. **f** HepG2 cells were infected by small RNAs against control (si-NC) or IL-37d (si-IL-37d), respectively. At 24 h transfection, cells were fixed and immuno-stained with antibody against p-mTOR and DAPI. The quantitative analyses of p-mTOR were performed. Data were shown as mean ± SEM (*n* = 3 biological replicates) and were analyzed using unpaired two-tailed Student’s *t*-test, **P < 0.01. **g** HepG2 cells were infected by small RNAs against control (si-NC) or IL-37d (si-IL-37d), respectively. At 24 h transfection, cells were fixed and immuno-stained with antibody against p-S6 and DAPI. The quantitative analyses of p-S6 were performed. Data were shown as mean ± SEM (*n* = 3 biological replicates) and were analyzed using unpaired two-tailed Student’s *t*-test, ***P < 0.001. **h** HepG2 cells were infected by small RNAs against control (si-NC) or IL-37d (si-IL-37d), respectively. The cells were transfected for 24 h, followed by p-S6K staining for flow cytometry analysis at the excitation wavelength 594. The quantitative analyses of p-S6K were performed. Data were shown as mean ± SEM (*n* = 3 biological replicates) and were analyzed using unpaired two-tailed Student’s *t*-test, **P < 0.01. **i** A549 cells were transfected with control or IL-37d expressing plasmids, respectively. The lysosome compartments were isolated from the resulting cells and subjected to western blot with indicated antibodies. **j** HEK-293T cells were transfected with IL-37d expressing plasmid. Cell lysates of the resulting cells were incubated with IgG or IL-37d antibody. Then, the protein A/G were added and the cell pellets were subjected to western blot with indicated antibodies. **k** A549 cells were transfected with control or IL-37d expressing plasmids alone or together with Rheb expressing plasmids. Cell lysates of the transfection cells were performed by western blot with indicated antibodies. The quantitative analyses of p-S6, p-S6K, and p-mTOR were performed. Data were shown as mean ± SEM (*n* = 3 biological replicates) and were analyzed using one-way ANOVA with Tukey’s multiple comparison test, *P < 0.05, **P < 0.01. **l** A549 cells were transfected with control or IL-37d expressing plasmids alone or together with Rheb expressing plasmids. At 24 h transfection, cells were fixed and immuno-stained with antibody against p-mTOR and DAPI. The quantitative analyses of p-mTOR were performed. Data were shown as mean ± SEM (*n* = 5–6 biological replicates) and were analyzed using one-way ANOVA with Tukey’s multiple comparison test, **P < 0.01. **m** A549 cells were transfected with control or IL-37d expressing plasmids alone or together with Rheb expressing plasmids. At 24 h transfection, cells were fixed and immuno-stained with antibody against p-S6 and DAPI. The quantitative analyses of p-S6 were performed. Data were shown as mean ± SEM (*n* = 6–9 biological replicates) and were analyzed using one-way ANOVA with Tukey’s multiple comparison test, ***P < 0.001, ****P < 0.0001.

We firstly observed that overexpression of IL-37d obviously suppressed mTORC1 phosphorylation and the phosphorylations of mTORC1 downstream substrate, S6K and S6 under basic conditions through the western blot, immunofluorescence stain (IF) and cytometry flow analyses (Fig. 1a–d). Under starvation and serum replenishing conditions, IL-37d overexpression also reduced cell size and growth and mTORC1 activation illustrated by phosphorylations of mTORC1, S6K, and S6 (Fig. S1a, b). Reciprocally, downregulation of IL-37d reversed these effects in HepG2 cells, especially in basic and starvation conditions (Fig. 1e–h and Fig. S1c–e). mTORC1 activation needs mTORC1 lysosomal translocation in the presence of amino acid and Rheb-mediated mTORC1 phosphorylation in the presence of growth factors, like insulin^23–26^. By isolating lysosome subtraction, we observed that IL-37d didn’t impact mTORC1 lysosome translocation. However, the phosphorylation of mTORC1 in the lysosome compartment was remarkedly inhibited in response to IL-37d (Fig. 1i). These data supported that the inhibitory effect of IL-37d on mTORC1 activation was not through influencing mTORC1 lysosomal localization. Furthermore, there was no direct interaction between mTOR-IL-37d (Fig. 1j). To further gain insight into whether IL-37d inhibitory role is dependent on Rheb, we performed experiments to evaluate the mTORC1 activity by simultaneous overexpression of Rheb and IL-37d in A549 cells. The results showed that Rheb overexpression remarkably abolished IL-37d-caused inhibitions on phosphorylations of mTORC1 and its substrates S6K and S6 (Fig. 1k–m). These data suggested that IL-37d-mediated inhibition of mTORC1 is dependent on Rheb.

### IL-37d targets lysosomal Rheb to inhibit its activity

**Fig. 2:**
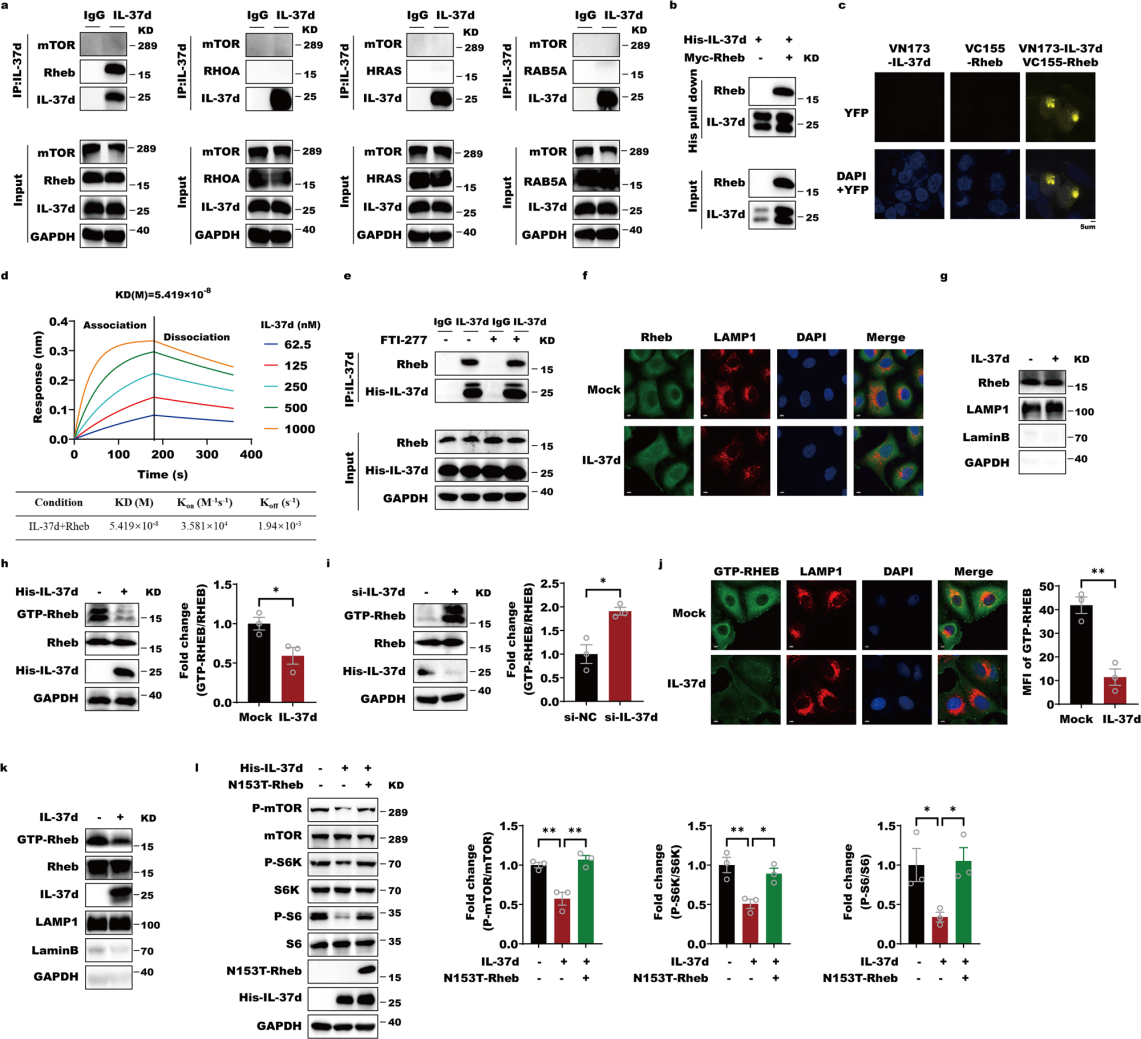
IL-37d targets lysosomal Rheb to inhibit its activity. **a** HEK-293T cells were transfected with Rheb, RHOA, HRAS or RAB5A expressing plasmids, respectively. Cell lysates of the resulting cells were incubated with IgG or IL-37d antibody. Then, the protein A/G were added and the cell pellets were subjected to western blot with indicated antibodies. **b** The purified recombinant protein IL-37d with his tag was incubated with or without myc-Rheb protein at 4 °C for 6 h. The Ni-agarose was then added to pull down proteins that bound to his tag-IL-37d. After high-speed centrifugation, the cell pellets were subjected to western blot with indicated antibodies. **c** Representative BiFC fluorescent images of HEK-293T cells transfected with 2 μg plasmid encoding IL-37d or Rheb fused to the fluorescent protein fragments indicated in each panel. **d** Biolayer interferometry binding analysis of the IL-37d protein to immobilized Rheb protein. The experiments were repeated with different protein preparations and one representative set of curves is shown. Dotted lines correspond to a global fit of the data using a 1:1 binding model. **e** A549 cells were transfected with control or IL-37d expressing plasmids, respectively. The transfected cells were treated with 10 μM an FTase inhibitor (FTI), FTI-277, for 16 h, and the resulting cell lysates were incubated with IgG or IL-37d antibody. Then, the protein A/G were added and the cell pellets were subjected to western blot with indicated antibodies. **f** A549 cells were transfected with IL-37d plasmid or control plasmid, respectively. At 24 h transfection, cells were fixed and immuno-stained with antibodies against Rheb and lysosome membrane protein LAMP1. **g** A549 cells were transfected with IL-37d plasmid or control plasmid, respectively. The lysosome compartments were isolated from the transfected cells and subjected to western blot with indicated antibodies. **h** A549 cells were transfected with control or IL-37d expressing plasmids, respectively. The total cell lysates were subjected to western blot with indicated antibodies. The quantitative analyses of GTP-Rheb were performed. Data were shown as mean ± SEM (*n* = 3 biological replicates) and were analyzed using unpaired two-tailed Student’s *t-*test, *P < 0.05. **i** HepG2 cells were infected by small RNAs against control (si-NC) or IL-37d (si-IL-37d), respectively. The total cell lysates were subjected to western blot with indicated antibodies. The quantitative analyse of GTP-Rheb was performed. Data were shown as mean ± SEM (*n* = 3 biological replicates) and were analyzed using unpaired two-tailed Student’s *t*-test, *P < 0.05. **j** A549 cells were transfected with control or IL-37d expressing plasmids, respectively. At 24 h transfection, cells were fixed and immuno-stained with antibody against GTP-Rheb and lysosome membrane protein LAMP1. Data were shown as mean ± SEM (*n* = 3 biological replicates) and were analyzed using unpaired two-tailed Student’s *t*-test, **P < 0.01. **k** A549 cells were transfected with control or IL-37d expressing plasmids, respectively. The lysosome compartments were isolated from the transfected cells and subjected to western blot with indicated antibodies. **l** A549 cells were transfected with control or IL-37d expressing plasmids alone or together with N153T-Rheb expressing plasmids. Cell lysates of the transfection cells were performed by western blot with indicated antibodies. The quantitative analyses of p-S6, p-S6K, and p-mTOR were performed. Data were shown as mean ± SEM (*n*  = 3 biological replicates) and were analyzed using one-way ANOVA with Tukey’s multiple comparison test, *P  < 0.05, **P < 0.01.

To interrogate how IL-37d regulates the Rheb, we assessed the binding ability of IL-37d to Rheb. By co-immunoprecipitation (co-IP) assay, IL-37d was only immunoprecipitated with Rheb, rather than other Ras-related GTPase family members, such as RHOA, HRAS and RAB5A or mTOR, suggesting that IL-37d may specifically bind to Rheb (Fig. 2a and Fig. S2a, b). In addition, the recombinant IL-37d protein pulled down Rheb protein in the pull-down assay (Fig. 2b). In the bimolecular fluorescence complementation (BiFC) experiments, there are positive YFP signals in the prenuclear by transfecting plasmids which express IL-37d and Rheb fusion proteins with N-terminal and C-terminal fragments of Venus fluorescent protein separately (Fig. 2c). In the Biolayer Interferometry (BLI) assay, IL-37d can directly bind Rheb in a dose-dependent manner with a dissociation constant (KD) value (KD(M) = 5.419 × 10^−8^) (Fig. 2d). Based on these data, the physical interactions of IL-37d and Rheb were confirmed. The farnesylation of Rheb is the prerequisite to localize on lysosomes and is required for activating mTORC1^27^. We thus evaluated IL-37d influence on Rheb farnesylation. The results showed that IL-37d-Rheb interaction was not changed by farnesyltransferase inhibitor FTI-277, suggesting that IL-37d-Rheb interaction is not changed by Rheb farnesylation and lysosome localization (Fig. 2e). To further support this hypothesis, the total levels of Rheb on lysosomal surface were also not influenced by IL-37d via the IF assay and isolating lysosome substrates from the whole cell extracts (Fig. 2f, g). Next, we examined the changes in Rheb activity in response to IL-37d, which is a critical step for mTORC1 activation. We found that the GTP-Rheb levels were obviously reduced by IL-37d treatment, while the downregulation of IL-37d caused obvious elevations in GTP-Rheb (Fig. 2h, i). Importantly, IL-37d remarkably inhibited the GTP-bound Rheb distribution on lysosomes (Fig. 2j, k). To further support these findings, the hyperactive Rheb mutant, N153T, which shows the altered guanine nucleotides ability with higher GTP and lower GDP levels^28^, was able to reverse IL-37d inhibitory effects on mTOR activity (Fig. 2l). These observations support that the physical IL-37d-Rheb interaction occurs on lysosome surface and IL-37d can prevent GTP binding to Rheb, thereby suppressing Rheb activity.

### IL-37d-mediated suppression of Rheb is independent of TSC2

**Fig. 3:**
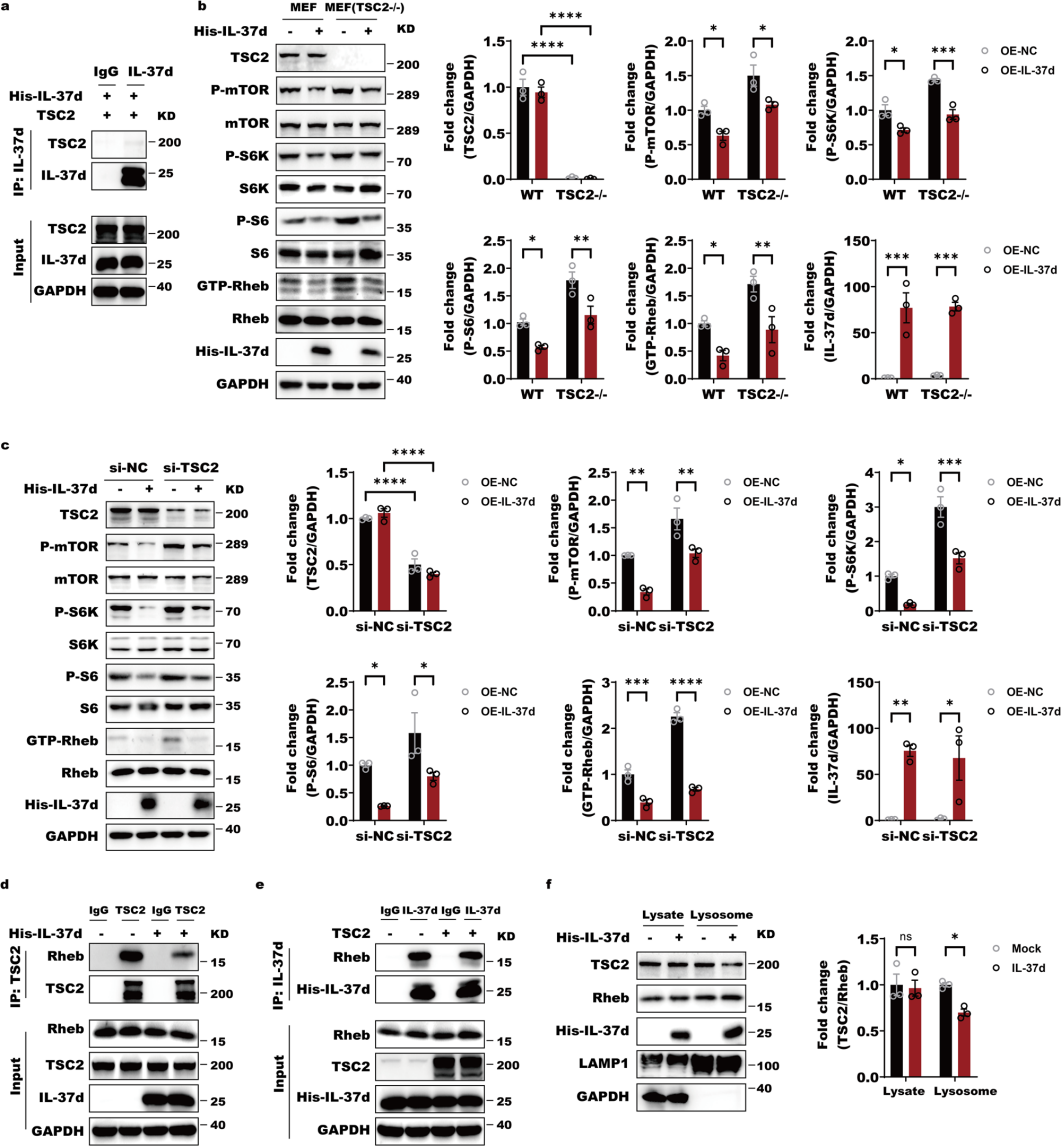
IL-37d-mediated suppression of Rheb is independent of TSC2. **a** HEK-293T cells were co-transfected with IL-37d and TSC2 expressing plasmids. Cell lysates of the resulting cells were incubated with IgG or IL-37d antibody. Then, the protein A/G were added and the cell pellets were subjected to western blot with indicated antibodies. **b** Wild-type TSC2 and TSC2 knockout cells of MEFs (P53−/−) were transfected with or without IL-37 expressing plasmids. And the protein levels of p-S6, p-S6K, and p-mTOR were then evaluated by western blot with indicated antibodies. The quantitative analyses of GTP-Rheb, p-S6, p-S6K, and p-mTOR were performed. Data were shown as mean ± SEM (*n* = 3 biological replicates) and were analyzed using two-way ANOVA with Tukey’s multiple comparison test, *P < 0.05, **P < 0.01, ***P < 0.001, ****P < 0.0001. **c** HepG2 cells were infected with small RNAs against control or TSC2 with or without IL-37d expressing plasmids. And the protein levels of p-S6, p-S6K, and p-mTOR were then evaluated by western blot with indicated antibodies. The quantitative analyses of GTP-Rheb, p-S6, p-S6K, and p-mTOR were performed. Data were shown as mean ± SEM (*n* = 3 biological replicates) and were analyzed using two-way ANOVA with Tukey’s multiple comparison test, *P < 0.05, **P < 0.01, ***P < 0.001, ****P < 0.0001. **d** HEK-293T cells were transfected with IL-37d and TSC2 expressing plasmids alone or together. The whole cell lysates were incubated with IgG or TSC2 antibody. Then, the protein A/G were added and the cell pellets were subjected to western blot with indicated antibodies. **e** HEK-293T cells were transfected with IL-37d and TSC2 expressing plasmids alone or together. The whole cell lysates were incubated with IgG or IL-37d antibody. Then, the protein A/G were added and the cell pellets were subjected to western blot with indicated antibodies. **f** A549 cells were transfected with IL-37d plasmid or control plasmid, respectively. The whole cell lysates and the lysosome compartments were isolated from the transfected cells and subjected to western blot with indicated antibodies. The quantitative analyses of TSC2 were performed. Data were shown as mean ± SEM (*n*  = 3 biological replicates) and were analyzed using two-way ANOVA with Tukey’s multiple comparison test, *P < 0.05.

Abundant evidences demonstrate that TSC2 binds directly to Rheb and inhibits its activity. We then asked whether IL-37d-mediated Rheb suppression is correlated with TSC2. Firstly, we found that IL-37d didn’t interact with TSC2, indicating that IL-37d-mediated the inhibition of Rheb activity via direct binding of Rheb, rather than targeting TSC2 (Fig. 3a). To further exclude TSC2 dependence, IL-37d was still able to remarkably reduce the levels of GTP-bound Rheb and suppress the activation of mTORC1 effector S6K in the *TSC2*-deficient MEF cells (Fig. 3b). Similarly, GTP-bound Rheb and the phosphorylations of S6K and S6 all displayed sharp decreases by IL-37d with TSC2 knockdown (Fig. 3c). These data supported that IL-37d can suppress Rheb activity independently of TSC2. Then we asked how IL-37d and TSC2 cooperate or compete with each other to suppress Rheb activity. Interestingly, the results showed that IL-37d strongly prevented TSC2 binding to Rheb, whereas TSC2 had no effect on IL-37d-Rheb interaction. This observation suggests that IL-37d may possess a higher binding ability of Rheb than TSC2, whereas they may target the same subregions of Rheb to inhibit its activity (Fig. 3d, e). More importantly, we observed that IL-37d greatly prevented TSC2 translocation to the lysosome membrane, suggesting that IL-37d inhibits lysosomal TSC2 interaction with Rheb (Fig. 3f). Collectively, IL-37d functions as a higher affinity suppressor of Rheb independently of TSC2.

### The β-trefoil fold of IL37d is required for binding to switch II motif of Rheb

**Fig. 4:**
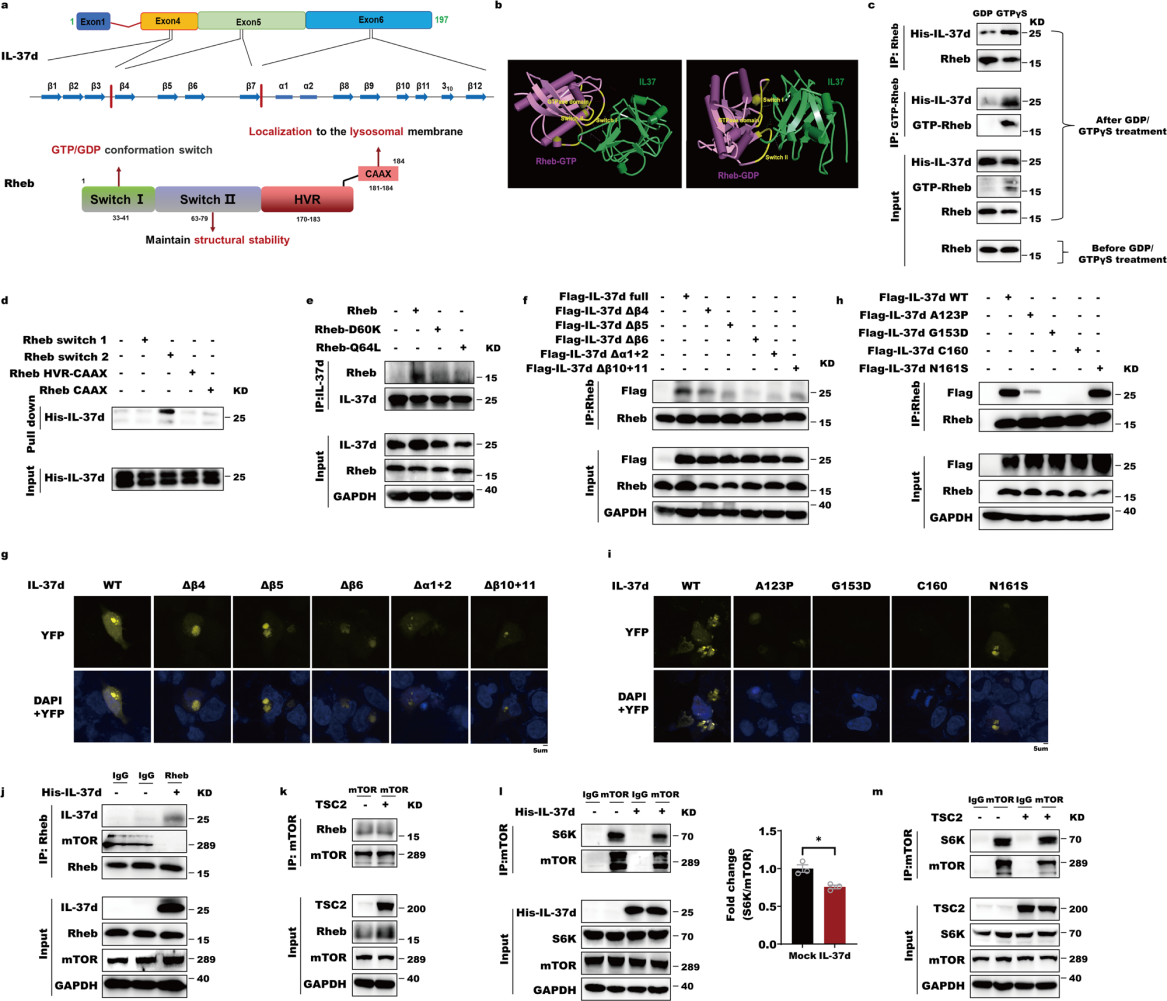
The β-trefoil fold of IL37d is required for binding to switch II motif of Rheb. **a** The schematic diagrams of IL-37d and Rheb. **b** IL-37d-GTP-bound Rheb and IL-37d-GDP bound Rheb protein docking using Z-Dock server. The model represents the best among all the docking poses. Protein-colored green is IL-37d, and protein-colored purple is GTP or GDP-bound Rheb. The GTPase activity domain, switch I and II subregions of Rheb are colored yellow and present in the IL-37d-GTP or GDP bound Rheb interaction faces. **c** 1ug of Rheb or GTP-Rheb protein were incubated with 200 ng of His-IL-37d into 1 ml lysis buffer. Two tubes were incubated with Rheb or GTP-Rheb antibody followed by Ni-agarose beads incubation. And incubate the tubes at 4 °C overnight with gentle agitation. Then the western blot was performed with indicated antibodies. **d** The synthesized Rheb subregions of switch I, switch II, HVR-CAAX, and CAXX was conjugated with biotin. These peptides were incubated with the same amount of purified his-tagged IL-37d at 4 °C for 6 h. The streptavidin agarose was then added, and the cell pellets were subjected to western blot with antibody against IL-37d. **e** HEK-293T cells were infected with or without Rheb expressing plasmids, including wild type or its point mutations D60K- and Q64L. The total cell lysates were incubated with IL-37d antibody followed by protein A/G beads treatment. The cell pellets were used for western bolts with indicated antibodies. **f** HEK-293T cells were transfected with full length of IL-37d or its truncations, including IL-37Δβ4, IL-37Δβ5, IL-37Δβ6, IL-37Δα1 + 2, and IL-37Δβ10 + 11. The whole cell lysates were incubated with Rheb antibody. Then, the protein A/G were added and the cell pellets were subjected to western blot with Flag antibodies. **g** Representative BiFC fluorescent images of HEK-293T cells transfected with 2 μg plasmid encoding IL-37d derivatives, including full length, IL-37Δβ4, IL-37Δβ5, IL-37Δβ6, IL-37Δα1+, and IL-37Δβ10 + 11, and Rheb fused to the fluorescent protein fragments indicated in each panel. **h** HEK-293T cells were transfected with full length of IL-37d or its point mutations, IL-37d-A123P, IL-37d-G153D, IL-37d-C160S, and IL-37d-N161S. The whole cell lysates were incubated with Rheb antibody. Then, the protein A/G were added and the cell pellets were subjected to western blot with Flag antibodies. **i** Representative BiFC fluorescent images of HEK-293T cells transfected with 2 μg plasmid encoding IL-37d derivatives, including full length, IL-37d-A123P, IL-37d-G153D, IL-37d-C160S, and IL-37d-N161S, and Rheb fused to the fluorescent protein fragments indicated in each panel. **j** HEK-293T cells were transfected with or without IL-37d expressing plasmids. The whole cell lysates were incubated with Rheb antibody. Then the protein A/G were added and the cell pellets were subjected to western blot with indicated antibodies. **k** HEK-293T cells were transfected with or without TSC2 expressing plasmids. The whole cell lysates were incubated with mTOR antibody. Then, the protein A/G were added and the cell pellets were subjected to western blot with indicated antibodies. **l** HEK-293T cells were transfected with or without IL-37d expressing plasmids. The whole cell lysates were incubated with mTOR antibody. Then, the protein A/G were added and the cell pellets were subjected to western blot with antibody against S6K. Data were shown as mean ± SEM (*n* = 3 biological replicates) and were analyzed using unpaired two-tailed Student’s *t*-test, *P < 0.05. **m** HEK-293T cells were transfected with or without TSC2 expressing plasmids. The whole cell lysates were incubated with mTOR antibody. Then the protein A/G were added and the cell pellets were subjected to western blot with antibody against S6K.

To further identify the key subregions in IL-37d and Rheb interface, we started to perform a molecular docking analysis of IL-37d and Rheb. We found that switch I (33–41aa) and II (63–79aa) motifs of Rheb were primarily presented to mediate IL-37d binding in both GTP- and GDP-bound status (Fig. 4a, b). Besides, we noted that much more amino acids of GDP-bound Rheb participated in IL-37d binding compared with GTP-bound state, suggesting that GDP-bound Rheb might have higher binding ability than GTP-bound Rheb (Fig. S3a, b and Tables S2, 3). Therefore, we used GTP-γS or GDP to enable Rheb binding GTP or GDP and compared their binding abilities with IL-37d. Interestingly, GTP-Rheb, rather than GDP-Rheb, showed a higher binding ability with IL-37d (Fig. 4c). These data support that GTP-Rheb may be a preferable target for IL-37d, thereby facilitating the suppression of its activity.

As for IL-37d, its β2 (71–74aa) and linker regions of β3 and β4 (84–90) were the major subregions to bind GTP-Rheb, while β7 (128–131aa) and β10–11 (175–198aa) interact with GDP-bound Rheb according to the molecular docking analysis (Fig. 4a, b). Surprisingly, the CAAX motif shared by Rheb and RAC1 in mediating IL-37-Rac1 binding, as we previously reported, was not participated in IL-37d-Rheb interaction. However, the independence of the CAAX motif of IL37d-Rheb binding can provide a reasonable explanation for our prior observations that IL-37d didn’t influence farnesylation-mediated Rheb membrane localization. To further confirm IL-37d and Rheb subregions or key amino acids to mediate their binding, we conducted a series of Rheb and IL-37d truncated deletions to estimate their interactions by co-IP, protein pull-down, and BiFC assays. As we predicted before, the switch II region of Rheb directly binded IL-37d and the mutations in the switch II motif, such as Q64L and D60K, lost the ability to bind IL-37d (Fig. 4b, d, e). Considering that IL37d-mediated suppression of Rheb-N153T activity with altered guanine nucleotides ability, we speculated that the lost interactions of Q64L- and D60K-Rheb with IL-37d might be attributable to their self residues in switch II subdomain, rather than GTP-bound abilities. As for IL-37d, its characteristic β-trefoil fold (β1-12) subregion was required for Rheb binding (Fig. 4f, g). Importantly, we observed that IL-37 mutations within β-trefoil fold encoded by exons 4, 5, and 6, such as A123P, G153D, C160 but not N161S, which are initially discovered in gout patients, lost the ability to bind Rheb, implying that abnormal IL-37d-Rheb interaction is closely related with gout development and may facilitate gout pathogenesis^29^ (Fig. 4h, i).

Rheb regulates mTORC1 activation is largely dependent on its GTPase activity. However, Rheb itself, especially the switch II domain, is required for direct binding to mTOR and thus mTORC1 activation, independently of its GTPase activity^8,30^. Therefore, we further evaluated IL-37d role in controlling Rheb-mTOR interaction. Contrary to expectation, distinct from TSC2, IL-37d inhibited both the interactions of Rheb-mTOR and mTOR-S6K, suggesting that the binding of IL-37d to Switch II of Rheb leads to mTOR disassociations from Rheb and its substrate, thereby facilitating mTOR signal termination (Fig. 4j–m). These data supported that IL-37d was quite different from TSC2 to possess a novel biological activity in terminating mTOR activation. Altogether, the β-trefoil fold of IL37d is required for targeting the switch II motif of Rheb.

### Alcohol downregulates IL-37d to activate mTOR via relieving suppression of Rheb

**Fig. 5:**
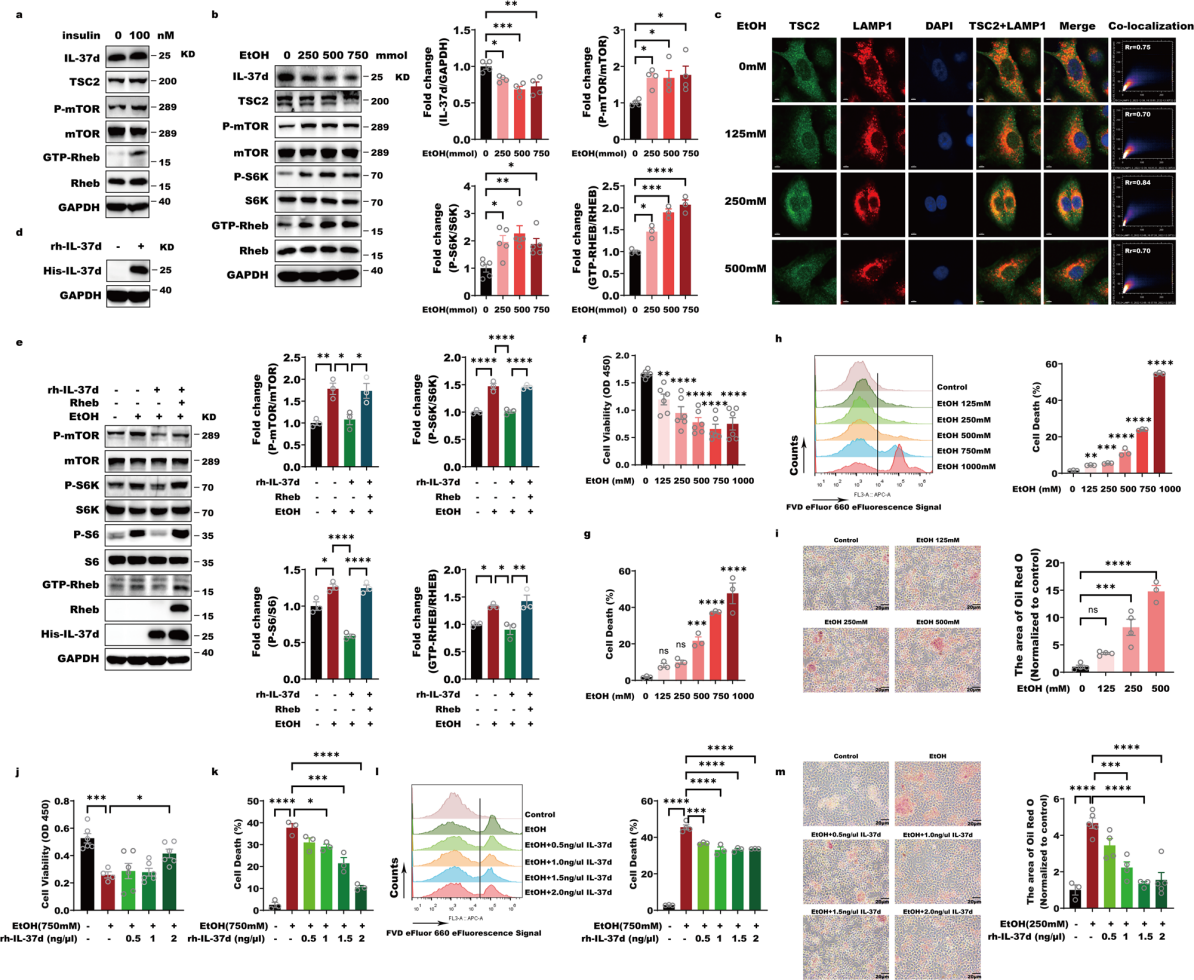
Alcohol downregulates IL-37d to activate mTOR via relieving suppression of Rheb. **a** HepG2 cells were treated with or without 100 nM insulin for 10 min. The whole cell lysates were used for western bolt with indicated antibodies. **b** HepG2 cells were treated with or without increasing alcohol from 250 to 750 mmol for 24 h. The whole cell lysates were used for western bolt with indicated antibodies. The quantitative analyses of GTP-Rheb, p-S6K, and p-mTOR were performed. Data were shown as mean ± SEM (*n* = 3–5 biological replicates) and were analyzed using one-way ANOVA with Tukey’s multiple comparison test, *P < 0.05, **P < 0.01, ***P < 0.001, ****P < 0.0001. **c** HepG2 cells were treated with or without increasing alcohol from 125 to 500 mmol. At 24 h treatment, cells were fixed and immuno-stained with antibodies against TSC2 and lysosome membrane protein LAMP1. **d** The recombinant protein IL-37d with a cell-penetrating polypeptides, TAT, and his tag was purified from bacteria using a Ni-agarose column and confirmed by western blot with IL-37d antibodies. **e** HepG2 cells were stimulated with or without 500 mmol alcohol for 24 h in the presence or absence of Rh-IL37d alone or together with Rheb-expressing plasmids. The total cell lysates were prepared for western blot with indicated antibodies. The quantitative analyses of GTP-Rheb, p-S6, p-S6K, and p-mTOR were performed. Data were shown as mean ± SEM (*n* = 3 biological replicates) and were analyzed using one-way ANOVA with Tukey’s multiple comparison test, *P < 0.05, **P < 0.01, ****P < 0.0001. **f** HepG2 cells were treated with or without increasing alcohol from 125 to 1000 mmol. At 24 h treatment, the absorbance was detected by the absorbance reader on OD450. Data were shown as mean ± SEM (*n* = 6 biological replicates) and were analyzed using one-way ANOVA with Tukey’s multiple comparison test, **P < 0.01, ****P < 0.0001. **g** HepG2 cells were treated with or without increasing alcohol from 125 to 1000 mmol. At 24 h treatment, the number of dead cells was recorded by Trypan blue staining. Data were shown as mean ± SEM (*n* = 3 biological replicates) and were analyzed using one-way ANOVA with Tukey’s multiple comparison test, ***P < 0.001, ****P < 0.0001. **h** HepG2 cells were treated with or without increasing alcohol from 125 to 1000 mmol. At 24 h treatment, dead cells were stained with eBioscience™ Fixable Viability Dye eFluor™ 660 and the number of dead cells was determined by flow cytometry. Data were shown as mean ± SEM (*n* = 3 biological replicates) and were analyzed using one-way ANOVA with Tukey’s multiple comparison test, **P < 0.01, ***P < 0.001, ****P < 0.0001. **i** HepG2 cells were treated with or without increasing alcohol from 125 to 1000 mmol. At 48 h treatment, the treated cells were stained with oil red O and the cells were visualized under an inverted microscope. Data were shown as mean ± SEM (*n* = 3–4 biological replicates) and were analyzed using one-way ANOVA with Tukey’s multiple comparison test, ***P < 0.001, ****P < 0.0001. **j** HepG2 cells were treated with or without increasing alcohol from 125 to 1000 mmol. In the treatment group, different concentrations of rh-IL37d were added 4 h in advance. After ethanol stimulation for 24 h, the absorbance was detected by the absorbance reader on OD450. Data were shown as mean ± SEM (*n* = 4–6 biological replicates) and were analyzed using two-way ANOVA with Tukey’s multiple comparison test, *P < 0.05, ***P < 0.001. **k** HepG2 cells were treated with or without increasing alcohol from 125 to 1000 mmol. In the treatment group, different concentrations of rh-IL37d were added 4 h in advance. After ethanol stimulation for 24 h, the number of dead cells was recorded by Trypan blue staining. Data were shown as mean ± SEM (*n* = 3 biological replicates) and were analyzed using two-way ANOVA with Tukey’s multiple comparison test, *P < 0.05, ***P < 0.001, ****P < 0.0001. **l** HepG2 cells were treated with or without increasing alcohol from 125 to 1000 mmol. In the treatment group, different concentrations of rh-IL37d were added 4 h in advance. After ethanol stimulation for 24 h, dead cells were stained with eBioscience™ Fixable Viability Dye eFluor™ 660 and the number of dead cells was determined by flow cytometry. Data were shown as mean ± SEM (*n* = 3 biological replicates) and were analyzed using two-way ANOVA with Tukey’s multiple comparison test, ***P < 0.001, ****P < 0.0001. **m** HepG2 cells were treated with or without increasing alcohol from 125 to 1000 mmol. In the treatment group, different concentrations of rh-IL37d were added 4 h in advance. After ethanol stimulation for 24 h, the treated cells were stained with oil red O and the cells were visualized under an inverted microscope. Data were shown as mean ± SEM (*n* = 3–5 biological replicates) and were analyzed using two-way ANOVA with Tukey’s multiple comparison test, ***P < 0.001, ****P < 0.0001.

Extensive evidence supports that TSC2 can be phosphorylated by the AKT pathway to disassociate from lysosomal Rheb in the presence of insulin. Herein, we proved that IL-37d suppressed lysosomal Rheb activity, Rheb-mTOR interaction, and mTOR substrate recruitment, thereby terminating mTORC1 activation. To gain insight into the upstream signal of IL-37d, we performed experiments to evaluate IL-37 changes under diverse stimulus, such as growth factors like insulin and alcohol. We observed that both the expressions of IL-37d and TSC2 were not responsible for insulin stimulation, whereas the levels of GTP-Rheb were augmented, implying that insulin is not the upstream signal of IL-37d (Fig. 5a). Surprisingly, we discovered that the global IL-37d level, rather than TSC2, was obviously reduced by ethanol in a dose-dependent manner without influencing the global level of Rheb in HepG2 cells (Fig. 5b). Importantly, alcohol led to reduced levels of IL-37d but caused higher levels of GTP-Rheb and mTOR activation, suggesting that IL-37d is physiologically involved in alcohol metabolism and its reduction may be responsible to mTOR activation via elevating GTP-Rheb (Fig. 5b). Of note, not only total TSC2, but also its lysosomal localizations, were not influenced by alcohol (Fig. 5c). These findings indicate that mTORC1 overactivation caused by elevations of GTP-Rheb with alcohol is independent on TSC2. From the above data, the responsive reduction of IL-37d, rather than TSC2, contributes to mTOR overactivation by relieving the suppression of Rheb activity in response to alcohol. Although IL-37d functions like IL-37b to have anti-inflammatory roles via either extracellular (receptor-mediated) or intracellular (nuclear function) pathways, we found that Rheb-IL-37d interaction mainly occurred within the cell. Therefore, we designed an IL-37d recombinant protein with a cell-penetrating TAT polypeptide to stress its intracellular role in suppressing Rheb-mTORC1 axis and evaluated its therapeutic effects on alcohol-induced mTOR overactivation. Firstly, we sought to determine that whether human IL-37d recombinant protein with TAT (rh-IL37d) could enter the cells, considering that cellular IL-37d is required for suppression of the Rheb-mTORC1 axis. Firstly, we purified the rh-IL37d recombinant and found that this protein successfully entered the HepG2 cells after 1 h incubation at a concentration of 500 ng/ml. More importantly, the intracellular rh-IL37d was persistently present after 72 h incubation, suggesting that the cellualr rh-IL37d has a high stability with a long-lasting action (Fig. 5d and Fig. S4a–c). Next, we observed that rh-IL37d was able to inhibit the enhanced phosphorylations of mTOR and S6K caused by alcohol treatment (Fig. 5e). Importantly, Rheb overexpression reversed the rh-IL37d-mediated suppressive effect on alcohol-caused mTOR overactivation, suggesting that the inhibitory effect of rh-IL37d on alcohol-induced mTORC1 activation is dependent on Rheb (Fig. 5e). mTORC1 has been reported to facilitate lipid synthesis and storage and thus is closely related with abnormal lipid metabolism in liver^31–33^. Thus, targeting mTORC1 acts as a promising therapeutic approach to improve abnormal lipid metabolism in various liver diseases, such as non-alcoholic fatty liver disease (NAFLD) and alcoholic liver disease (ALD). Thus, we examined IL-37d function in improving abnormal lipid metabolism through negatively regulating mTORC1 activation. In line with previous findings, the alcohol could dose-dependently induce cell death and abnormal lipid accumulation in HepG2 and Huh7 cells from 125 mM to 1 M (Fig. 5f–i and Fig. S4d–f). The rh-IL37d remarkedly reduced alcohol-caused cell death and ameliorated the aberrant lipid storage (Fig. 5j–m and Fig. S4g–i). Altogether, these data indicate that physiological IL-37d reduction, rather than TSC2, is responsible for alcohol-induced mTORC1 overactivation via Rheb. More importantly, the recombinant protein rh-IL37d can prevent alcohol-caused hepatic abnormal lipid accumulation and cell death.

### IL-37d suppresses alcohol-induced mTORC1 overactivation and hepatic steatosis in chronic-binge ethanol-fed mice

**Fig. 6:**
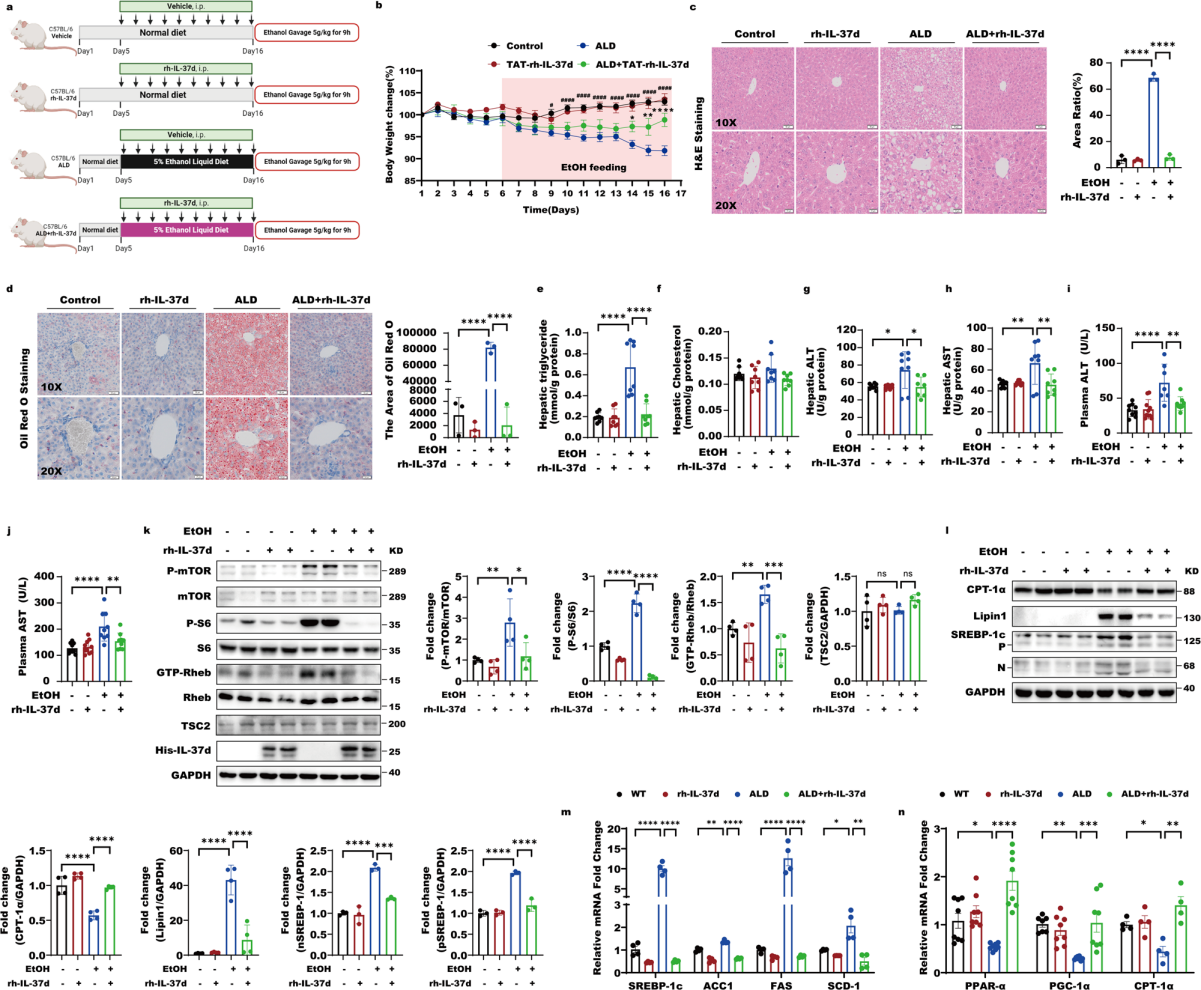
IL-37d suppresses alcohol-induced mTORC1 overactivation and hepatic steatosis in chronic-binge ethanol-fed mice. **a** The timeline of the animal experiment that the rh-IL37d protein attenuates hepatic steatosis in ethanol-fed mice (The mouse images of the flowcharts were credited by Biorender.com). In detail, mice were provided with chronic ethanol feeding (10-d ad libitum oral feeding with the Lieber-DeCarli ethanol liquid diet) plus a single binge ethanol feeding. The paired-fed control mice were given a calorie-matched maltodextrin diet. Control mice and ALD mice per group (*n* = 8–10) accepted vehicle treatment i.p. daily or 1 μg rh-IL37d recombinant protein during the whole experimental period. Then mice received a single gavage of 5 g/kg body weight (31.5% vol/vol) or 45% maltodextrin for control mice followed by 5% alcohol diet on day 16. **b** The changes of body weight in groups of mice (*n* = 8–10) with or without chronic alcohol feeding (WT group or ALD group) in the presence or absence of rh-IL37d treatment. (^**#**^represents the Statistics between the WT group and the ALD group; *represents the Statistics between ALD group and ALD+rh-IL-37d group). Data were shown as mean ± SEM (*n* = 8–10/group) and were analyzed using two-way ANOVA with Tukey’s multiple comparison test, */^**#**^*P* < 0.05, **P < 0.01, ****/^**####**^*P* < 0.0001. **c** The graphs of H&E staining of liver sections in WT, rh-IL37d, ALD and ALD with rh-IL37d groups (*n* = 3) and the area ratio was statistically performed. Data were shown as mean ± SEM (*n* = 3/group) and were analyzed using two-way ANOVA with Tukey’s multiple comparison test, ****P < 0.0001. **d** The graphs of Oil Red O staining of the liver in WT, rh-IL37d, ALD, and ALD with rh-IL37d groups (*n* = 3) and the area of Oil Red O was statistically performed. Data were shown as mean ± SEM (*n* = 3/group) and were analyzed using two-way ANOVA with Tukey’s multiple comparison test, ****P < 0.0001. **e**–**h** The liver function including activities of TG (**e**), total cholesterol (T-CHO) (**f**), ALT (**g**), and AST (**h**) were determined in mice of WT, rh-IL37d, ALD, and ALD with rh-IL37d groups. Data were shown as mean ± SEM (*n* = 6–8/group) and were analyzed using two-way ANOVA with Tukey’s multiple comparison test, *P < 0.05, **P < 0.01, ****P < 0.0001. **i**, **j** The levels of ALT (**i**) and AST (**j**) were analysed from mice of WT, rh-IL37d, ALD, and ALD with rh-IL37d groups. Data were shown as mean ± SEM (*n* = 7–8/group) and were analyzed using two-way ANOVA with Tukey’s multiple comparison test, **P  < 0.01, ****P < 0.0001. **k** The liver lysates of mice from WT, rh-IL37d, ALD, and ALD with rh-IL37d groups (*n* = 4) were subjected to western blot with indicated antibodies. And the quantitative analyses of GTP-Rheb, p-S6, p-mTOR, and TSC2 were performed. Data were shown as mean ± SEM (*n* = 4/group) and were analyzed using two-way ANOVA with Tukey’s multiple comparison test, *P < 0.05, **P < 0.01, ***P < 0.001, ****P < 0.0001. **l** The liver lysates of mice from WT, rh-IL37d, ALD, and ALD with rh-IL37d groups (*n* = 4) were subjected to western blot with indicated antibodies. And the quantitative analyses of nSREBP-1, pSREBP-1, Lipin1, and CPT-1A were performed. Data were shown as mean ± SEM (*n* = 4/group) and were analyzed using two-way ANOVA with Tukey’s multiple comparison test, ***P < 0.001, ****P < 0.0001. **m** The nuclear translocation of SREBP-1, as well as expression of SREBP-1c and its targets, including ACC1, FAS, and SCD1, were reduced by rh-IL37d in chronic-binge ethanol-fed mice. Data were presented as mean ± SEM, *n* = 4–8. *P < 0.05 versus pair-fed mice. Data were shown as mean ± SEM (*n* = 4/group) and were analyzed using one-way ANOVA with Tukey’s multiple comparison test, *P < 0.05, **P < 0.01, ****P < 0.0001. **n** The expressions of key genes involving fatty acid oxidation such as PPARa, CPT-1a, and PGC-1a were analysed. Data were presented as mean ± SEM, *n* = 4–8. *P < 0.05 versus pair-fed mice. Data were shown as mean ± SEM (*n* = 4–8/group) and were analyzed using one-way ANOVA with Tukey’s multiple comparison test, *P < 0.05, **P < 0.01, ***P < 0.001, ****P < 0.0001.

We found that physiological IL-37d is responsively reduced by alcohol, thereby leading to mTORC1 activation and alcohol-induced cell death. Thus, we further examined whether IL-37d has a protective role in ALD via negatively modulating mTORC1 using a chronic-binge ethanol-feeding mouse model (EtOH). Firstly, we evaluated whether intraperitoneal injections of 1 μg rh-IL37d every day had toxic effects on glucose metabolism and peripheral organs function in mice. The results demonstrated that the weights and morphology of the heart, liver, spleen, lung, and kidney were not influenced by rh-IL37d (Fig. S5a–i). The levels of plasma alanine aminotransferase (ALT), aspartate aminotransferase (AST), aspartate aminotransferase/alanine aminotransferase (S/L), alkaline phosphatase (ALP), total protein (TP), albumin (ALB), globulin (GloB), and albumin/globulin (A/G) were neither altered by rh-IL37d. These observations suggest that IL-37d has no toxic effects on liver function in normal mice. The kidney function-related urea, Crea (Cr), and uric acid (UA) levels were neither influenced by rh-IL37d. The levels of blood glucose (GLU) and lactate dehydrogenase (LDH) remained unchanged with rh-IL37d treatment, suggesting that the IL-37d doesn’t impact glucose metabolism in normal mice (Fig. S5j–v). Taken together, the rh-IL37d doesn’t have toxic effects on normal mice. Next, we evaluated the rh-IL37d function in ALD mice (Fig. 6a). We observed that the reductions of mice body weight in response to alcohol were remarkedly prevented by rh-IL37d treatment (Fig. 6b). Hematoxylin and eosin (H&E) staining showed that chronic-binge ethanol administration induced steatosis with small and large lipid droplet vacuoles in the liver compared to that of wild type mice without ethanol treatment (control mice) (Fig. 6c). On the contrary, the rh-IL37d treatments greatly prevented the alcohol-induced liver steatosis in ALD mice (Fig. 6c). Further Oil Red O staining of liver tissue revealed that rh-IL37d obviously reduced ethanol-induced hepatic lipid deposits in ALD mice (Fig. 6d). Correspondingly, the elevations of liver triglyceride levels in ALD mice were remarkedly blocked by rh-IL37d, whereas the cholesterol levels in liver of ALD mice were not changed by alcohol or rh-IL37d (Fig. 6e, f). Importantly, the increases of alanine aminotransferase (ALT) and aspartate transaminase (AST) levels in both liver and plasma caused by chronic-binge ethanol administration were also suppressed by rh-IL37d (Fig. 6g–j).

In line with the observation that the active GTP-bound Rheb was greatly induced by alcohol in HepG2 cells, the GTP-Rheb levels were remarked increased in the liver of ALD mice compared with those of control mice (Fig. 6k). However, the aberrant activation of Rheb-mTORC1 signaling including GTP-Rheb, p-mTOR, p-S6 in ALD mice was dramatically inhibited by rh-IL37d (Fig. 6k). The mTORC1 overactivation can cause the lipid metabolic abnormalities in alcohol-induced hepatic steatosis including lipid synthesis and fatty acid oxidation. Therefore, both the critical transcription factor of the synthesis of fatty acid and triglycerides, SREBP-1, including its precursor (about 125 kDa) and nuclear active forms (about 68 kDa), and the key metabolic enzyme for triglyceride synthetic pathway, lipin 1, display the higher up-regulation. In contrast, the rate-limiting enzyme for fatty acid oxidization, CPT-1α, is remarkedly downregulated by alcohol-induced mTORC1 overactivation. We thus examined the lipid synthesis and fatty acid oxidation in the liver of ALD mice with or without rh-IL37d treatment. In line with previous report^33^, alcohol obviously increased the expressions of lipogenic enzymes including SREBP-1 precursor and nuclear active forms, acetyl-coenzyme A carboxylase (ACC1), fatty acid synthase (FAS) and stearoyl coenzyme A desaturase (SCD1), and lipin 1 but decreased the expressions of lipid utilization and fatty acid oxidation genes including CPT-1α, PPAR-α and PGC-1α (Fig. 6l–n). However, the rh-IL37d treatment greatly prevented their changes in ALD mice, suggesting that IL-37 suppresses and ameliorates alcohol-induced lipid metabolic abnormalities (Fig. 6k–n). Collectively, these results support that the rh-IL37d can inhibit alcohol-induced mTORC1 overactivation and has a protective role against hepatic steatosis in ALD.

### The protective effect of IL-37d in ALD mice relies on Rheb in a TSC2-independent manner

**Fig. 7:**
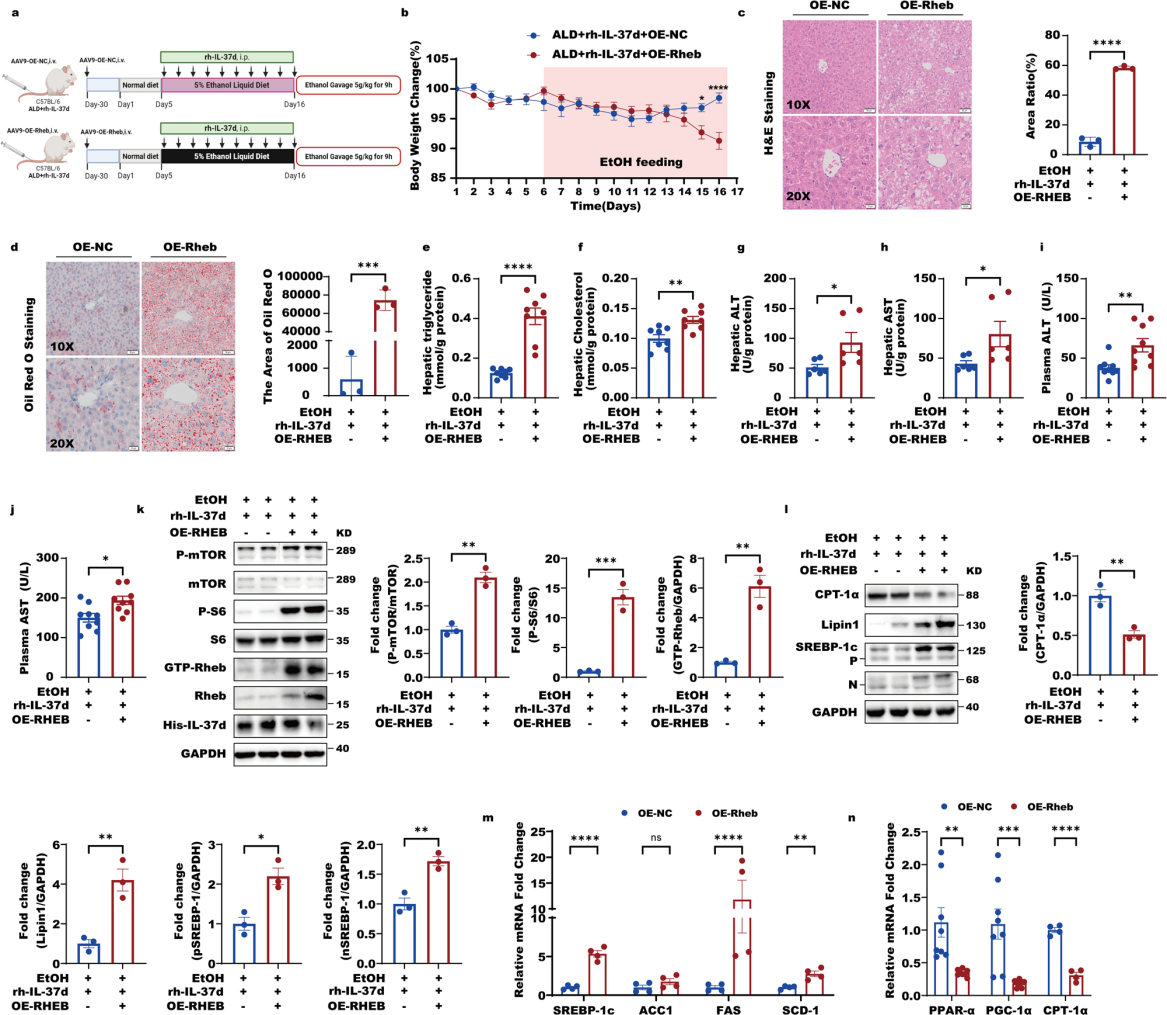
The protective effect of IL-37d in ALD relies on Rheb in a TSC2-independent manner in mice. **a** The timeline of the animal experiment showed that overexpression of Rheb eliminated the therapeutic effect of IL-37d in ethanol-fed mice (The mouse images of the flowcharts were credited by Biorender.com). In detail, mice were given AAV9 virus-containing OE-Rheb or control through tail vein injection one month before ethanol feeding. **b** The changes of body weight in groups of mice (*n* = 8–10) with or without Rheb overexpression (OE-NC group or OE-Rheb group). Data were shown as mean ± SEM (*n* = 8–10/group) and were analyzed using two-way ANOVA with Tukey’s multiple comparison test, *P < 0.05, ****P < 0.0001. **c** The graphs of H&E staining of liver sections in OE-NC and OE-Rheb groups (*n* = 3) and the area ratio was statistically performed. Data were shown as mean ± SEM (*n*  = 3/group) and were analyzed using unpaired two-tailed Student’s *t*-test, ****P < 0.0001. **d** The graphs of Oil Red O staining of the liver in OE-NC and OE-Rheb groups (*n* = 3) and the area of Oil Red O was statistically performed. Data were shown as mean ± SEM (*n*  = 3/group) and were analyzed using unpaired two-tailed Student’s *t-*test, ***P < 0.001. **e**–**h** The liver function including activities of TG (**e**), total cholesterol (T-CHO) (**f**), ALT (**g**), and AST (**h**) were determined in mice of OE-NC and OE-Rheb groups. Data were shown as mean ± SEM (*n* = 6–8/group) and were analyzed using unpaired two-tailed Student’s *t*-test, *P < 0.05, **P < 0.01, ****P < 0.0001. **i**, **j** The levels of ALT (**i**) and AST (**j**) were analysed from mice of OE-NC and OE-Rheb groups (*n* = 9). Data were shown as mean ± SEM (*n* = 8–9/group) and were analyzed using unpaired two-tailed Student’s *t-*test, *P < 0.05, **P < 0.01. **k** The liver lysates of mice from OE-NC and OE-Rheb groups were subjected to western blot with indicated antibodies. And the quantitative analyses of GTP-Rheb, p-S6, p-mTOR, and TSC2 were performed. Data were shown as mean ± SEM (*n* = 3/group) and were analyzed using unpaired two-tailed Student’s *t*-test, **P < 0.01, ***P < 0.001. **l** The liver lysates of mice from OE-NC and OE-Rheb groups were subjected to western blot with indicated antibodies. And the quantitative analyses of nSREBP-1, pSREBP-1, Lipin1, and CPT-1A were performed. Data were shown as mean ± SEM (*n*  = 3/group) and were analyzed using unpaired two-tailed Student’s *t*-test, *P < 0.05, **P < 0.01. **m** The nuclear translocation of SREBP-1, as well as expression of SREBP-1c and its targets, including ACC1, FAS, and SCD1, were induced by overexpression Rheb in chronic-binge ethanol-fed with rh-IL37d mice. Data were shown as mean ± SEM (*n* = 4/group) and were analyzed using one-way ANOVA with Tukey’s multiple comparison test, **P < 0.01, ****P < 0.0001. **n** The expressions of key genes involving fatty acid oxidation, such as PPARa, CPT-1a, and PGC-1a were analysed. Data were presented as mean ± SEM, *n* = 4–8. *P < 0.05 versus OE-NC mice. Data were shown as mean ± SEM (*n*  = 4–8/group) and were analyzed using one-way ANOVA with Tukey’s multiple comparison test, **P < 0.01, ***P < 0.001, ****P < 0.0001.

As we described above, the rh-IL37d can suppress alcohol-induced mTORC1 activation to improve ALD-related lipid metabolic abnormalities and hepatic steatosis. We then further examined whether the protective role of IL-37d in ALD mice was dependent on Rheb but independent of TSC2. To this end, we performed adeno-associated virus 9 (AAV9)-mediated hepatic Rheb overexpression or TSC2 knockdown, which were driven by the thyroxine-binding globulin (TBG) promoter through tail vein injection in the rh-IL37d treated ALD mice. Interestingly, we found that hepatic Rheb overexpression obviously abolished IL-37d improvement in ALD mice, including the inhibition of body weight reductions, abnormal lipid accumulation in the liver illustrated by H&E and Oil Red O staining and measurements of liver triglyceride and cholesterol levels, and the liver and plasma enzymatic activities of ALT and AST (Fig. 7a–j and Fig. S6a–d). The rh-IL37d-mediated suppression of Rheb-mTORC1 signaling, including GTP-Rheb, p-mTOR, and p-S6 in ALD mice was also abrogated (Fig. 7k). Consequently, the reduced expressions of the key metabolic enzymes for the synthesis of fatty acid and triglycerides by rh-IL37d including SREBP-1, ACC1, FAS, SCD1, and lipin 1 were reversed as well by Rheb overexpression in the rh-IL37d treated ALD mice (Fig. 7l–m). Similarly, the elevations of expressions of lipid utilization and fatty acid oxidation genes, including CPT-1α, PPAR-α, and PGC-1α by rh-IL37d were also abrogated by hepatic Rheb overexpression (Fig. 7l, n). These data support that the improvement of IL-37d in alcohol-induced hepatic steatosis is dependent on Rheb.

**Fig. 8:**
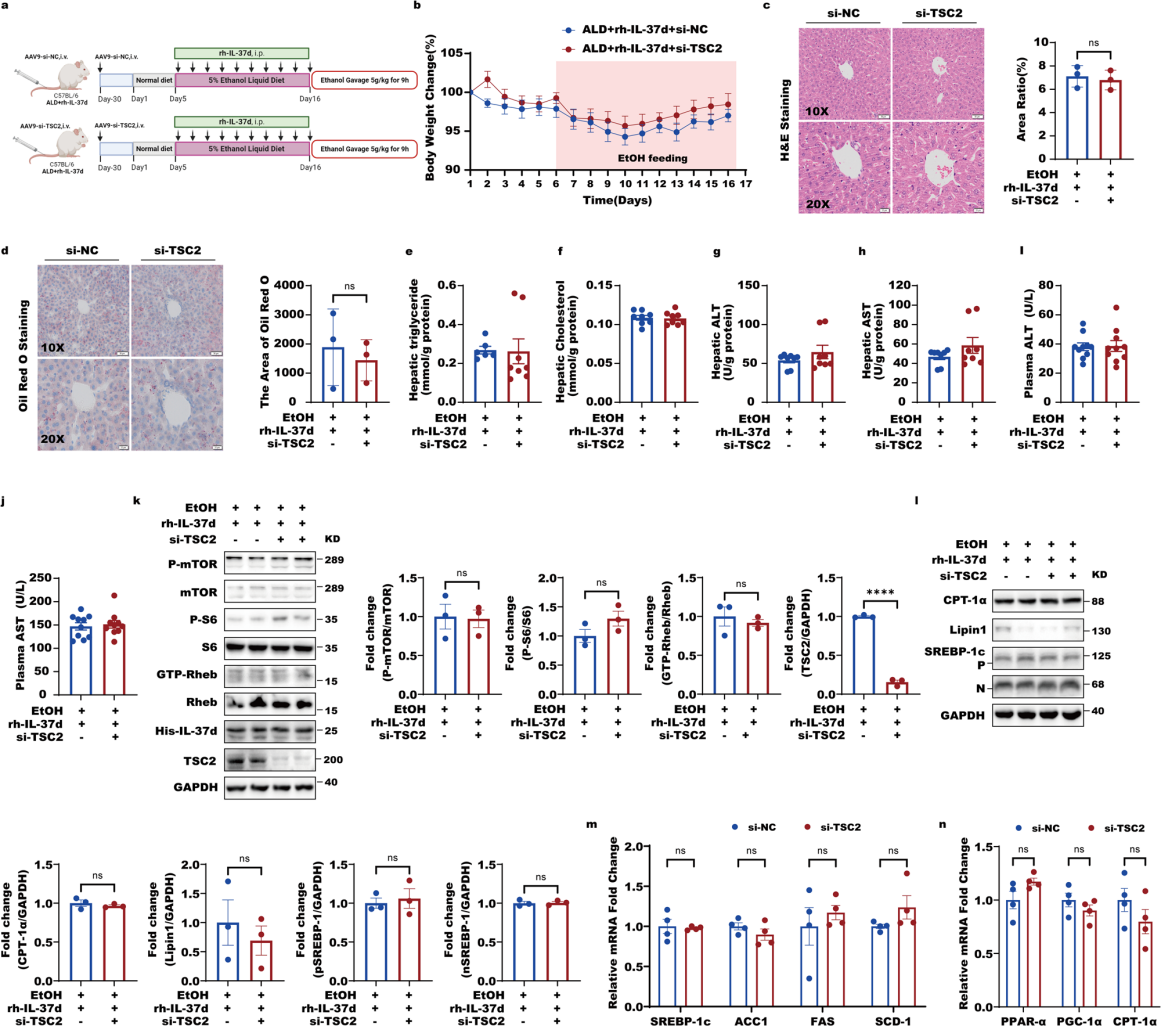
The protective effect of IL-37d in ALD relies on Rheb in a TSC2-independent manner in mice. **a** The timeline of the animal experiment showed that the downregulation of TSC2 had no effect on the therapeutic effect of rh-IL37d in ethanol-fed mice (The mouse images of the flowcharts were credited by Biorender.com). In detail, mice were given the AAV9 virus of si-Tsc2 or si-NC through tail vein injection one month before ethanol feeding. **b** The changes of body weight in groups of mice (*n* = 8–10) with or without small interference of Tsc2 (si-NC group or si-Tsc2 group). Data were shown as mean ± SEM (*n* = 8–10/group) and were analyzed using two-way ANOVA with Tukey’s multiple comparison test. **c** The graphs of H&E staining of liver sections in si-NC or si-Tsc2 groups (*n* = 3) and the area ratio was statistically performed. Data were shown as mean ± SEM (*n*  = 3/group) and were analyzed using unpaired two-tailed Student’s *t*-test. **d** The graphs of Oil Red O staining of the liver in si-NC or si-Tsc2 groups (*n* = 3) and the area of Oil Red O was statistically performed. Data were shown as mean ± SEM (*n*  = 3/group) and were analyzed using unpaired two-tailed Student’s *t*-test. **e**–**h** The liver function including activities of TG (**e**), total cholesterol (T-CHO) (**f**), ALT (**g**), and AST (**h**) were determined in mice of si-NC or si-Tsc2 groups. Data were shown as mean ± SEM (*n* = 6–8/group) and were analyzed using unpaired two-tailed Student’s *t*-test. **i**, **j** The levels of ALT (**i**) and AST (**j**) were analysed from mice of OE-NC and OE-Rheb groups. Data were shown as mean ± SEM (*n* = 10/group) and were analyzed using unpaired two-tailed Student’s *t*-test. **k** The liver lysates of mice from si-NC or si-Tsc2 groups were subjected to western blot with indicated antibodies. And the quantitative analyses of GTP-Rheb, p-S6, p-mTOR, and TSC2 were performed. Data were shown as mean ± SEM (*n* = 3/group) and were analyzed using unpaired two-tailed Student’s *t*-test. **l** The liver lysates of mice from si-NC or si-Tsc2 groups were subjected to western blot with indicated antibodies. And the quantitative analyses of nSREBP-1, pSREBP-1, Lipin1, and CPT-1A were performed. Data were shown as mean ± SEM (*n*  = 3/group) and were analyzed using unpaired two-tailed Student’s *t*-test. **m** The nuclear translocation of SREBP-1, as well as expression of SREBP-1c and its targets, including ACC1, FAS, and SCD1, were unaffected by downregulation of Tsc2 in chronic-binge ethanol-fed with rh-IL37d mice. Data were presented as mean ± SEM, *n* = 4–8. *P < 0.05 versus si-NC mice. Data were shown as mean ± SEM (*n* = 4/group) and were analyzed using one-way ANOVA with Tukey’s multiple comparison test. **n** The expressions of key genes involving fatty acid oxidation, such as PPARa, CPT-1a, and PGC-1a were analysed. Data were presented as mean ± SEM, *n* = 4–8. *P < 0.05 versus si-NC mice. Data were shown as mean ± SEM (*n* = 4/group) and were analyzed using one-way ANOVA with Tukey’s multiple comparison test.

In line with the observation in HepG2 that TSC2 level was not altered by alcohol, hepatic TSC2 levels remained unchanged in control and ALD mice in the absence or presence of rh-IL37d (Fig. 6k). When we silenced hepatic TSC2 in rh-IL37d treated ALD mice, the IL-37d improvements in ALD mice including the inhibition of body weight reductions, abnormal lipid accumulation in liver illustrated by H&E and Oil Red O staining and measurements of liver triglyceride and cholesterol levels, and the liver and plasma enzymatic activities of ALT and AST remained unchanged (Fig. 8a–j and Fig. S7a–d). The rh-IL37d-mediated suppression of Rheb-mTORC1 signaling, including GTP-Rheb, p-mTOR, and p-S6 in ALD mice was not influenced by hepatic TSC2 downregulation as well (Fig. 8k). The inhibitions of the key metabolic enzymes’ expressions for the synthesis of fatty acid and triglycerides by rh-IL37d including SREBP-1, ACC1, FAS, SCD1, and lipin 1 were neither affected by TSC2 silencing in the rh-IL37d treated ALD mice (Fig. 8l, m). Similarly, the elevations of expressions of lipid utilization and fatty acid oxidation genes, including CPT-1α, PPAR-α, and PGC-1α by the rh-IL37d remained unaltered in response to hepatic TSC2 silencing (Fig. 8l, n). These data support that the improvement of IL-37d in alcohol-induced hepatic steatosis is independent of TSC2. Collectively, the protective effect of the rh-IL37d in alcohol-induced hepatic steatosis is dependent on the suppression of Rheb activity in a TSC2-independent manner.

Together, hepatic IL37d is responsively decreased to promote ALD pathogenesis via the Rheb-mTORC1 axis. The rh-IL37d may thus be a therapeutic target to ameliorate abnormal lipid metabolism via negatively modulating the Rheb-mTORC1 axis in a TSC2-independent manner.

## Discussion

We here discover that IL-37d functions as a novel suppressor of Rheb independently of TSC2 to inhibit mTORC1 activation, thereby alleviating chronic alcohol-induced liver disorders. TSC2 has been well evidenced to serve as a GAP towards Rheb to promote the conversion of Rheb-GTP into Rheb-GDP, thereby restricting mTORC1 and its downstream targets activation. The identification of TSC2 in sensing and integrating nutrient signals and growth factors, like insulin, to regulate mTORC1 activity is huge of significance and provides the details of signal transduction and integration from external nutrition to intracellular growth and proliferation. At this point, TSC2 seems like a sensor of cell nutrients to tightly couple external nutritional signals with cell growth and proliferation. When insulin is present, TSC2 can be phosphorylated by AKT and disassociate from lysosomal Rheb to relieve its inhibition on mTORC1. Thus, TSC2 acts as a tumor suppressor to limit protein synthesis and cell growth. However, the mutations of either TSC2 or its partner, TSC1, often occur and cause persistent activations of mTORC1 and its targets, such as S6K and 4EBP1. The resulting overactivation of mTORC1 can induce diverse metabolic diseases and cancer. Although multiple mechanisms present to tightly control TSC2 activity, there are still plenty of clinical examples of TSC2 inability. Regarding this, other suppressors of Rheb needs urgent exploration. IL-37d is initially recognized as an anti-inflammatory cytokine but is ubiquitously expressed in various tissues and different types of non-immune cells, such as adipocyte and lung fibroblast. However, its function in these non-immune cells remains largely unknown. In our study, we identify that hepatic IL-37d acts as a negative regulator of Rheb, independently of TSC2. Therefore, this finding will propose IL-37d as an alternative target to prevent mTORC1-overactivation-induced diverse metabolic disorders and tumors. Especially for patients with TSC2 or TSC1 genetic mutations, IL-37d may be involved as a promising therapeutic target to treat severe developmental and neuronal disorders.

IL37d, like TSC2, is proved to suppress lysosomal Rheb activity without influencing its membrane localization. From our observations, the switch II subregion of Rheb, which is critical for maintaining structural stability in the GTP and GDP cycling during the hydrolysis process, mediates binding with IL-37d. The GTP-Rheb shows a higher affinity for IL-37d compared with GDP-Rheb. We, therefore, speculate that IL-37d may preferentially bind GTP-Rheb to suppress Rheb activity. As a known inhibitor of Rheb, TSC2 has been proved to simultaneously increase GTP and GDP binding, thereby accelerating its GTP hydrolysis and leading to the inhibition of Rheb activity. At this point, the molecular mechanism of IL-37d in negatively regulating Rheb activity is quite distinct from TSC2. Moreover, we observed that IL-37d can disassociate and capture Rheb from TSC2 but TSC2 doesn’t influence IL-37d-Rheb interaction. These data suggest that IL-37d binding may prevent TSC2 recruitment to Rheb and IL-37d thus displays a stronger inhibitory effect than TSC2. We should point out that, although a complete ORF for the mouse homolog of IL-37 has not been found, mouse and human Rheb is highly conserved and their protein sequence shared a 99% identity (Fig. S8a). Moreover, their structures also show the high similarity (Fig. S8b)^34,35^. The high identity of their sequence and structure supplies the basis of human IL-37d in the recognition and binding of both human and mouse Rheb. This would afford an explanation of the rh-IL37d effective function in ALD mouse in our study.

As previously reported, the binding ability of Rheb to mTOR is independent of its GTP- or GDP-status but its switch II segment plays a key role in promoting Rheb-mTOR direct binding^8,30^. We found that, distinct from TSC2, IL-37d disrupts interactions of Rheb-mTOR and mTOR-S6K, thereby further enhancing the suppression of mTORC1 via targeting the Rheb switch II subregion. Moreover, the switch II subregion of Rheb can also play a unique regulation of mTOR by recruiting other unknown coactivators, which is independent of Rheb activity and Rheb-mTOR interaction. Therefore, other IL-37 effects on mTORC1 needs further investigation. Another important thing we want to stress here is that what signal or molecule terminates mTORC1 signaling, which is a long-standing but quite difficult and complex question to answer, remains unsolved. Given that IL-37d has multiple inhibitory functions on mTORC1 via Rheb, we support that IL-37d acts as a novel terminator of mTORC1 activation.

Although extensive reports support that Rheb triggers cell growth via activating mTORC1, Rheb can also affect other targets in a mTORC1-independent way. Rheb interacts with Raf-1 and B-Raf kinases to suppress their activity under the control of TSC in a mTORC1-independent manner. However, the physiological significance of Rheb/Raf interaction remains vague. The association of Rheb and GAPDH has been reported to support a mTORC1-independent role of Rheb in regulating glycolytic flux. Their interaction can directly coordinate the mTORC1 signaling with the availability of glucose, thereby avoiding exhausting energy resources. In correlation with Rheb function in conservation of energy sources, Rheb has been evidenced to induce mitophagy by binding to mitochondrial autophagy receptor Nix (BNIP3L) and the autophagosome protein LC3-II. Their bindings link Rheb to mitochondria function in the maintenance of efficient energy production. Moreover, the dynamic translocation of Rheb to the mitochondrial matrix has been found. More importantly, mitochondrial Rheb can bind to and activate pyruvate dehydrogenase (PDH) independent of mTORC1, thereby mediating neuronal-activity-induced mitochondrial energetics. In addition to lysosomes, mitochondria, and cytoplasm, abundant Rheb can also localize or be activated in cellular Golgi, ER, and nucleus. Therefore, the role and relationship of IL-37d with Rheb function in an mTORC1-independent way and IL-37d-Rheb complex action in other subcellular compartments, such as Golgi, ER and nucleus are worthy of further investigation.

An important observation we noted is that hepatic IL-37d, rather than TSC2, is responsively reduced by alcohol, thereby relieving suppression of mTORC1 via Rheb. This finding indicates that IL-37d participates in liver injury and steatosis during chronic alcohol feeding. Chronic-plus-binge ethanol uptake can cause hyperactivations of mTORC1 and its downstream kinase S6K to promote ALD pathogenesis^33^. However, the key molecule leading to mTORC1 overactivation remains largely unknown. We here identify that hepatic IL-37d displays a crucial function in preventing ALD pathogenesis via negatively regulating the Rheb-mTORC1 axis. In addition to more than 70% hepatocyte, the Kupffer cells, macrophages that reside in the liver, also produce various cytokines to regulate the innate immune response in ALD development. Regarding this, it is reasonable to speculate that IL-37d, like other anti-inflammation cytokines, may exert its protective roles in ALD via inhibiting NF-κB activation and inflammation in the Kupffer cells. Consistently, the rh-IL37d recombinant protein showed a prominent efficacy in improving abnormal lipid metabolism in the liver via downregulating the Rheb-mTORC1 axis. IL-37d, thus, can systematically alleviate alcoholic liver injury and steatosis. To conclude, we support that IL-37d is physiologically involved in ALD development and the recombinant rh-IL37d protein may be a promising therapeutic approach to prevent or treat ALD.

We support that IL-37d can translocate to the lysosome membrane and target Rheb to modulate mTORC1. Our previous results demonstrate that mature IL-37b can bind directly to and inhibit RAC1, thereby inhibiting tumor metastasis^21^. Rac1 has been believed to bind and activate mTORC1 and thus promote cell growth. Therefore, whether IL-37d interacts with RAC1 to mediate mTORC1 regulation should be explored. TSC complex lysosome localization is dependent on membrane phosphatidylinositol (PIPs) and G3BP proteins, however, the molecular mechanisms by which it can tether on the lysosome surface remains elusive. Nevertheless, its lysosomal localization prompts us to further investigate its physiological functions, given that the lysosome is an important hub for integrating nutrients, stress, toxic stimulis, and infections of bacteria and virus. Our previous reports pointed out that IL-37d had a similar biological function with IL-37b by both extracellular and intracellular pathways. However, abundant cytoplasmic IL-37d function was still unclear. Our identification of IL-37d lysosomal localization here may open a new window to further explore the IL-37d function.

In summary, we here establish that IL-37d functions as a unique suppressor of Rheb to inactivate mTORC1 signaling independently of TSC2. Hepatic IL-37d is physiologically involved in ALD and the rh-IL37d protein can greatly improve alcohol-induced hepatic steatosis via negatively modulating Rheb-mTORC1 signaling in a TSC2-independent manner in mice. Overall, our findings suggest IL-37d as a novel repressor of Rheb independent of TSC2 and propose the possibility of putting forth rh-IL37d protein as a potential therapeutic strategy for ALD.

## Methods

### Mice

Eight- to ten-week-old male mice with body weight more than 20 g were used for the chronic-binge feeding experiments. The mice were housed in specific pathogen-free conditions on a 12 h light/dark cycle at 18–22 °C and allowed free access to sterilized food and water. All animal experiments were in accordance with the National Institutes of Health Guide for the Care and Use of Laboratory Animals and were approved by the Animal Care and Utilization Committee of Shandong University.

### Mice alcoholic liver model

Mice were divided randomly into ALD groups with or without 1 μg rh-IL37d daily i.p. injections and home cage controls with or without rh

-IL37d administration. An alcoholic liver injury mouse model was performed as described previously^36,37^. Briefly, mice were provided with chronic ethanol feeding (10-d ad libitum oral feeding with the Lieber-DeCarli ethanol liquid diet) plus a single binge ethanol feeding. The paired-fed control mice were given a calorie-matched maltodextrin diet. Control mice and ALD mice per group (*n* = 8–10) accepted vehicle treatment i.p. daily or 1 μg rh-IL37d recombinant protein during the whole experimental period. Mice received a single gavage of 5 g/kg body weight (31.5% vol/vol) or 45% maltodextrin for control mice, followed by 5% alcohol diet on day 16. Blood and liver samples were collected, processed immediately, and stored at −80 °C for further analyses^38,39^.

### Viral injection

For liver *tsc2* RNA interference, we injected 200 μl virus liquid (3 × 10^11^v.g./ml) carrying small interfering RNA targeting *tsc2* with TBG promoter by tail vein injection. For liver Rheb overexpression, we injected 200 μl virus liquid (3 × 10^11^ v.g.) containing OE-Rheb with TBG promoter by tail vein injection. The siRNA sequence for mouse *tsc2* was as listed: antisense, 5′-GGATGGATGTTGGCTTGTCCT-3′. The AAV9 recombinant virus of si-Tsc2 and si-NC were packaged and purified separately by Shanghai Genechem Co., Ltd (Shanghai, China). The AAV9 recombinant virus-containing OE-Rheb or control was derived from Shanghai Genechem Co., Ltd (Shanghai, China).

### Plasma collection

About 100 μl blood was collected in a collecting tube containing EDTA (Ethylene Diamine Tetra Acetic Acid) to avoid blood coagulation. Blood was mixed with EDTA by gently inverting the tube and centrifuged at 3500×*g* at 4 °C temperature for 15 min. Plasma was carefully aspirated and stored at −80 °C.

### ALT assay

Plasma ALT and Hepatic ALT levels were measured by the Alanine aminotransferase Assay Kit (Nanjing Jiancheng Bioengineering Institute) according to the manufacturer’s instructions.

### AST assay

Plasma AST and Hepatic AST levels were measured by the Aspartate aminotransferase Assay Kit (Nanjing Jiancheng Bioengineering Institute) according to the manufacturer’s instructions.

### TG assay

Hepatic TG level was measured by the Triglyceride Assay Kit (Nanjing Jiancheng Bioengineering Institute) according to the manufacturer’s instructions.

### TC assay

Hepatic TG level was measured by the Total cholesterol Assay Kit (Nanjing Jiancheng Bioengineering Institute) according to the manufacturer’s instructions.

### Cell culture

The human HCC cell line HepG2 and Huh7 were purchased from Shanghai Cell Bank of Chinese Academy of Sciences (Shanghai, China) and grown in Dulbecco’s Modified Eagle Medium (DMEM) and RPMI-1640 medium (Corning, MA, USA) with 10% fetal bovine serum (Gibco, CA, USA). Human lung cancer A549 cells and HEK-293T cells were from China Center for Type Culture Collection (Wuhan, China), and grown in Ham’s F12K medium (M&C GENE TECHNOLOGY, Ltd, Beijing, China) or DMEM with 10% fetal bovine serum. All the cells were incubated in a humidified incubator containing 5% CO_2_ at 37 °C.

For starvation, cells were incubated in Earle’s balanced salt solution (EBSS, Sigma) for 2 h, which was substituted for a complete medium to model cell starvation stress.

### Plasmid construction

The expression vector pEnCMV-RHOA(human)-3×HA, pEnCMV-HRAS(human)-FLAG-SV40-Neo, pEnCMV-RAB5A(human)-HA-SV40-Neo, PET-22b, pCDNA3.1-RHEB-Q64L-Myc, pCDNA3.1-RHEB-Q60K-Myc were purchased from Miaoling Biological (Wuhan, China). The expression vectors pBiFC-bFosVC155 and pBiFC-bJunVN173 were purchased from Addgene (USA). The expression vectors RHEB and TSC2 were purchased from Weizhen Biological (Shandong, China). The expression vector pBiFC-VC155, pBiFC-VN173, pBiFC-VC155-Rheb, pBiFC-VN173-IL-37d, pBiFC-VN173-IL-37d Δβ4, pBiFC-VN173-IL-37d Δβ5, pBiFC-VN173-IL-37d Δβ6, pBiFC-VN173-IL-37d Δα1 + 2, pBiFC-VN173-IL-37d Δβ10 + 11, pBiFC-VN173-IL-37d-A123P, pBiFC-VN173-IL-37d-G153D, pBiFC-VN173-IL-37d-C160, and pBiFC-VN173-IL-37d-N161S were constructed by Keyanyun biological.

For the construction of the pET-22b-TAT-his-IL-37d plasmid, we utilized the pCDNA3.1-C-his-IL-37d eukaryotic expression plasmid, which was previously constructed in the laboratory, as the template for PCR. The mature IL-37d (21–197aa) cDNA fused with TAT DNA coding sequences was then subcloned into the pET-22b plasmid with BamH I and Not I. The following primers were used: the forward primer: 5′-GGAATTAATTCGGATCCATGTACGGTCGTAAGAAACGTCGCCAGCGTCGCCGTGAACCCCAGTGCTGCTT-3′; The reverse primer: 5′-TGGTGCTCGAGTGCGGCCGCATCGCTGACCTCACTGGGGCTC-3′.

### IL-37d lentivirus construction

Full-length human IL-37d and Rheb were generated from the cDNA clone by PCR and cloned in frame with a 3 × Flag at the C-terminal into vector pcDNA3.1 or an N-terminal Myc into vector pCMV. All the IL-37d and Rheb mutants were constructed from IL-37d and Rheb plasmid using the KOD-Plus-Mutagenesis kit (Toyobo, Osaka, Japan). For the IL-37d expressing lentivirus, IL-37d cDNA with a 3× Flag tag at its C terminus was linked to the GV358 lentivirus vector and the GFP signal was used for evaluating infection efficiency (Genechem Co., Ltd.Shanghai, China). The titer of lentivirus is 5 × 10^8^ TU/ml.

### Transfection of plasmid and siRNA

Plasmid DNA was transiently transfected using Lipofectamine 2000 (Cat#11668-019, Invitrogen, USA) or jetPRIME (Polyplus, Illkirch, France). The experiments of siRNA transfection were carried out using INTERFERin (Cat#409-10, Polyplus, France). Cells were transfected at 70–80% confluence in serum-free Opti-MEM (Cat#31985-070, Gibco, USA). TSC2-specific siRNAs and control nonspecific siRNA were derived from Genepharma (Shanghai, China).

### Recombinant protein preparation and purification

The recombination protein of His-IL-37d was induced by isopropyl β-d-1 thiogalactopyranoside (IPTG) in *Escherichia coli* BL21(DE3) harboring pET-22b-TAT-IL-37d plasmid. The protein was purified as described before^40^. In brief, BL21(DE3) cells expressing (His)_6_-IL-37d was cultured overnight and 2.5 mL of the resulting cultures were transferred to 250 mL fresh LB medium individually. 1 mM IPTG was added to induce (His)_6_-IL-37d expression for 24 h at 16 °C when OD_600_ of the culture reached around 0.4. The bacterial cells were collected after being centrifuged for 30 min at 4000 rpm at 4 °C and resuspended with lysis buffer (25 mM Tris, 50 mM NaCl, PH = 8.0) or phosphate-buffered saline (PBS, 140 mM NaCl, 5 mM KH_2_PO_4_, 1 mM NaHCO_3_, PH 7.4) before lysis via sonication. For his-IL-37d affinity purification, cell lysates were added into 600 ul Ni-NTA column and washed with five column volumes of 30 mM imidazole to remove contaminated proteins. Finally, the his-IL-37d protein was eluted by elution buffer (250 mmol/L imidazole, 25 mmol/L Tris, PH = 8.0) and dialyzed to remove imidazole before use. SDS-PAGE and Coomassie Brilliant Blue (G-250) staining were used for determining the purification efficiency.

### Rheb activation assay and pull-down assay

Rheb was activated by the Rheb Activation Assay Kit (NewEast Biosciences) according to the manufacturer’s instructions.

For IL-37d and Rheb/GTP-Rheb pull-down assay, 1 ug of Rheb or GTP-Rheb protein were incubated with 200 ng of His-IL-37d in 1 ml lysis buffer followed by Ni-agarose beads incubation. After incubation at 4 °C overnight, the beads were washed three times to clear nonspecific binding proteins and then resuspended in 20 μL of SDS-PAGE sample buffer for western blot.

### Co-immunoprecipitation and pull-down assay

Cells were washed and lysed with ice-cold PBS and lysis buffer ((1.0% NP-40, 10 mM Tris-HCl, pH 7.4, 1 mM EDTA, 150 mM NaCl, 10% Glycerol, and protease inhibitor cocktail (Sigma)). The soluble fractions from cell lysates were isolated by centrifugation at 12,000 rpm for 20 min. Supernatants were incubated with anti-His magnetic beads (Sigma) for 6–8 h at 4 °C with rotation. Immunoprecipitants were washed five times with lysis buffer and denatured by 40 μl of 1 ×SDS sample buffer (Thermo, Rockford, USA) followed by SDS-PAGE and Western blot.

The recombination protein of His-IL-37d was induced by isopropyl β-d-1 thiogalactopyranoside (IPTG) in *Escherichia coli* BL21(DE3) harboring IL37d-pET28a plasmid. For IL-37d and Rheb pull-down assay, 200 ng of Rheb protein was incubated with 200 ng of His-IL-37d followed by Ni-agarose beads incubation in a binding buffer (50 mM Tris-HCl, pH 7.5, 1% Triton X-100, 150 mM NaCl, 1 mM dithiothreitol, 0.5 mM EDTA, 100 μM PMSF, 100 μM leupeptin, 1 μM aprotinin, 100 μM sodium orthovanadate, 100 μM sodium pyrophosphate, and 1 mM sodium fluoride) at 4 °C. The proteins with beads were boiled with SDS buffer prior to electrophoresis on SDS-polyacrylamide gels.

### Lysosome isolation

We separated the lysosome component using the Lysosome isolation kit (#BB31452, Bestbio). About 2 × 10^7^ cells were used and then rinsed three times with PBS buffer. Then cells were homogenized followed by lysosomes purification by gradient centrifugation.

### Quantitative real-time PCR

For quantitative real-time PCR analysis, total RNA was extracted by Trizol Reagent (Invitrogen) and reversely transcribed into cDNA using PrimeScript™ RT reagent Kit with gDNA Eraser (TAKARA, Japan). We used FastStartUniversal SYBR Green Master (Roche Applied Science, Penzberg, Germany) on the Bio-Rad CFX 96 (Bio-Rad, California, USA) to detect the expressions of genes by quantitative real-time PCR (q-PCR). The sequences of primers were shown in Table S4. The relative quantity of gene expression was normalized to β-actin or GAPDH expression in each sample.

### Western blot

The equal cell lysates were separated on sodium dodecyl sulfate-polyacrylamide gel and then transferred onto polyvinylidene fluoride membranes (Millipore, Billerica, MA, USA). 5% bovine serum albumin (BSA) in TBS containing 0.1% Tween-20 was used to block membranes for 2 h. The primary antibodies and secondary antibody conjugated with HRP were sequentially incubated with membranes. Then membranes were visualized by an ECL detection system (Sage Creation Science, Beijing, China). Primary antibodies: p-S6 (Ser235/236, Cell Signaling, 1:2000 rabbit), S6 (Cell Signaling, 1:1000 rabbit), p-S6K (Cell Signaling, 1:1000 rabbit), S6K (Cell Signaling, 1:1000 rabbit), P-mTOR (Cell Signaling, 1: 1000 rabbit),mTOR (Cell Signaling, 1: 1000 rabbit), TSC2(Santa, 1:1000 mouse), Rheb (New East, 1:1000 rabbit), GTP-Rheb (NewEast Biosciences, 1:1000 mouse), SREBP-1(BD, 1:1000 rabbit), Lipin1(CST, 1:1000 rabbit), CPT-1α (Abcam, 1:1000 mouse), Flag (Medical & Biological Laboratories, 1: 4000 mouse), His(ZSGB-Bio, 1: 2000 mouse), and GAPDH (ZSGB-Bio,1: 2000 mouse). HRP-conjugated anti-rabbit and anti-mouse IgG were purchased from Jackson ImmunoResearch (West Grove, PA, USA). The list of key resources used in this study were shown in the Supplementary Information file (Table S1), and the Original Blot images are included in the Supplementary Information file (Supplementary Fig. 9).

### Flow cytometry

Flow cytometry was used to evaluate the expressions of p-S6K and p-S6. For intracellular cytokine staining, cells were incubated in 100 µl p-S6K or p-S6 antibody (clone #4851, Cell Signaling Technology, Danvers, USA) diluent for 1 h. The dilution concentration of the antibody is 1:50. The flow cytometry analysis was performed by CytoFLEX S (Beckman, CA, USA). The resulting data were processed by FlowJo and CytExpert software.

Flow cytometry for cell death, after cell adherence, different concentrations of rh-IL37d were added 4 h in advance, and then ethanol of different concentrations was added for stimulation for 24 h. After treatment for 24 h, 0.25% trypsin was used to digest the single-cell suspension. Then FVD eFlour 660 dye solution was added for 30 min. The cells were centrifuged at 1000 rpm for 5 min and the pellets were collected and resuspended in a flow-up-like buffer solution for flow cytometry analysis.

### Immunofluorescence

Cells were seeded in 24 well plates on coverslips for 12 h and rinsed with phosphate-buffered saline (PBS). They were fixed in stationary liquid from Beyotime Biotechnology (Shanghai, China) for 10 min. After being blocked with 5% BSA in TBS-Tween buffer, Cells were sequentially incubated with primary antibodies and secondary antibodies. ProLong® Gold Antifade Mountant with DAPI (P36941, Life Technologies Corporation, USA) was used to stain nuclei. Images were captured with a Carl Zeiss LSM780 confocal microscope (Oberkochen, Germany) and images were exported from ZEN software. Primary antibodies: p-S6 (Ser235/236, Cell Signaling, 1:400 rabbit), Rheb (Cell Signaling, 1:200 rabbit), GTP-Rheb (NewEast Biosciences, 1:100 mouse), and Flag (Medical & Biological Laboratories, 1: 4000 mouse). The secondary antibodies: Alexa Fluor 647 (mouse/rabbit), Alexa Fluor 594 (mouse/rabbit), and Alexa Fluor 488(mouse/rabbit) (Abcam, Cambridge, UK, 1:500).

### Immunohistochemistry

The liver tissue of mice from the ALD mouse model was used for the detection of p-S6 expression. The slides were pretreated in citrate buffer (pH 6.0) in a microwave oven for 15 min and washed in distilled water, followed by 3% H_2_O_2_ treatment for 10 min. The p-S6 (Ser235/236, Cell Signaling, 1:400 rabbit) antibody were then incubated with the slide. Horseradish peroxidase-conjugated anti-rabbit IgG and 3,5-diaminobenzidine peroxidase Substrate Kit (Maixin Co., Fuzhou, China) were used to stain the slides, followed by counterstaining with Mayer’s hematoxylin.

### Bimolecular fluorescent complimentary (BiFC) assay

This assay is used for detecting the rapid visualization of the compartment-specific interactions of a protein complex, and the protein–protein interactions can be easily quantified in living cells. BiFC expression plasmids for IL-37d and Rheb were constructed by inserting the PCR fragment containing full-length IL-37d, Rheb, or their derivatives into pBiFC-VN173 and pBiFC-VC155. The resulting plasmids were transfected into HEK-293T cells and co-transfections of pBiFC-bFosVC155 and pBiFC-bJunVN173 were defined as a positive control. DAPI stain was indicated as cellular nuclear. The BiFC signals of YFP fluorescence were detected at 561 nm excitation wavelength by LSM780 with a 63 x Plan-Apochromat objective and the resulting data and image were analyzed using ZEN lite 2012 software package.

### Biolayer interferometry (BLI)

BLI is a widely recognized biological method for studying biomolecular interactions^41–43^. Assays were performed on an Octet R4 (ForteBio) instrument at 30 °C with shaking at 1000 RPM. The anti-penta GST biosensor is incubated in PBST (PBS + 0.02% Tween-20) buffer for 10 min. GST-Rheb protein was loaded at 5 μg/mL in PBST Buffer for 200 s prior to baseline equilibration. The association of IL-37d protein in PBST buffer at various concentrations in a two-fold dilution series from 1000 nM to 62.5 nM was carried out for 180 s prior to dissociation for 180 s. The data were baseline subtracted prior to fitting performed using a 1:1 binding model and the ForteBio data analysis software. Mean kon and koff values were determined with a global fit applied to all data.

### CCK8

About 10,000 cells were inoculated into 96-well plates and treated with different concentrations of ethanol and rh-IL37d for 24 h after adherent to the walls. After the culture-medium was discarded, 10 μL CCK8 dye and 90 μL PBS were added and incubated in 37 °C incubator for 90 min. The absorbance was detected by absorbance reader.

### Oil Red O staining

Fresh frozen fixed liver sections were immersed with Oil Red solution for 8–10 min in the dark, and then differentiated with 60% isopropanol, washed with pure water and mounted with glycerol gelatin after hematoxylin stain. The full sections were ready for scanning by Panoramic Scanning Microscope-VS120 (Olympus Life Science, Tokyo, Japan).

HEPG2 cells after pretreatment were fixed in 4% PFA for 10 min, incubated with 60% isopropanol for 15–20 s, and stained by Oil Red O followed by 60% isopropanol treatment for 3–5 s. The cells were then observed under an inverted microscope (Olympus, Japan).

### Molecular docking

To assess the possibility of IL-37d and Rheb interactions, studies to predict their interactions were carried out by Discovery Studio 2016 client software. The modules MODELER, Docking (ZDOCK/RDOCK) Proteins, and Analyzing interaction, were used to investigate the interactions of IL-37d (5HN1, PDB) and Rheb with GTP or GDP (1XTS and 1XTQ, PDB). The three-dimensional structures of IL-37d and Rheb were obtained from the RCSB. PDB Protein Data Bank (http://www.rcsb.org/pdb/home/home.do). The hydrophobic binding, hydrogen bonding, and Pi interactions of docked molecules were calculated.

### Quantification analysis

The specific western band signals were quantificationally analyzed by Image J. All image statistical analyses were done by Image Pro-Plus and Zeiss Auto-measure software.

### Statistics and reproducibility

Mean values, standard error of the mean, and statistical significance were calculated by GraphPad Prism 9. Student’s *t*-test (unpaired), one-way or two-way ANOVA followed by Tukey’s test (for multiple comparisons) were used to analyze the differences between groups. *P* values <0.05 standard for being statistically significant; *P < 0.05, **P < 0.01, ***P < 0.001, and ****P < 0.0001. At least three independent experiments of biological replicates were calculated.

### Materials availability

This work did not generate new unique reagents.

### Reporting summary

Further information on research design is available in the Nature Portfolio Reporting Summary linked to this article.

### Supplementary information

Supplementary Information

Description of Additional Supplementary Files

Supplementary Data

Reporting Summary

## Data Availability

Data from this study are available on request and numerical source data underlying all graphs in the manuscript can be found in the supplementary data file.
