# Supplementary material for: IL-37d suppresses Rheb-mTORC1 axis independently of TCS2 to alleviate alcoholic liver disease
Source: Commun Biol. 2024 Jun 21;7:756. doi: 10.1038/s42003-024-06427-8 (PMC11192940; doi:10.1038/s42003-024-06427-8)
Supplement: Supplementary file 1 — Supplementary Information [file 42003_2024_6427_MOESM1_ESM.pdf]

Supplementary Information

IL-37d suppresses Rheb-mTORC1 axis independently of TCS2 to alleviate alcoholic liver disease

Nuo Chen[1], Xiaoyu Wang[1], Yaxin Guo[1], Ming Zhao[1], Baihui Cao [1], Bing Zhan[1], Yubin Li[1], Tian Zhou[1], Faliang Zhu[1], Chun Guo[1], Yongyu Shi[1], Qun Wang[1], Lining Zhang[1]* and Yan Li[2*]

1 Department of Immunology, School of Basic Medical Science, Shandong University, Jinan, China

2 Department of Pathogen Biology, School of Basic Medical Science, Shandong University, Jinan, China

*Reprints and correspondence to:

Yan Li, PhD, Department of Pathogen Biology, School of Basic Medical Science, Shandong University, Jinan, China (E-mail: liyan2015@sdu.edu.cn);

Lining Zhang, MD, PhD, Department of Immunology, School of Basic Medical Science, Shandong University, 44# Wenhua Xi Road, Jinan 250012, China (E-mail: zhanglining@sdu.edu.cn).

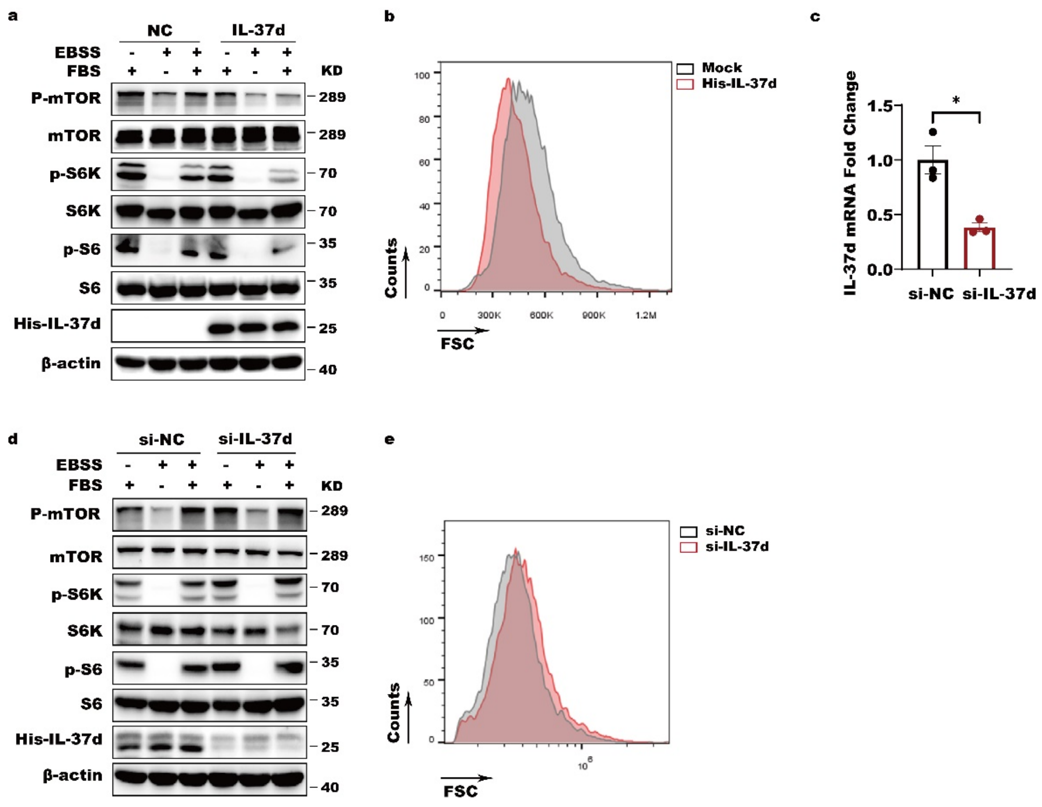

Figure S1. IL-37d negatively regulates mTORC1 activity by Rheb. (a) A549 cells were transfected with IL-37d plasmid or control plasmid, respectively. The cells were starved for 2 hours in EBSS medium and then activated with F12K medium containing 10% FBS for 20 minutes followed by western blot with indicated antibodies. **(b)** A549 cells were transfected with IL-37d plasmid or control plasmid, respectively. The cells were transfection for 24h followed by flow cytometry analysis for FSC. **(c)** Quantitative real-time PCR (qRT-PCR) analysis of IL-37d expression levels in HepG2 cells exposure to si-NC and si-IL-37d. Data were normalized with GAPDH mRNA levels and presented as fold changes compared with control group. Data were shown as mean ± SEM (n =3/group) and were analyzed using unpaired two-tailed Student's *t* test, *P <0.05. **(d)** HepG2 cells were infected by small RNAs against control (si-NC) or IL-37d (si-IL-37d), respectively. The transfected cells were starved for 2 hours in EBSS medium and then activated with F12K medium containing 10% FBS for 20 minutes followed by western blot with indicated antibodies. **(e)** HepG2 cells were infected by small RNAs against control (si-NC) or IL-37d (si-IL-37d), respectively. The cells were transfection for 48h followed by flow cytometry analysis for FSC.

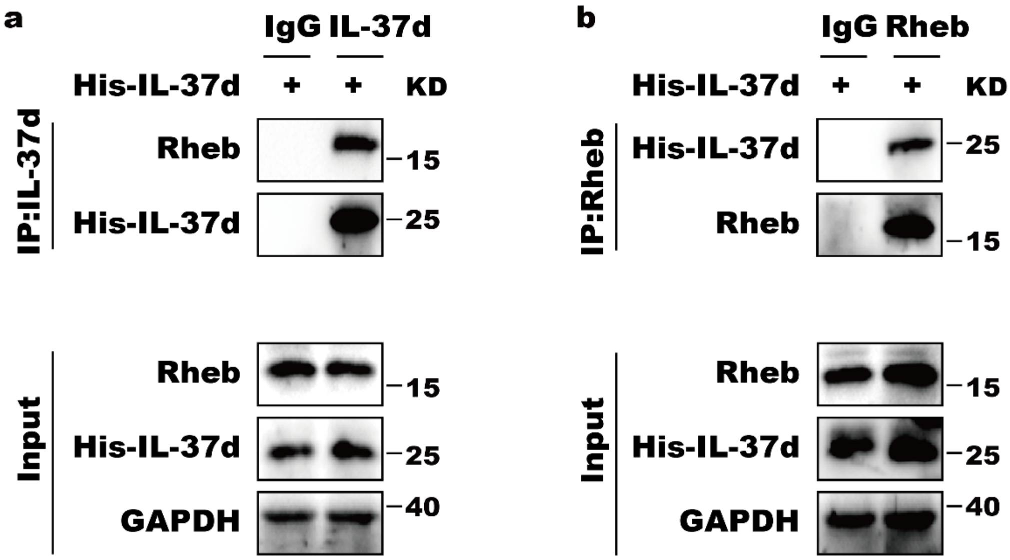

Figure S2. IL-37d targets lysosomal Rheb to inhibit its activity. (a) HepG2 cells were transfected with IL-37d plasmid. The whole cell lysates were incubated with IL-37d antibody. Then the protein A/G were added and the cell pellets were subjected to western blot with His antibodies. **(b)** HepG2 cells were transfected with IL-37d plasmid. The whole cell lysates were incubated with Rheb antibody. Then the protein A/G were added and the cell pellets were subjected to western blot with Rheb antibodies.

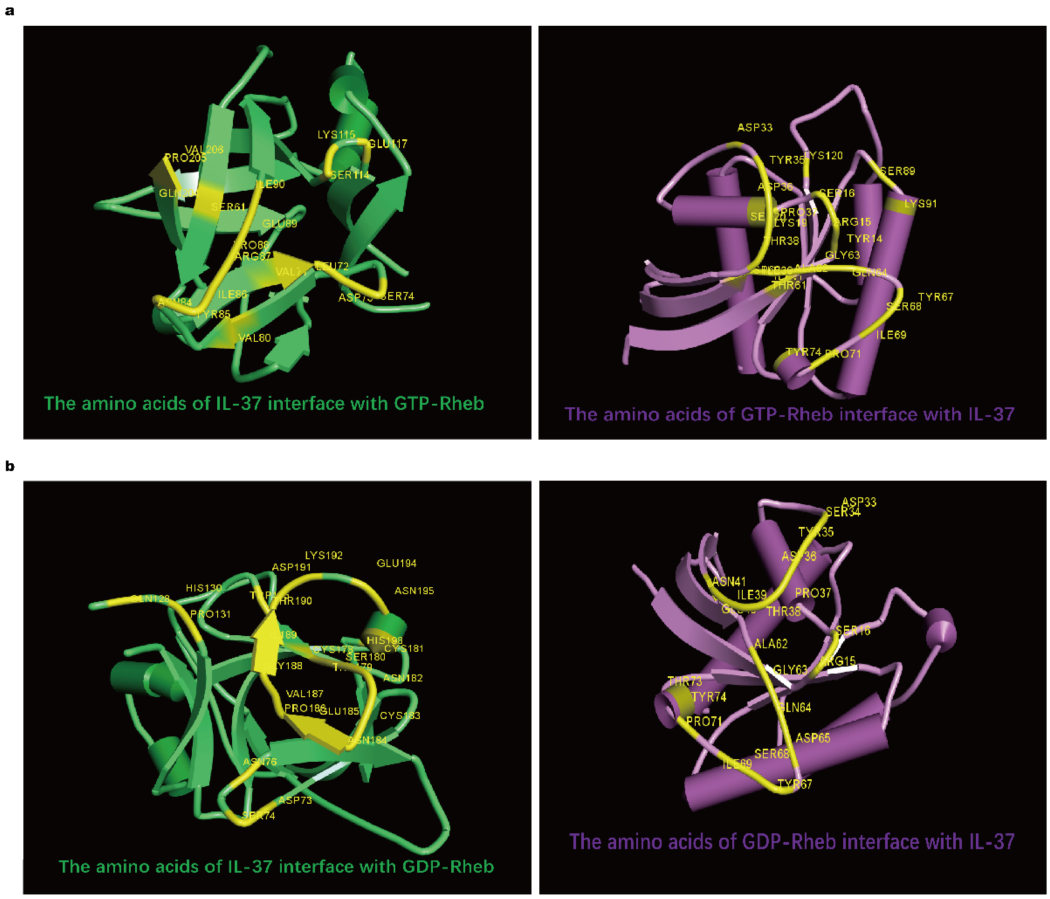

Figure S3. The β-trefoil fold of IL37d is required for binding to switch II motif of Rheb. (a) The amino acid compositions of IL-37 in the interface with GTP-bound Rheb; The amino acid compositions of GTP-Rheb in the interface with IL-37. **(b)** The amino acid compositions of IL-37 in the interface with GDP-bound Rheb; The amino acid compositions of GDP-Rheb in the interface with IL-37.

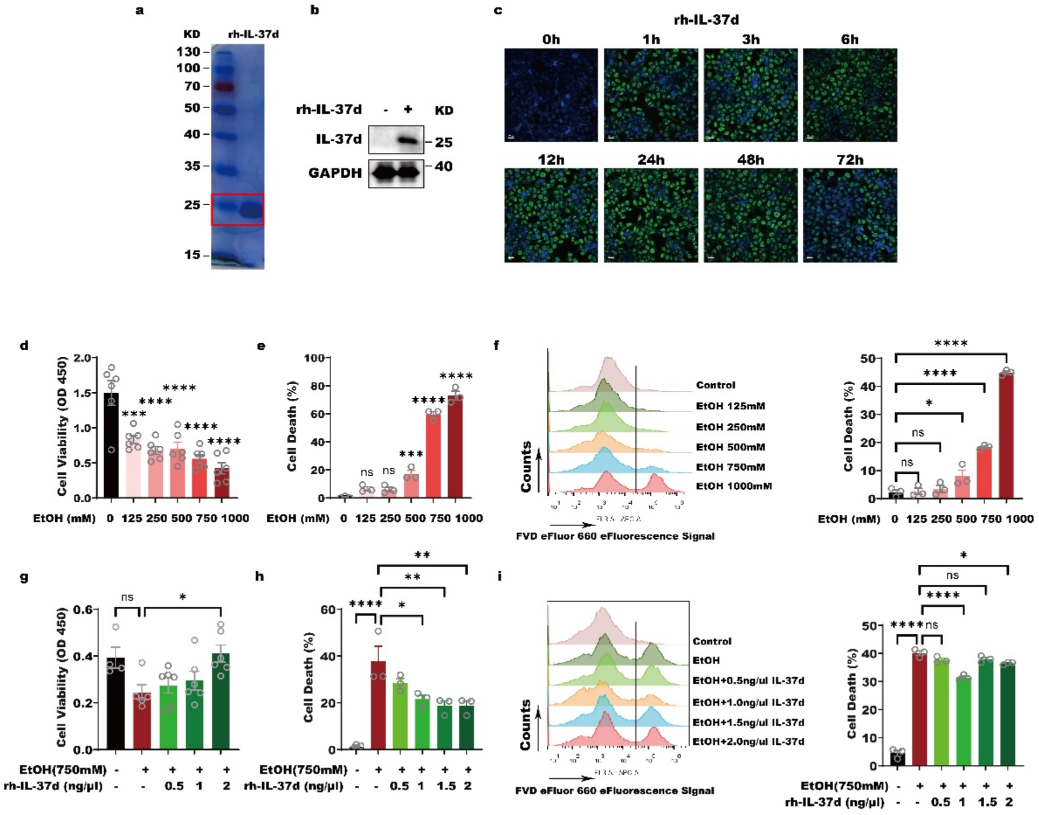

Figure S4. Alcohol downregulates IL-37d to activate mTOR via relieving suppression of Rheb. (a) The recombinant protein of human IL-37d with His tag was purified from Rosetta transfected with pET-22b-IL-37d plasmids in the presence of 1 mM IPTG by Ni agarose column. The expression of rh-IL-37d was confirmed by SDS-PAGE. **(b)** HepG2 cells were incubated with 500 ng/mL rh-IL37d protein for 24h, the expression of rh-IL-37d was confirmed by western blot with the anti-IL-37d antibody. **(c)** HepG2 cells were incubated with 500 ng/mL rh-IL37d protein for indicated times. Then cells were fixed and immune-stained with his tag antibody and DAPI to examine the status of intracellular rh-IL37d. **(d)** Huh7 cells were treated with or without increasing alcohol from 125 mmol to 1000 mmol. At 24h treatment, the absorbance was detected by Absorbance Reader on OD450. Data were shown as mean ± SEM (n =6 biological replicates) and were analyzed using one-way ANOVA with Tukey's multiple comparison test, ***P <0.001, ****P <0.0001. **(e)** Huh7 cells were treated with or without increasing alcohol from 125 mmol to 1000 mmol. At 24h treatment, the number of dead cells was recorded by Trypan blue staining. Data were shown as mean ± SEM (n =3 biological replicates) and were analyzed using one-way

ANOVA with Tukey's multiple comparison test, ***P <0.001, ****P <0.0001. **(f)** Huh7 cells were treated with or without increasing alcohol from 125 mmol to 1000 mmol. At 24h treatment, dead cells were stained with eBioscience™ Fixable Viability Dye eFluor™ 660 and the number of dead cells was determined by flow cytometry. Data were shown as mean ± SEM (n =3 biological replicates) and were analyzed using one-way ANOVA with Tukey's multiple comparison test, *P <0.05, ****P <0.0001. **(g)** Huh7 cells were treated with or without increasing alcohol from 125 mmol to 1000 mmol. In the treatment group, different concentrations of rh-IL37d were added 4h in advance. After ethanol stimulation for 24 hours, the absorbance was detected by Absorbance Reader on OD450. Data were shown as mean ± SEM (n =4-6 biological replicates) and were analyzed using one-way ANOVA with Tukey's multiple comparison test, *P <0.05. **(h)** Huh7 cells were treated with or without increasing alcohol from 125 mmol to 1000 mmol. In the treatment group, different concentrations of rh-IL37d were added 4h in advance. After ethanol stimulation for 24 hours, the number of dead cells was recorded by Trypan blue staining. Data were shown as mean ± SEM (n =3 biological replicates) and were analyzed using one-way ANOVA with Tukey's multiple comparison test, *P <0.05, **P <0.01, ****P <0.0001. **(i)** Huh7 cells were treated with or without increasing alcohol from 125 mmol to 1000 mmol. In the treatment group, different concentrations of rh-IL37d were added 4h in advance. After ethanol stimulation for 24 hours, dead cells were stained with eBioscience™ Fixable Viability Dye eFluor™ 660 and the number of dead cells was determined by flow cytometry. Data were shown as mean ± SEM (n =3 biological replicates) and were analyzed using one-way ANOVA with Tukey's multiple comparison test, *P <0.05, ****P <0.0001.

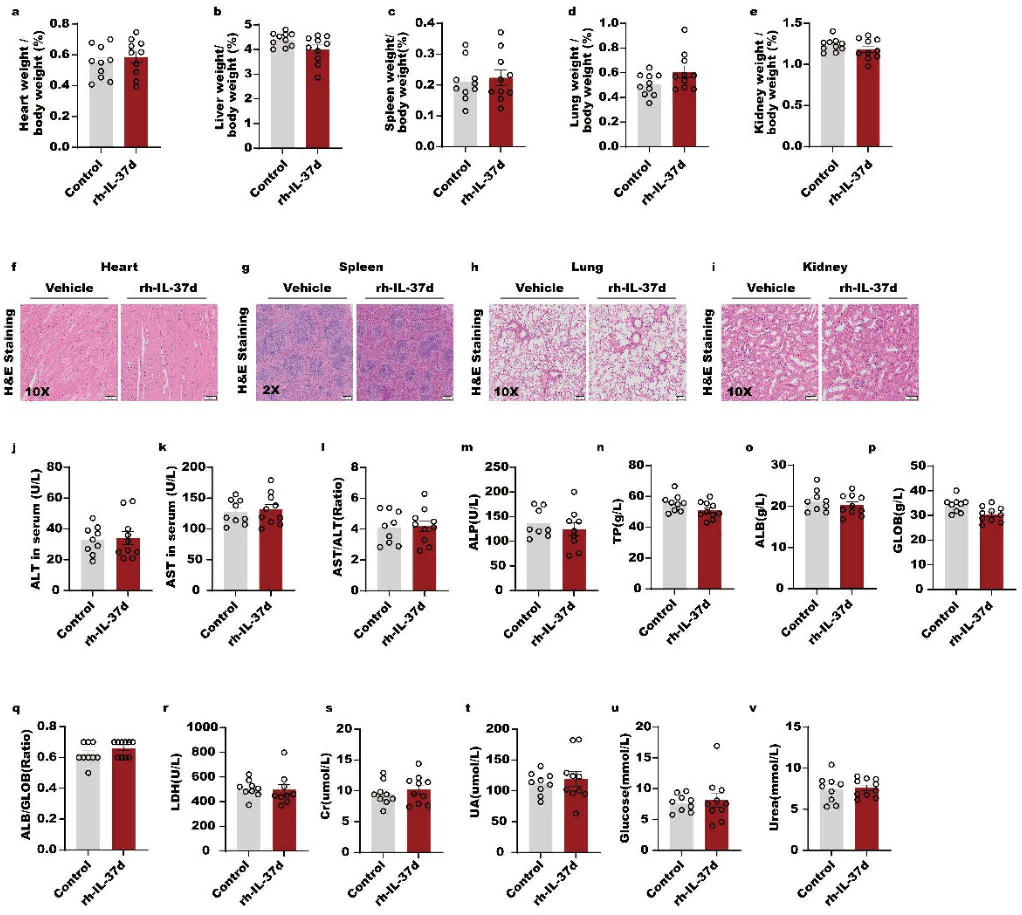

Figure S5. **IL-37d suppresses alcohol-induced mTORC1 overactivation and hepatic steatosis in chronic-binge ethanol-fed mice**. **(a-e)**The weights of heart **(a)**, liver **(b)**, spleen **(c)**, lung **(d)** and kidney **(e)** were not influenced by rh-IL37d. **(f-i)** The morphology of heart **(f)**, spleen **(g)**, lung **(h)** and kidney **(i)** were not influenced by rh-IL37d. **(j-r)** The levels of plasma alanine aminotransferase (ALT) **(j)**, aspartate aminotransferase (AST) **(k)**, aspartate aminotransferase/alanine aminotransferase (S/L) **(l)**, alkaline phosphatase (ALP) **(m)**, total protein (TP) **(n)**, albumin (ALB) **(o)**, globulin (GloB) **(p)**, albumin/ globulin (A/G) **(q)** and lactate dehydrogenase (LDH) **(r)** were neither altered by rh-IL37d, suggesting that IL-37d has no toxic effects on liver function in normal mice. **(s-v)** The kidney function related-Crea (Cr) **(s),** uric acid (UA) **(t)**, glucose (GLU) **(u)** and Urea **(v)** levels were not influenced by rh-IL37d. Data were shown as mean ± SEM (n =9-10/group) and were analyzed using unpaired two-tailed Student's *t* test.

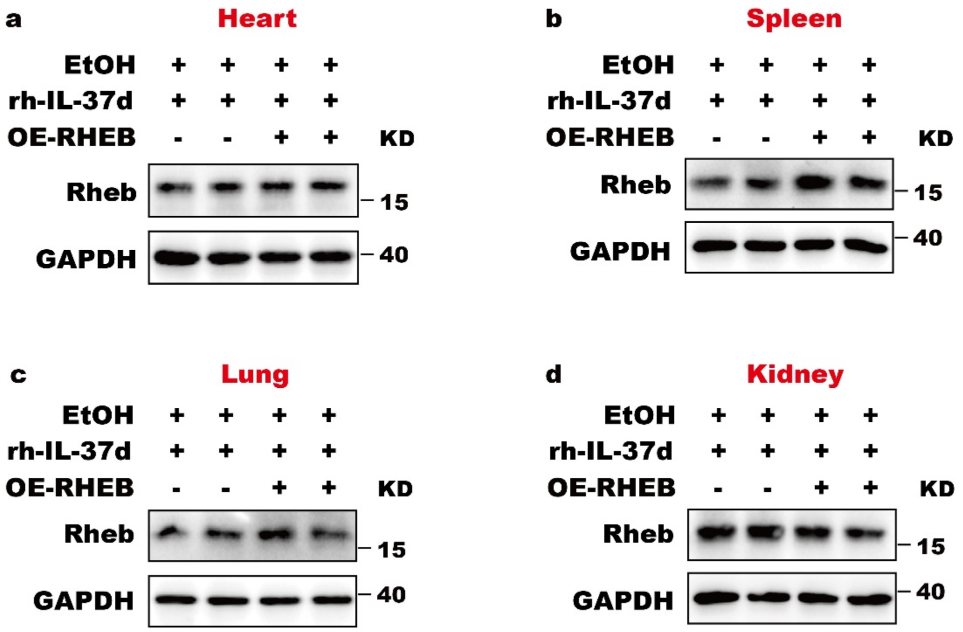

Figure S6. **The protective effect of IL-37d in ALD relies on Rheb. (a-d)** Adeno-associated virus 9 (AAV9)-mediated hepatic Rheb overexpression derived by the thyroxine binding globulin (TBG) promoter through tail vein injection had no effect to the expressions of Rheb in Heart **(a)**, Spleen **(b)**, Lung **(c)** and Kidney **(d)**.

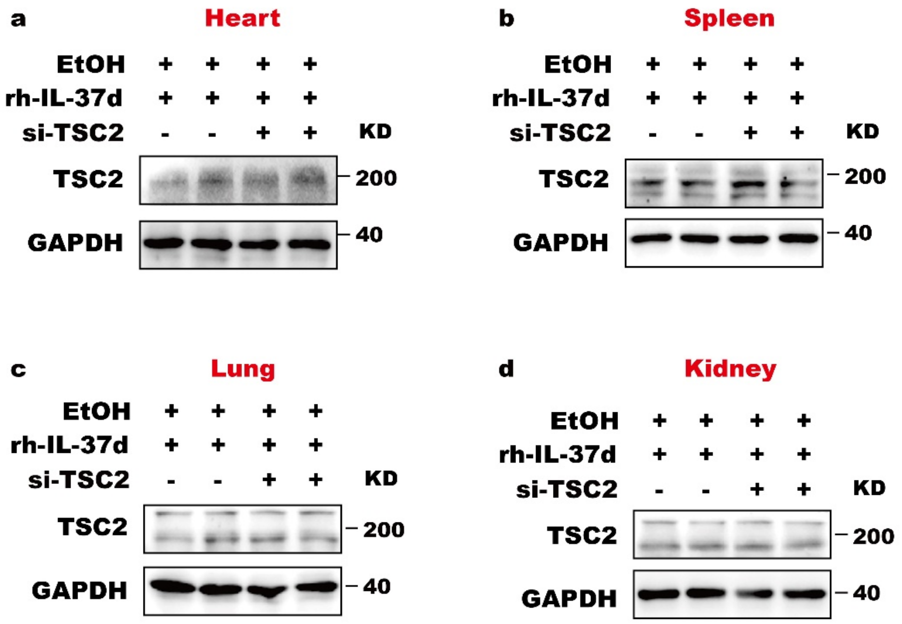

Figure S7. The protective effect of IL-37d in ALD relies on Rheb in a TSC2-independent manner in mice. (a-d) Adeno-associated virus 9 (AAV9)-mediated hepatic TSC2 knockdown derived by the thyroxine binding globulin (TBG) promoter through tail vein injection had no effect to the expressions of TSC2 in Heart **(a)**, Spleen **(b)**, Lung **(c)** and Kidney **(d)**.

a

```
Query: NP_444305.2 GTP-binding protein Rheb [Mus musculus] Query ID:
lcl|Query_8575 Length: 184

>NP_005605.1 GTP-binding protein Rheb [Homo sapiens]
Sequence ID: Query_8577 Length: 184
Range 1: 1 to 184

Score:375 bits(962), Expect:5e-140,
Method:Compositional matrix adjust.,
Identities:182/184(99%), Positives:184/184(100%), Gaps:0/184(0%)

Query  1
MPQSKSRKIAILGYRSVGKSSLTIQFVEGQFVDSYDPTIENTFTKLITVNGQEYHLQLVD  60

MPQSKSRKIAILGYRSVGKSSLTIQFVEGQFVDSYDPTIENTFTKLITVNGQEYHLQLVD
Sbjct  1
MPQSKSRKIAILGYRSVGKSSLTIQFVEGQFVDSYDPTIENTFTKLITVNGQEYHLQLVD  60

Query  61
TAGQDEYSIFPQTYSIDINGYILVYSVTSIKSFEVIKVIHGKLLDMVGKVQIPIMLVGNK  120

TAGQDEYSIFPQTYSIDINGYILVYSVTSIKSFEVIKVIHGKLLDMVGKVQIPIMLVGNK
Sbjct  61
TAGQDEYSIFPQTYSIDINGYILVYSVTSIKSFEVIKVIHGKLLDMVGKVQIPIMLVGNK  120

Query  121
KDLHMERVISYEEGKALAESWNAAFLESSAKENQTAVDVFKRIILEAEKIDGAASQGKSS  180

KDLHMERVISYEEGKALAESWNAAFLESSAKENQTAVDVF+RIILEAEK+DGAASQGKSS
Sbjct  121
KDLHMERVISYEEGKALAESWNAAFLESSAKENQTAVDVFRRIILEAEKMDGAASQGKSS  180

Query  181  CSVM  184
            CSVM
Sbjct  181  CSVM  184
```

b

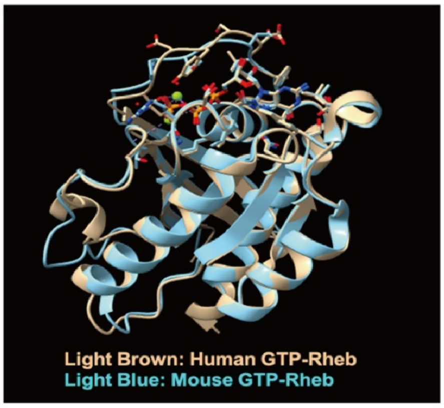

Figure S8. The protein sequences and structures of mouse and human Rheb. (a) The protein sequences of mouse and human Rheb. **(b)** The structures of mouse and human Rheb.

Figure S9– Original Blots

Relevant areas for cropped blots in the main and Extended Data figures are shown with a solid box.

Supplemental Material to Fig 1a (original blots)

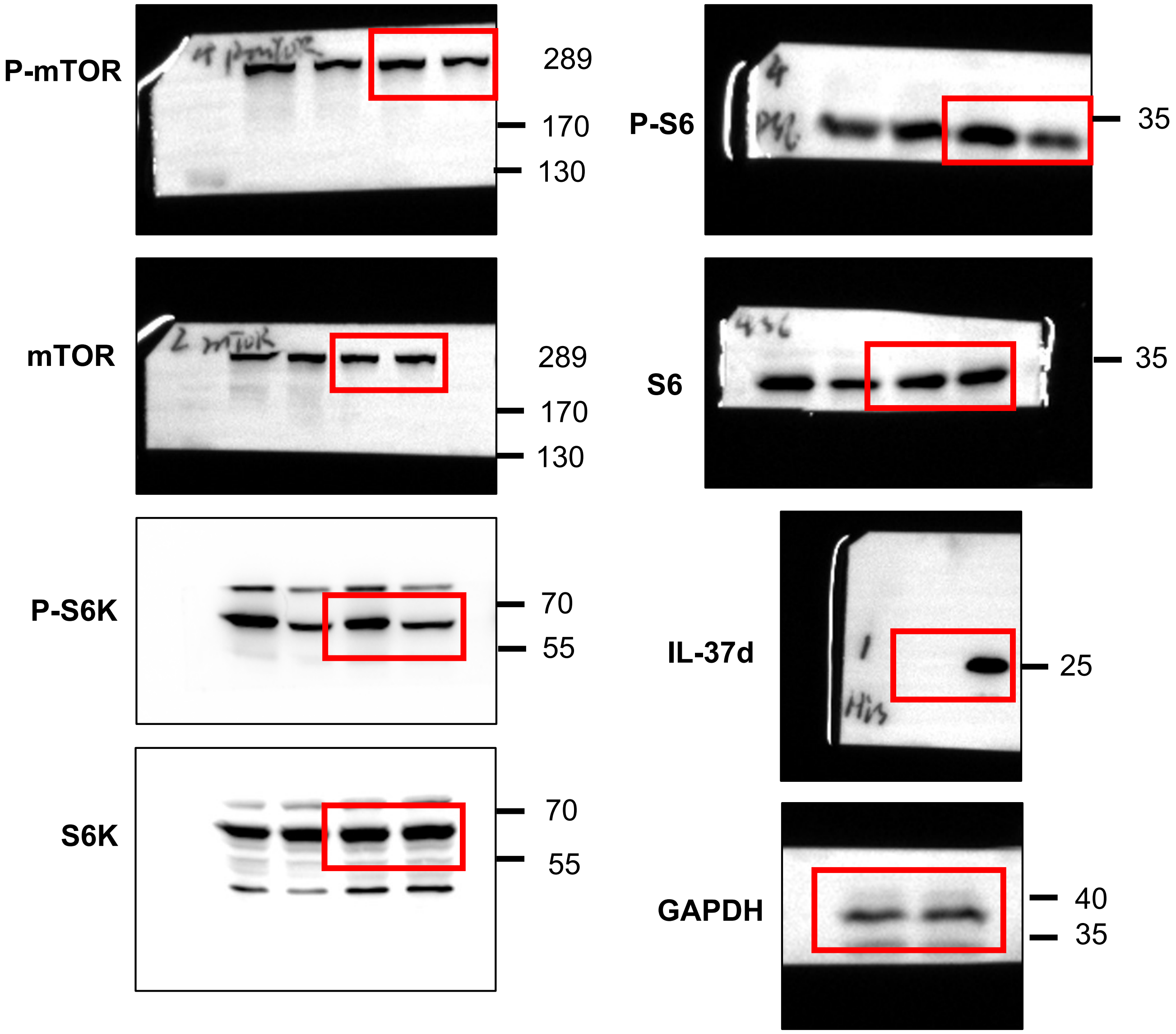

Supplemental Material to Fig 1e (original blots)

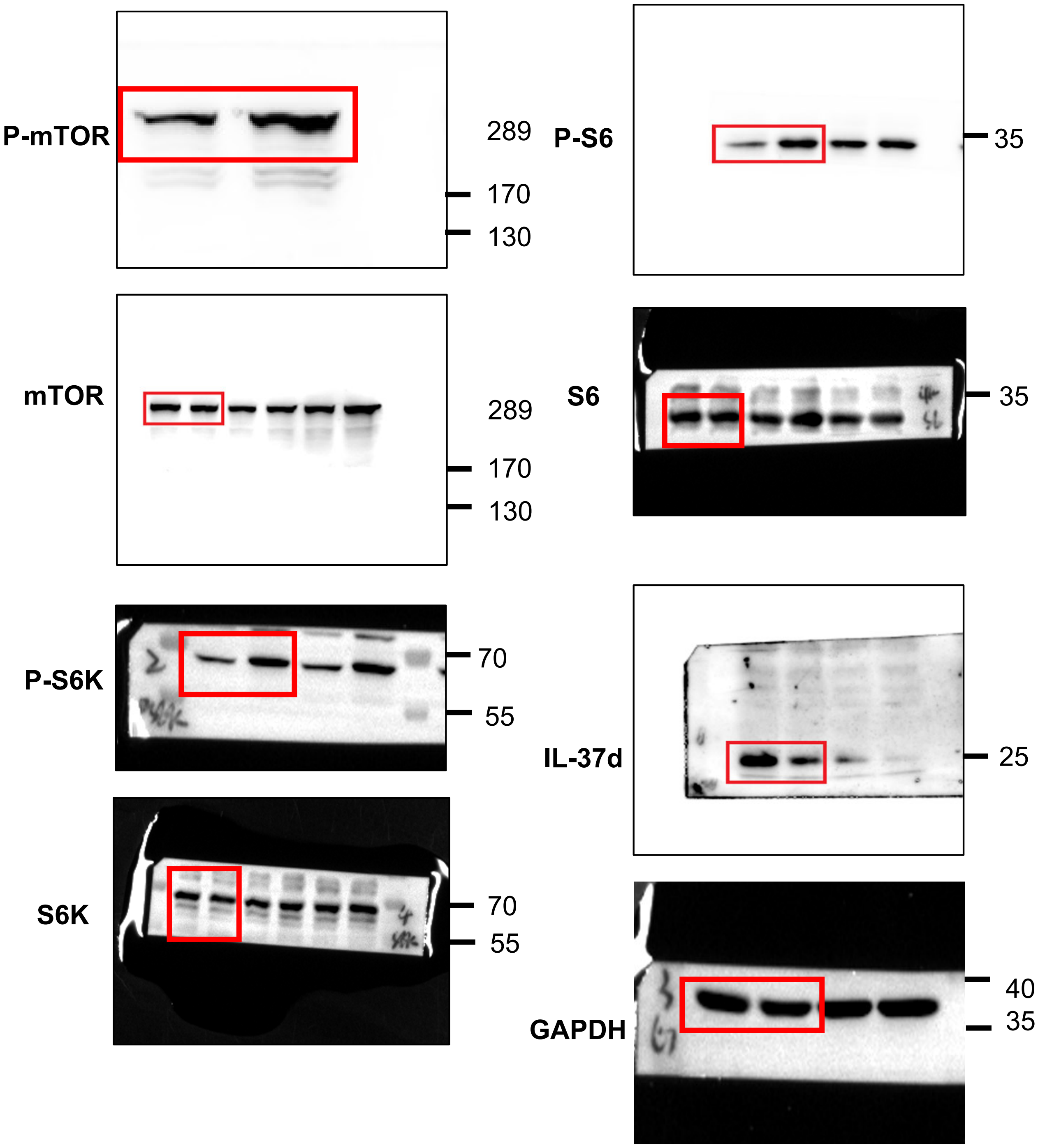

Supplemental Material to Fig 1i-j (original blots)

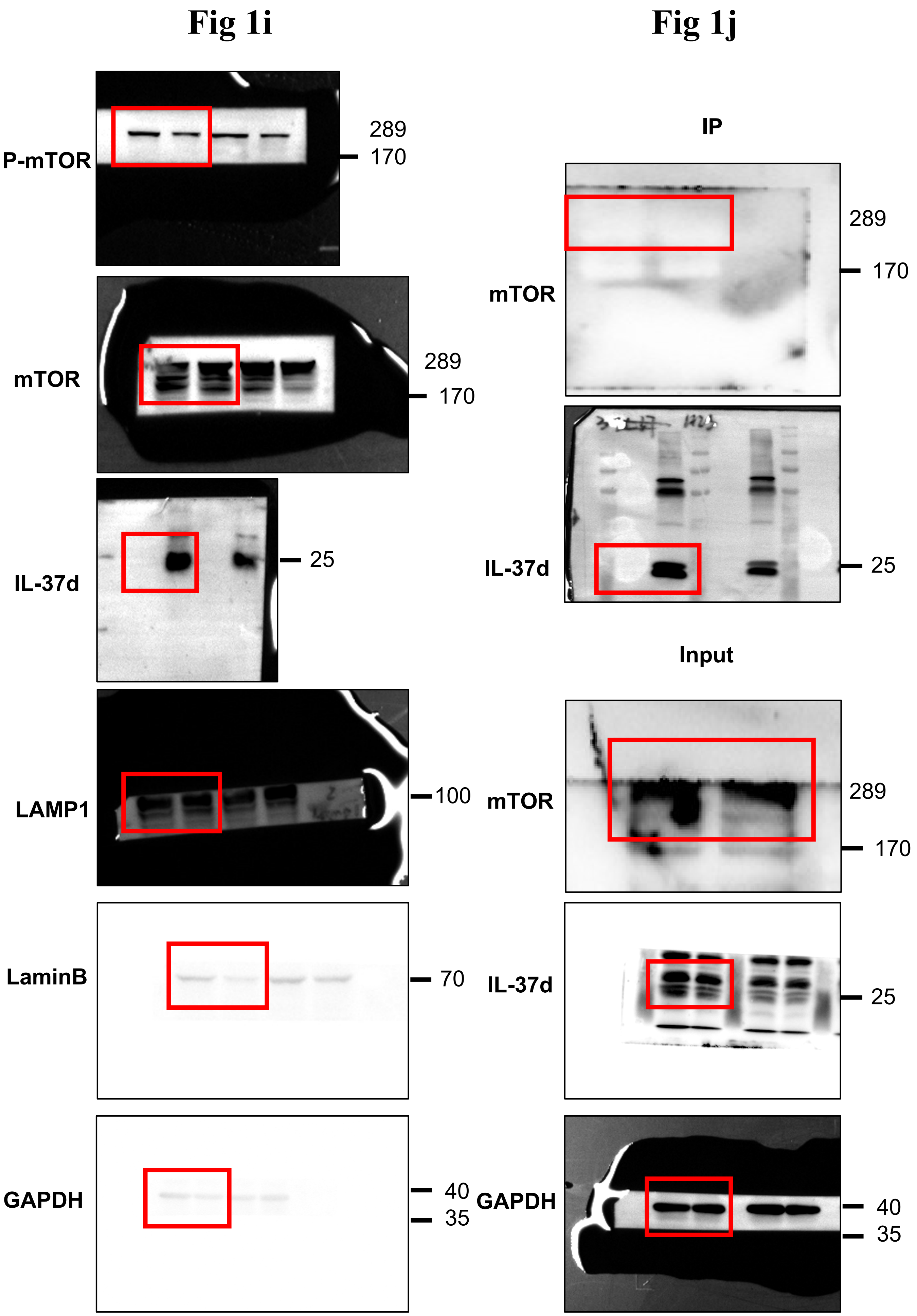

Supplemental Material to Fig 1k (original blots)

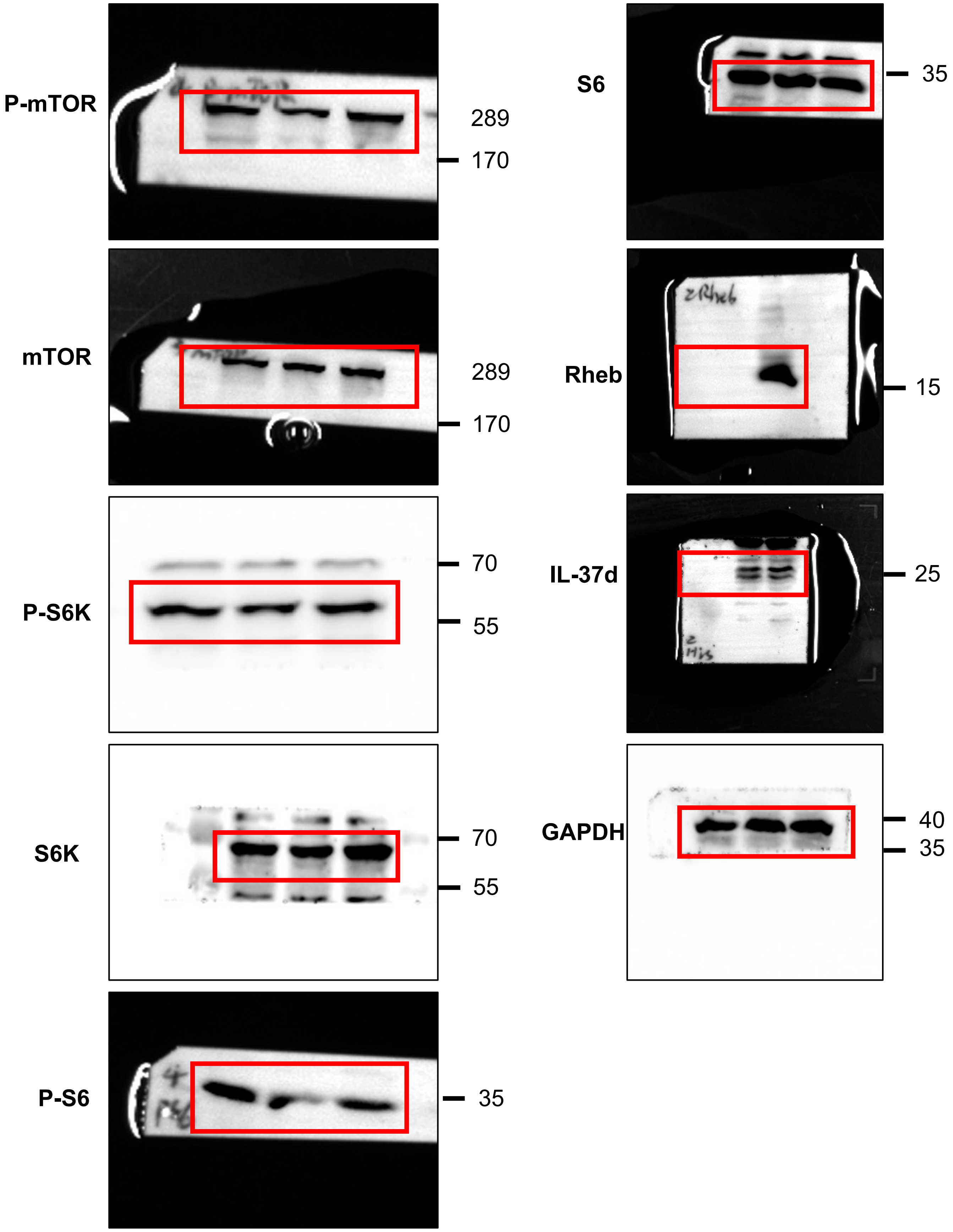

Supplemental Material to Fig 2a (original blots)

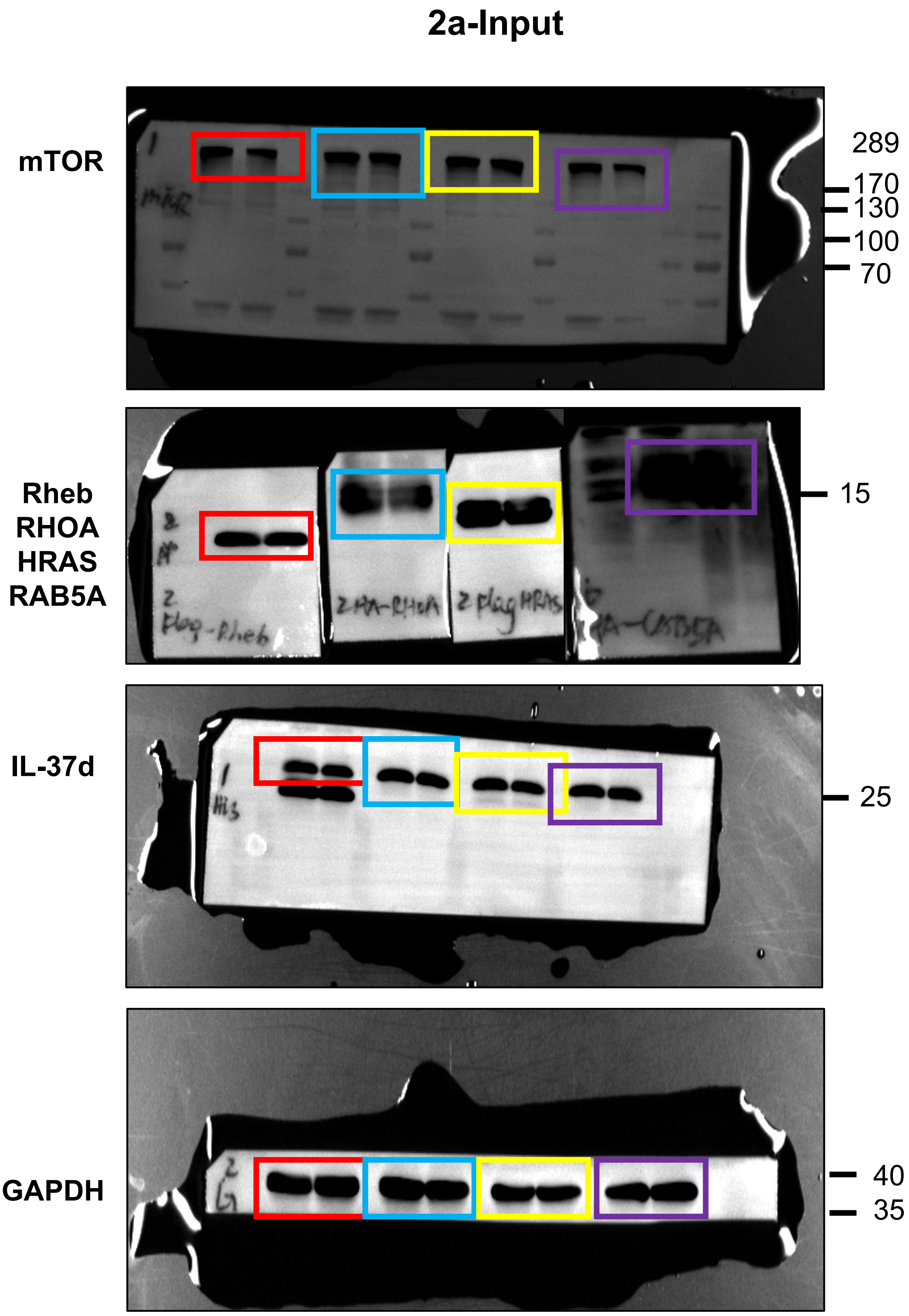

Supplemental Material to Fig 2a (original blots)

2a-IP

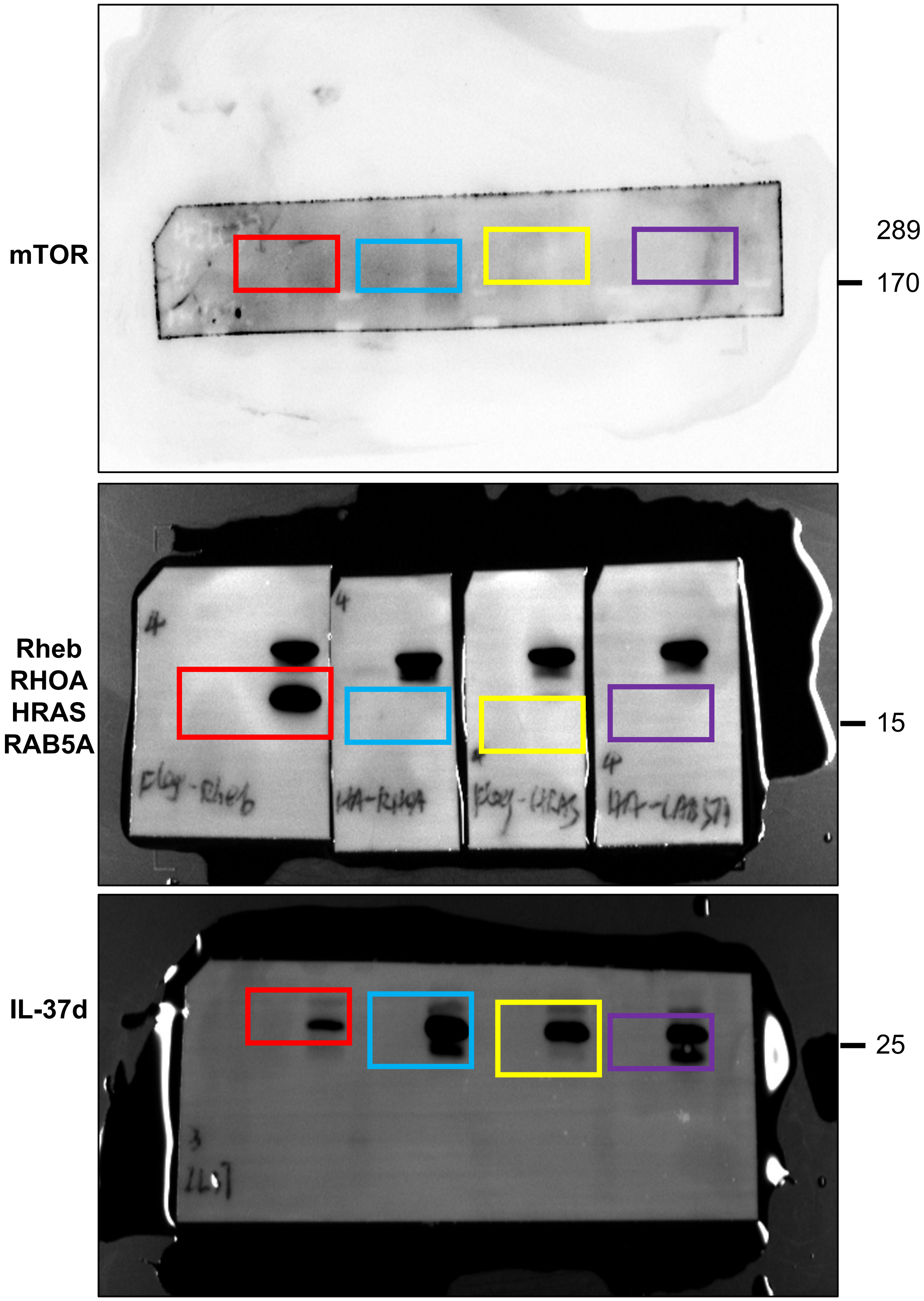

Supplemental Material to Fig 2b, e (original blots)

Fig 2b

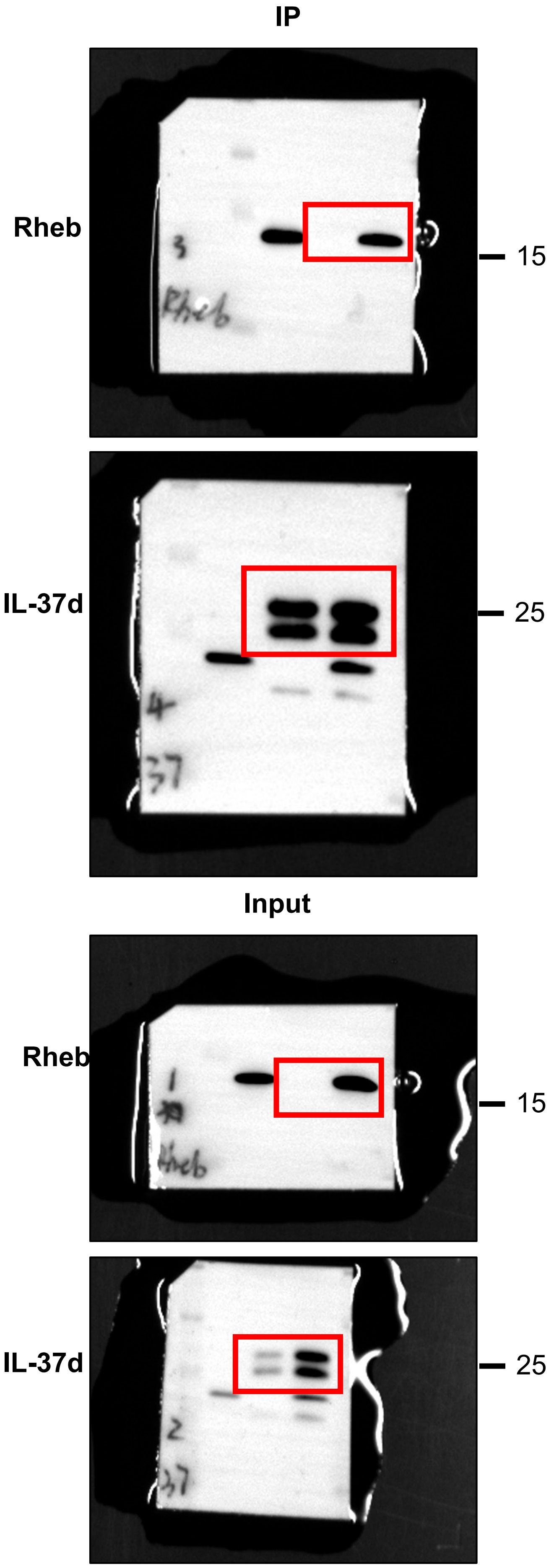

Fig 2e

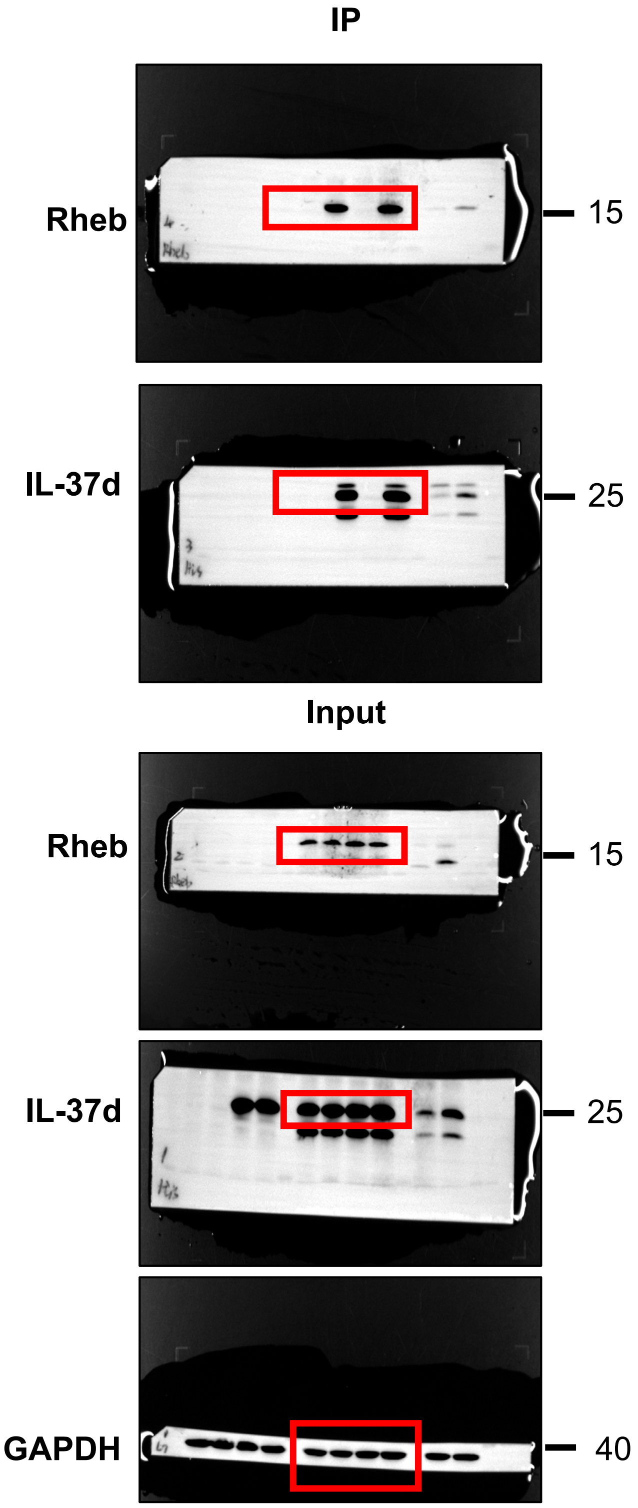

Supplemental Material to Fig 2g-h (original blots)

Fig 2g

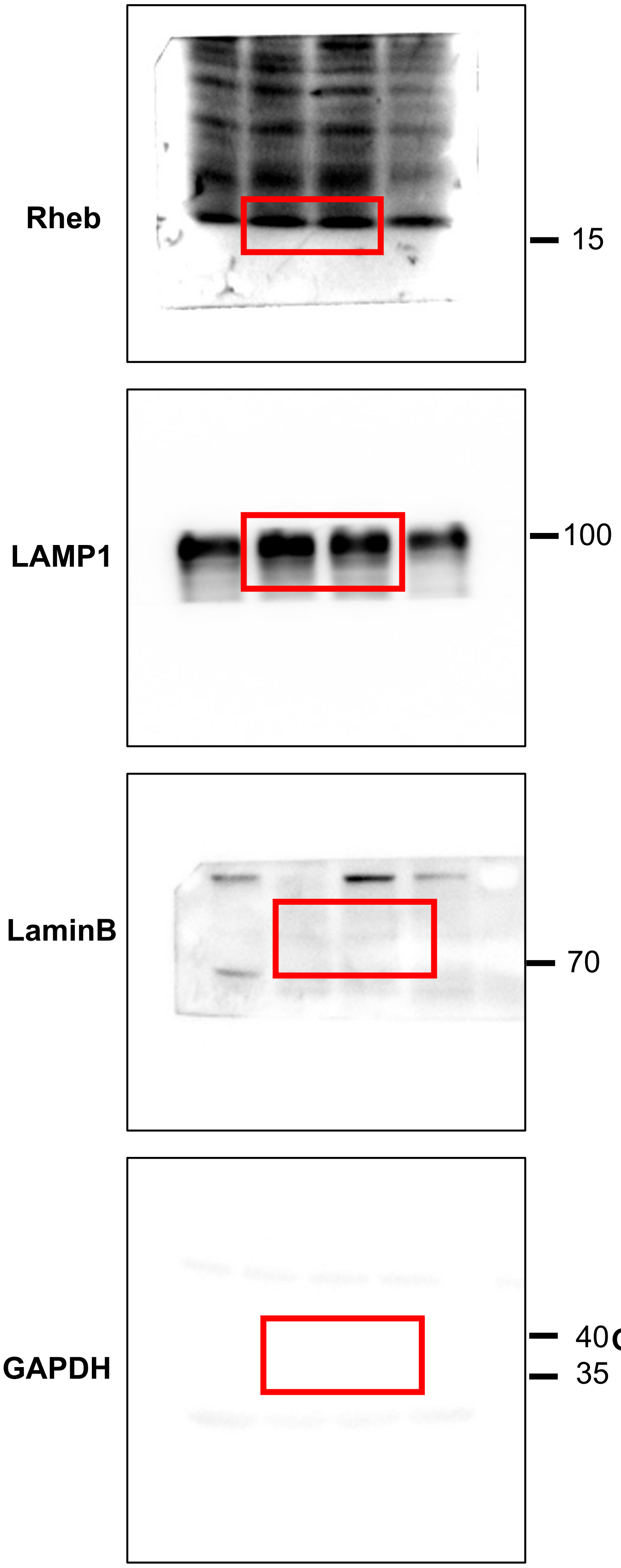

Fig 2h

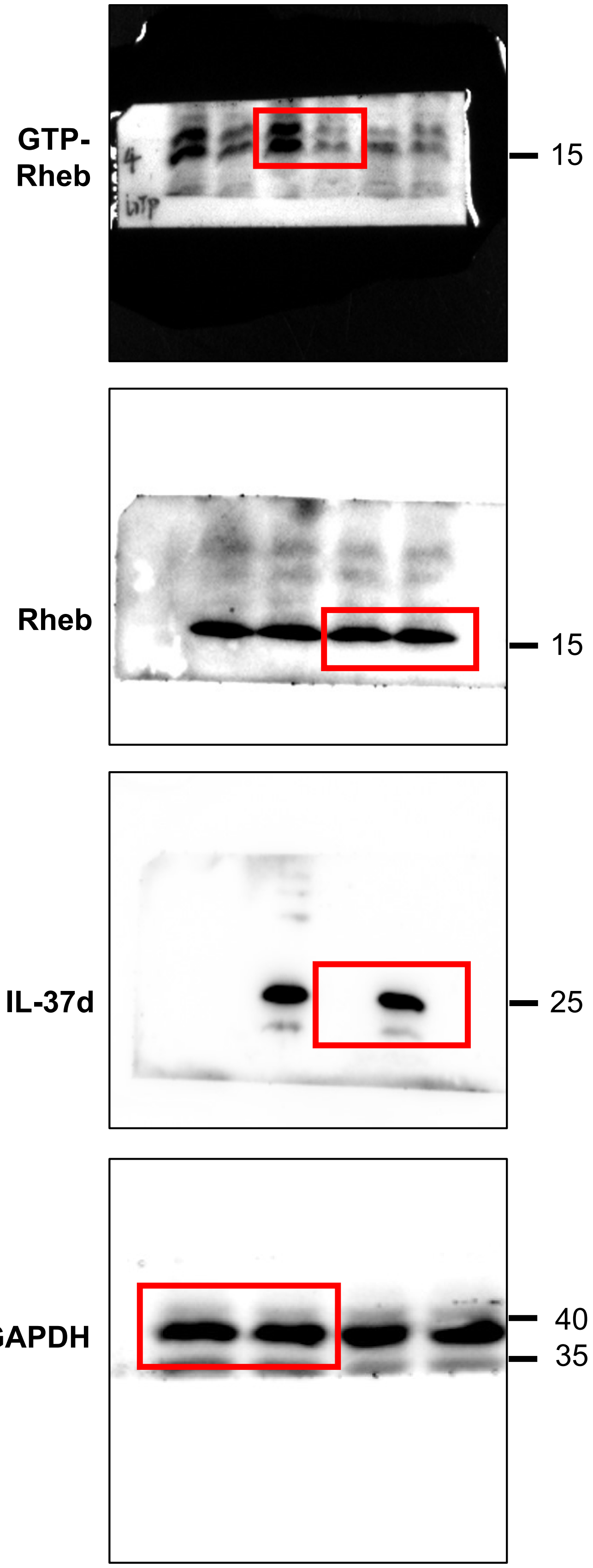

Supplemental Material to Fig 2i, k (original blots)

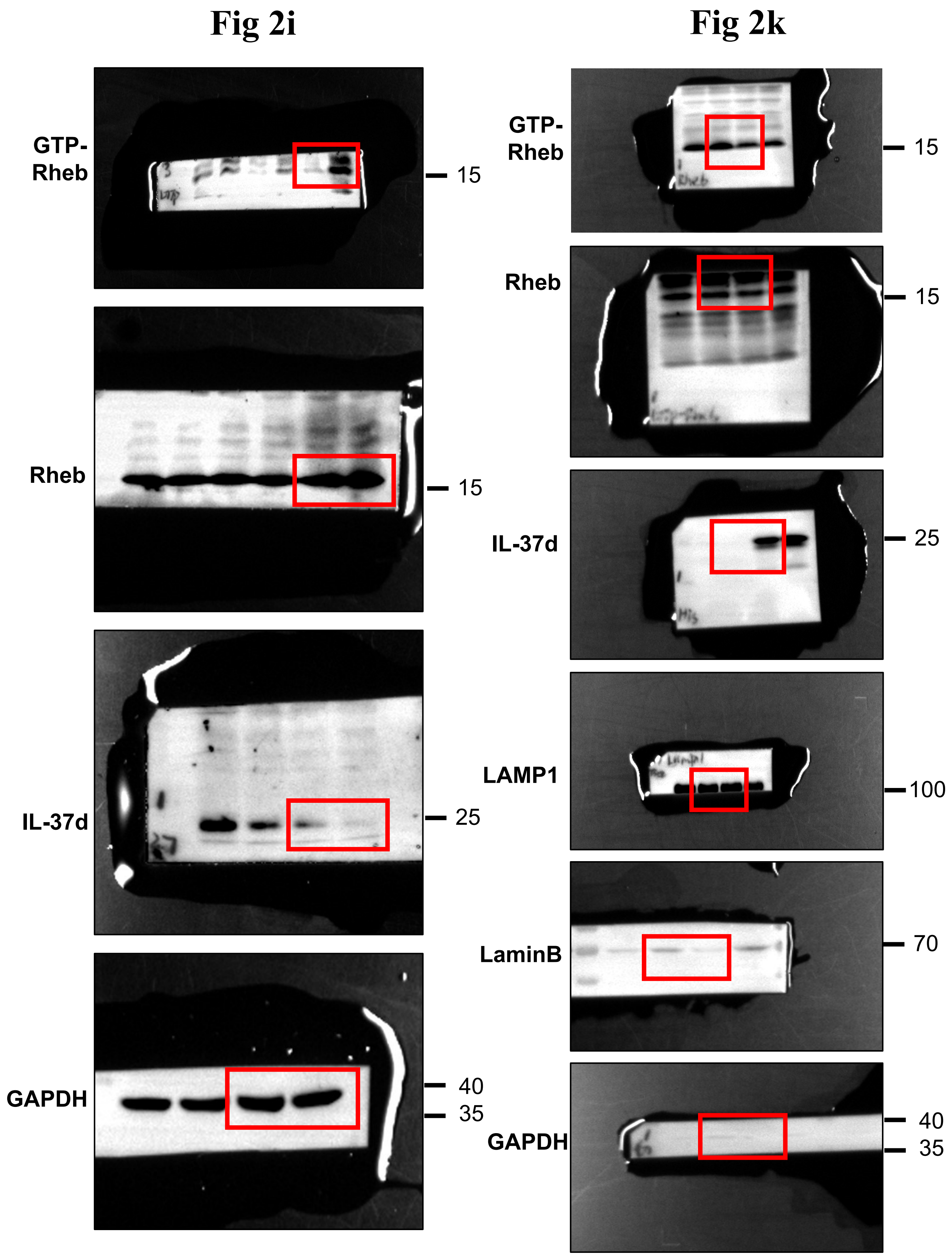

Supplemental Material to Fig 2l (original blots)

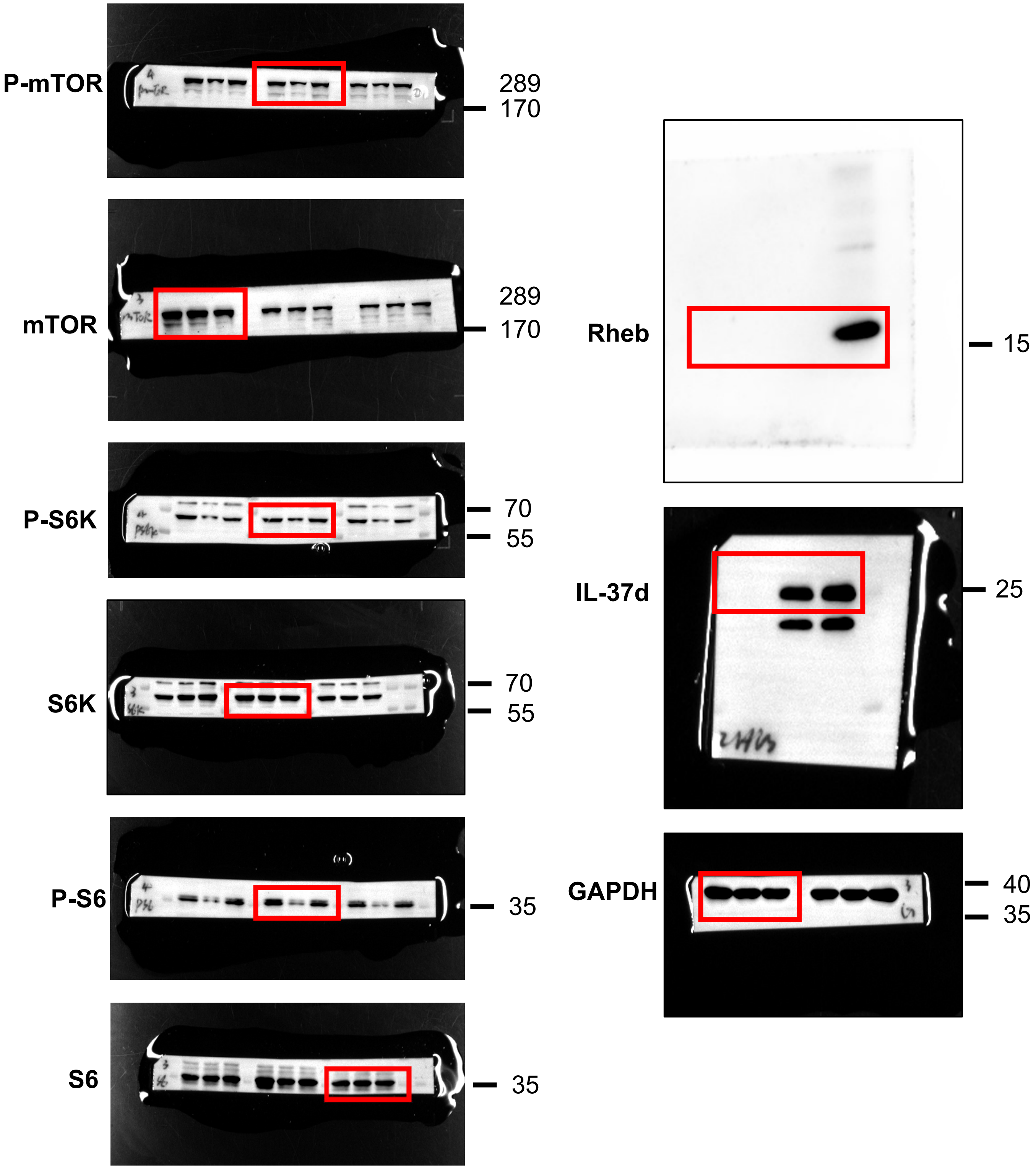

Supplemental Material to Fig 3a (original blots)

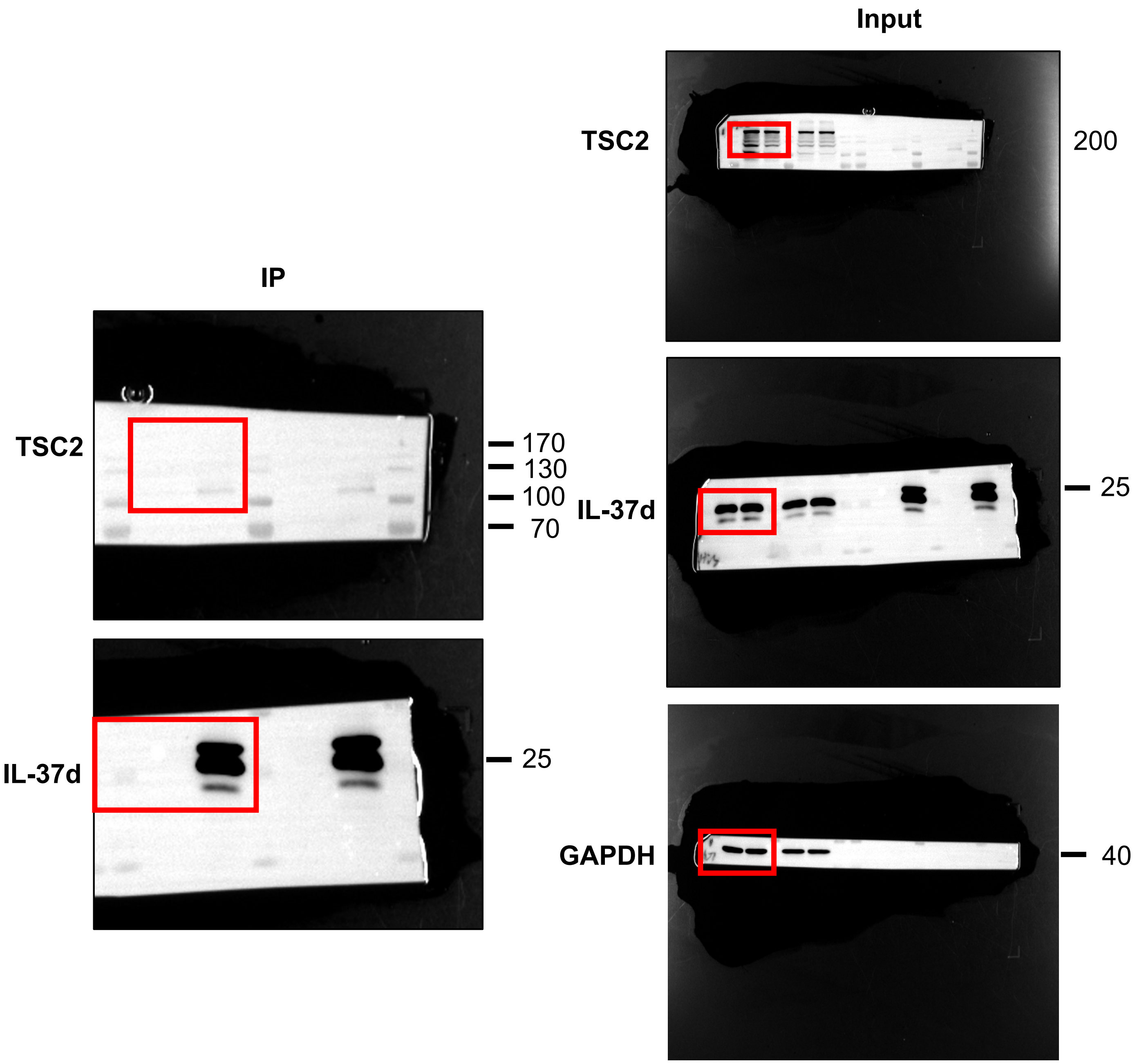

Supplemental Material to Fig 3b (original blots)

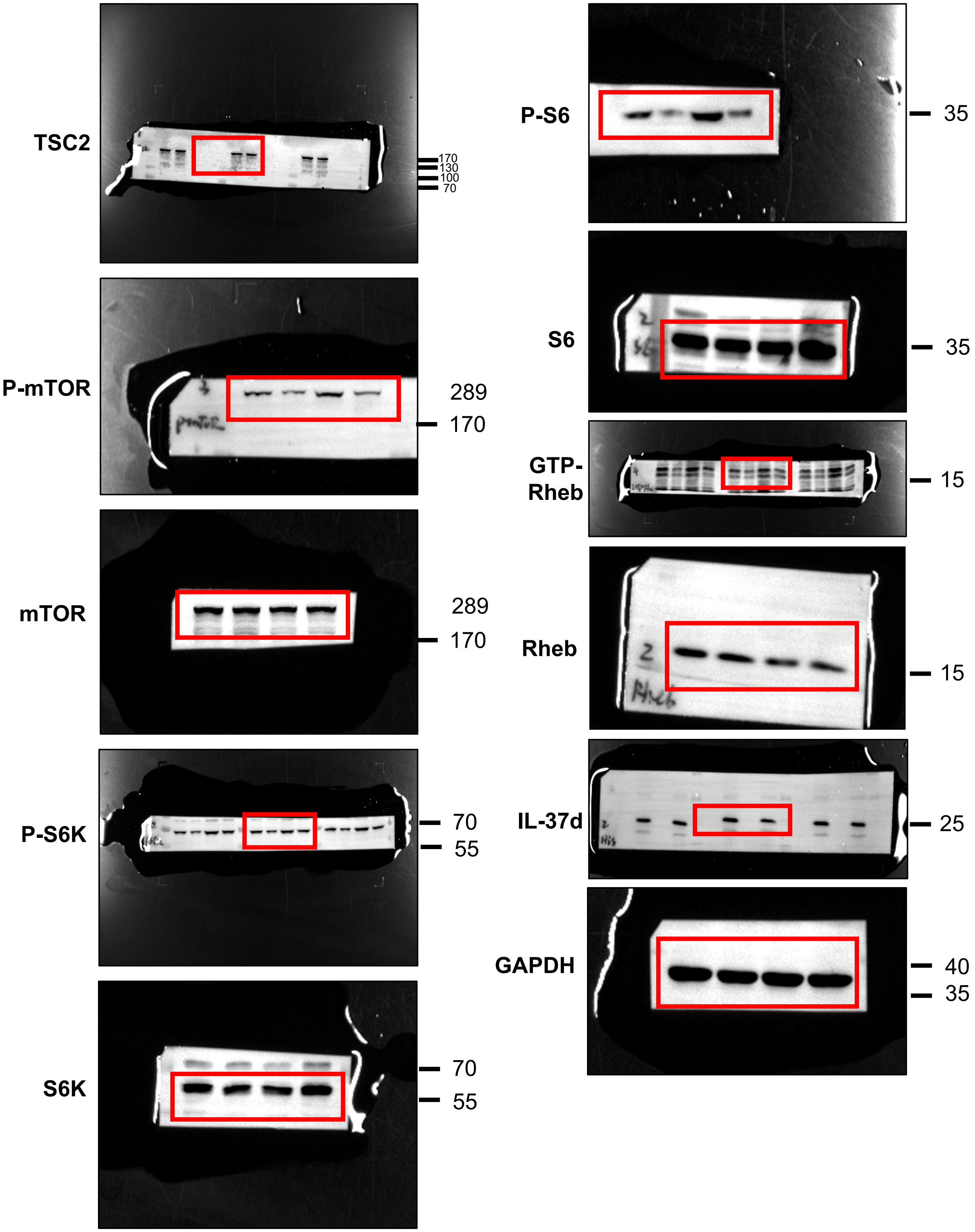

Supplemental Material to Fig 3c (original blots)

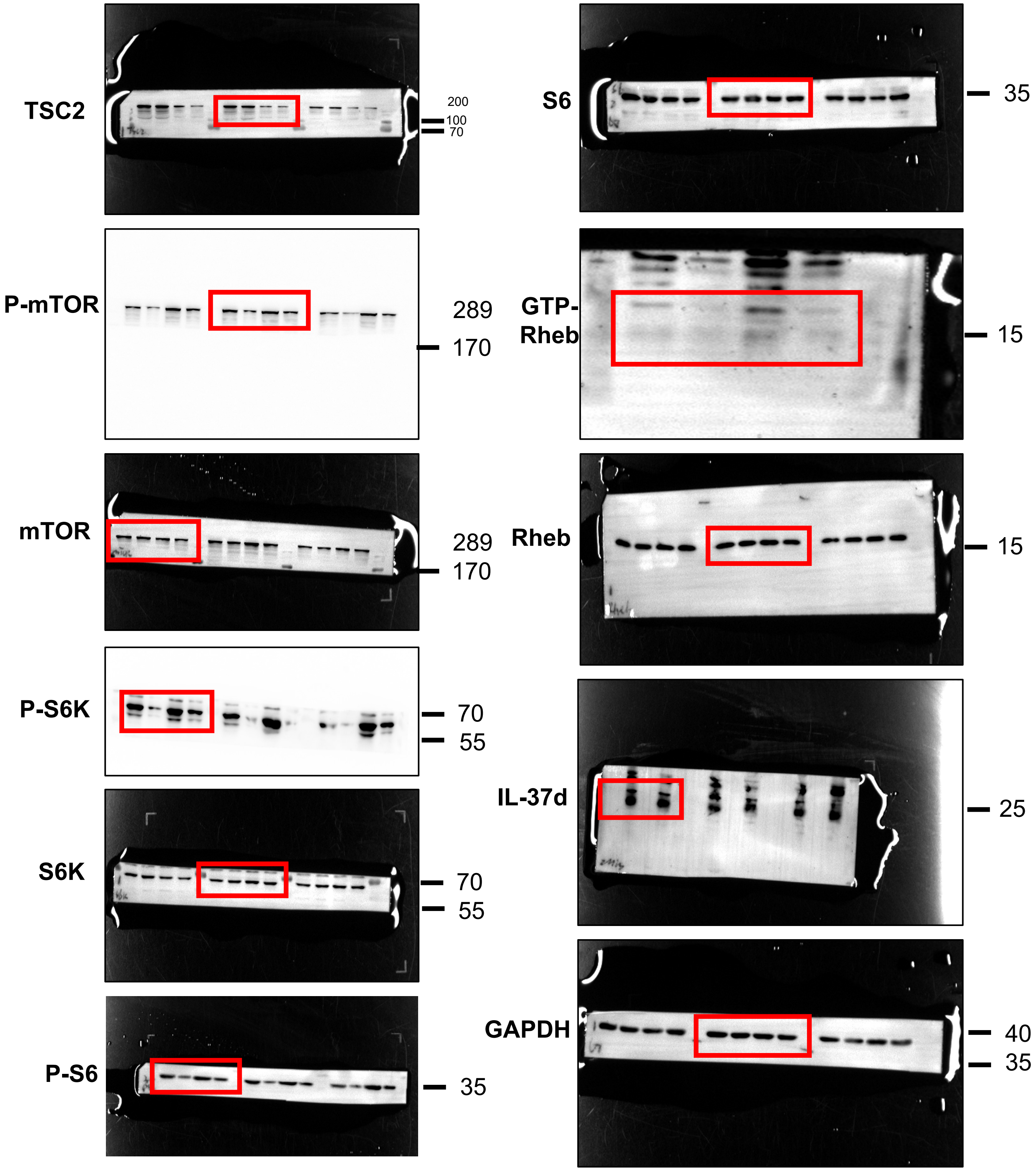

Supplemental Material to Fig 3d-e (original blots)

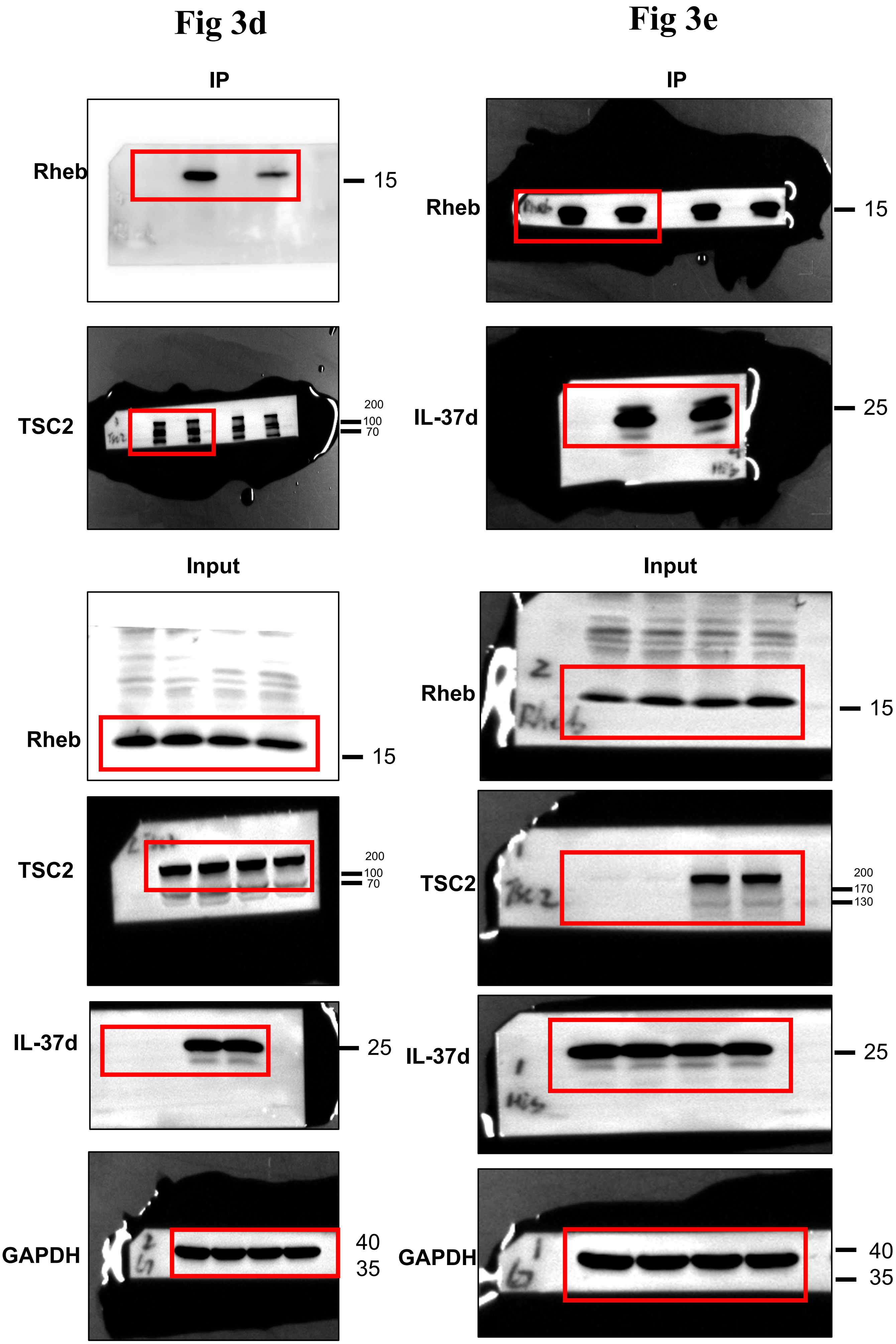

Supplemental Material to Fig 3f (original blots)

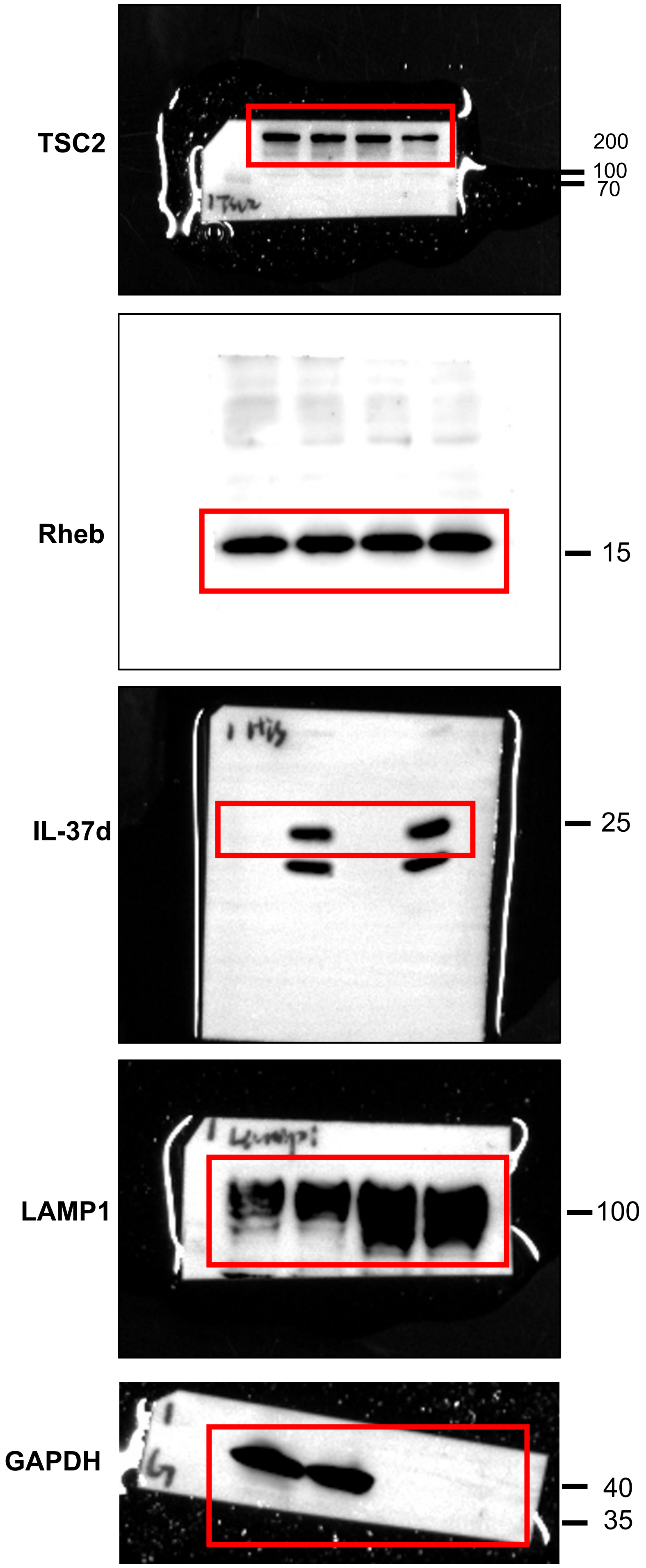

Supplemental Material to Fig 4c (original blots)

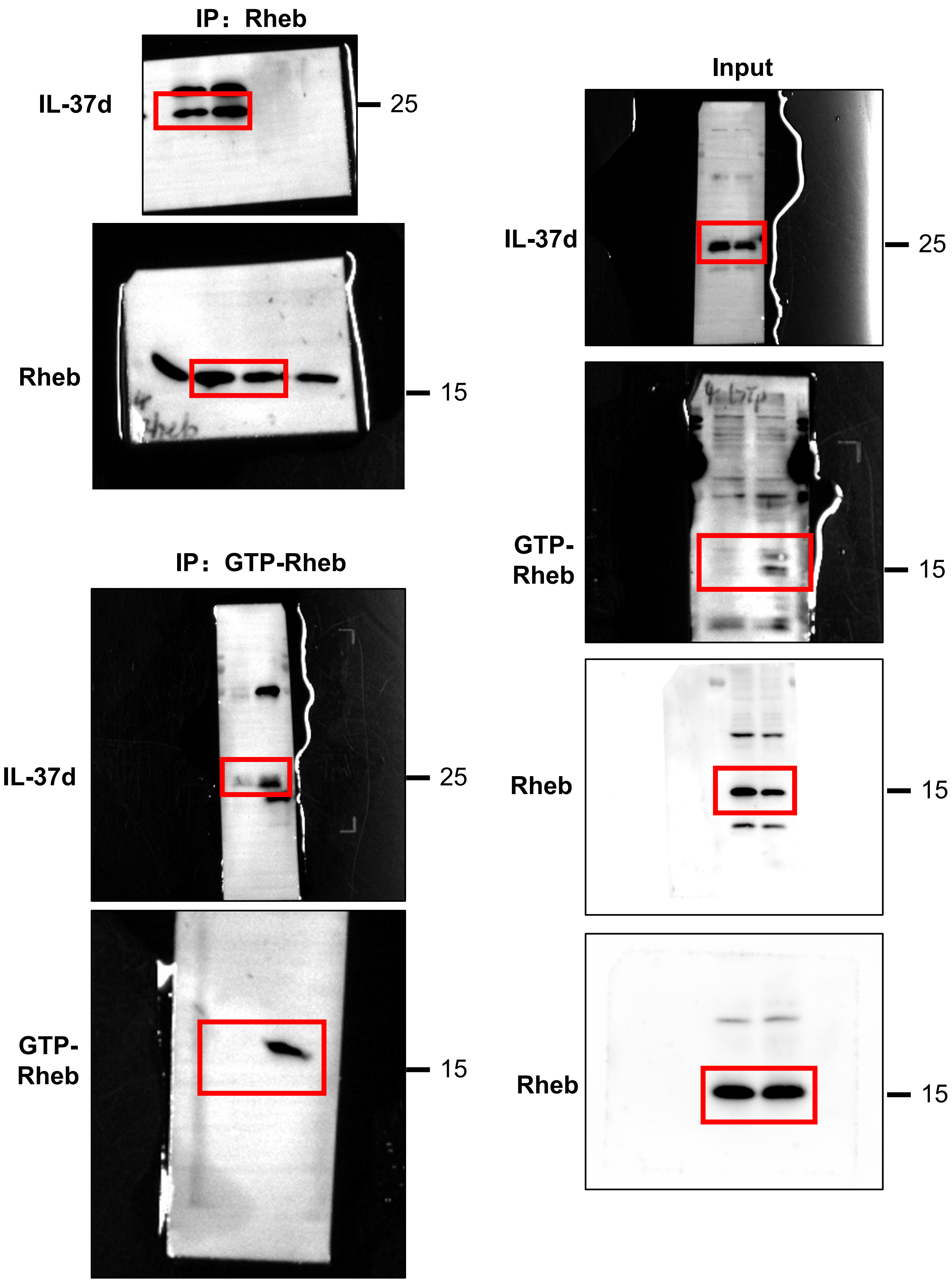

Supplemental Material to Fig 4d-e (original blots)

Fig 4d Fig 4e

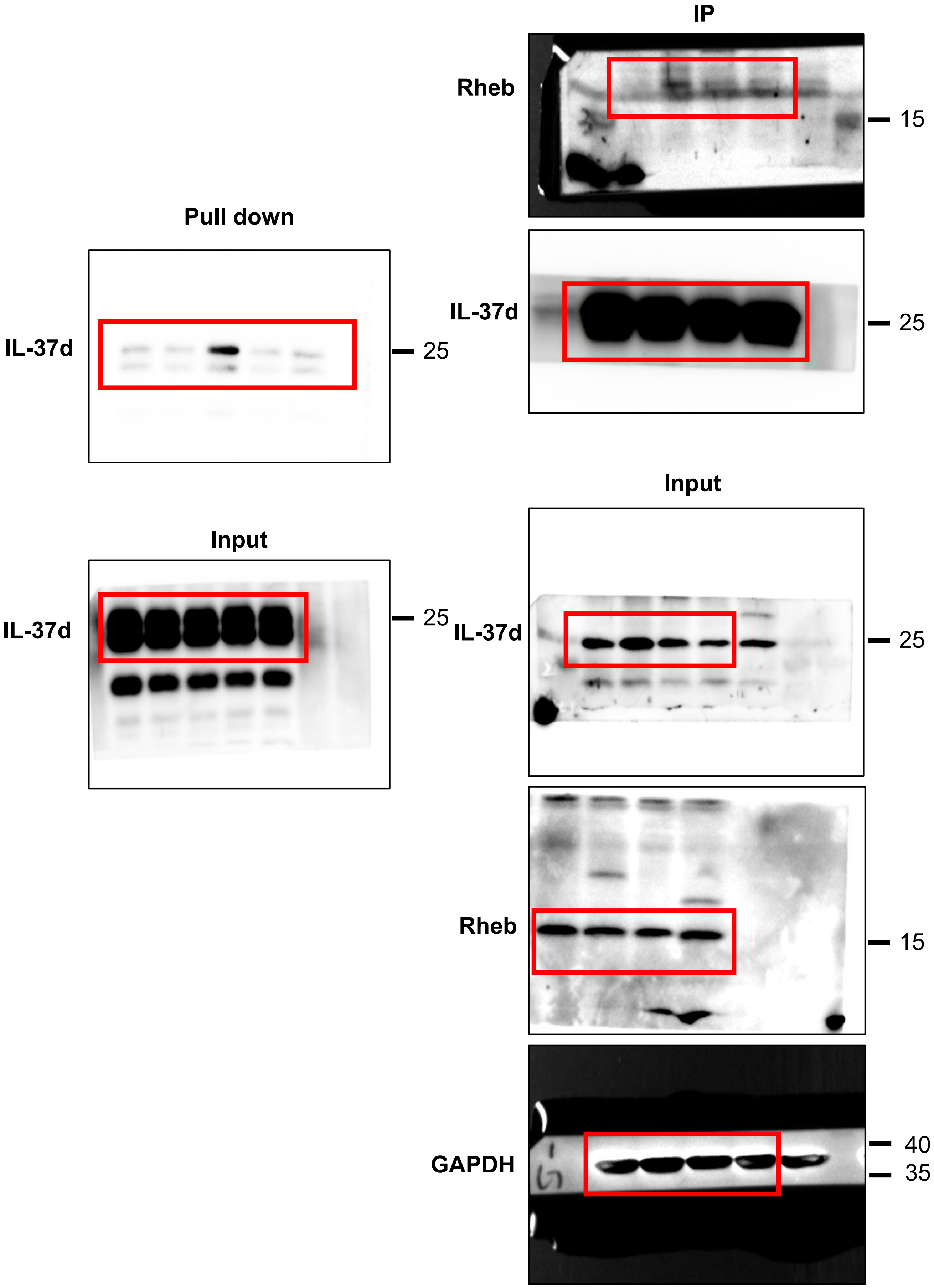

Supplemental Material to Fig 4f, h (original blots)

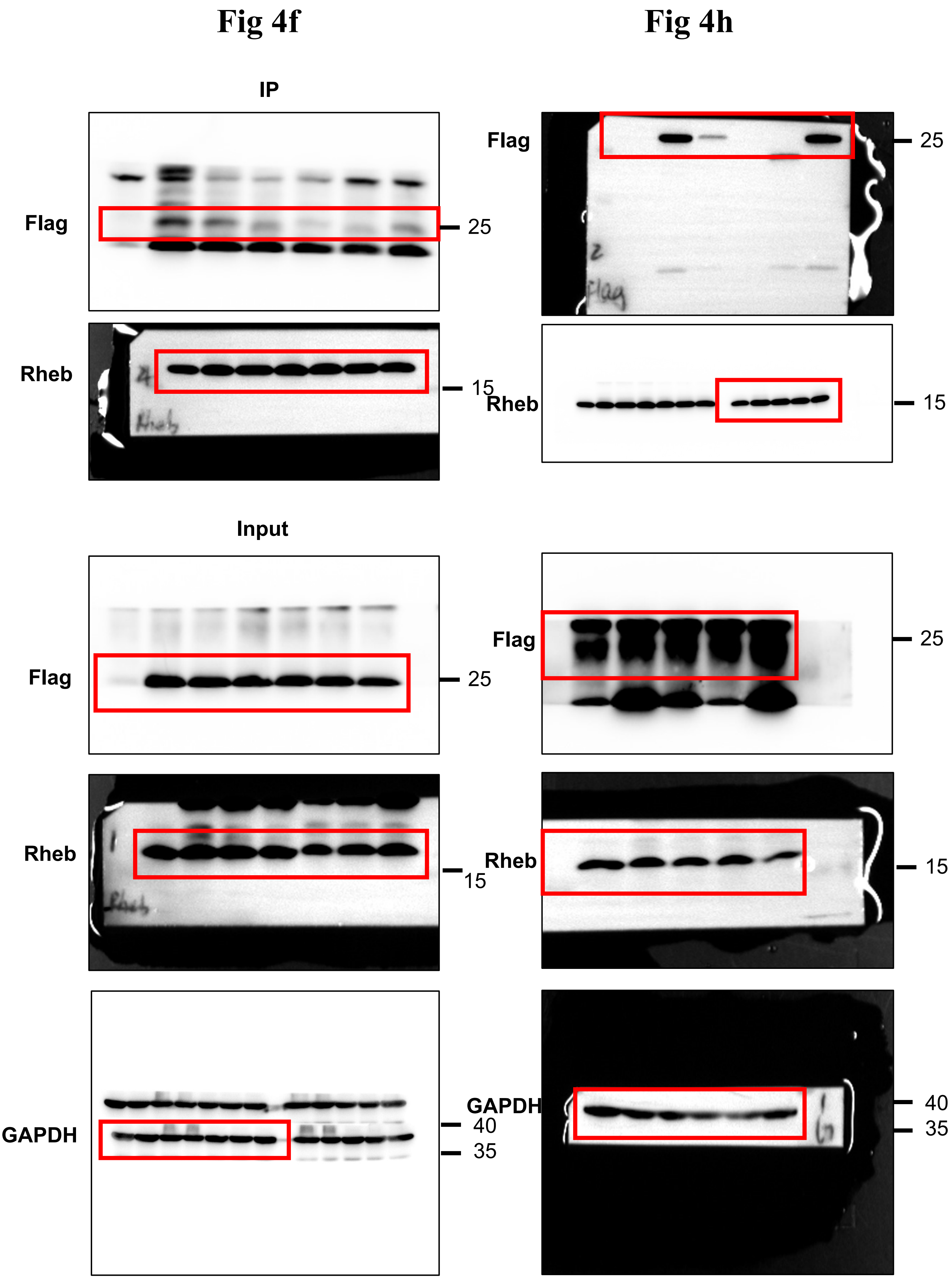

Supplemental Material to Fig 4j-k (original blots)

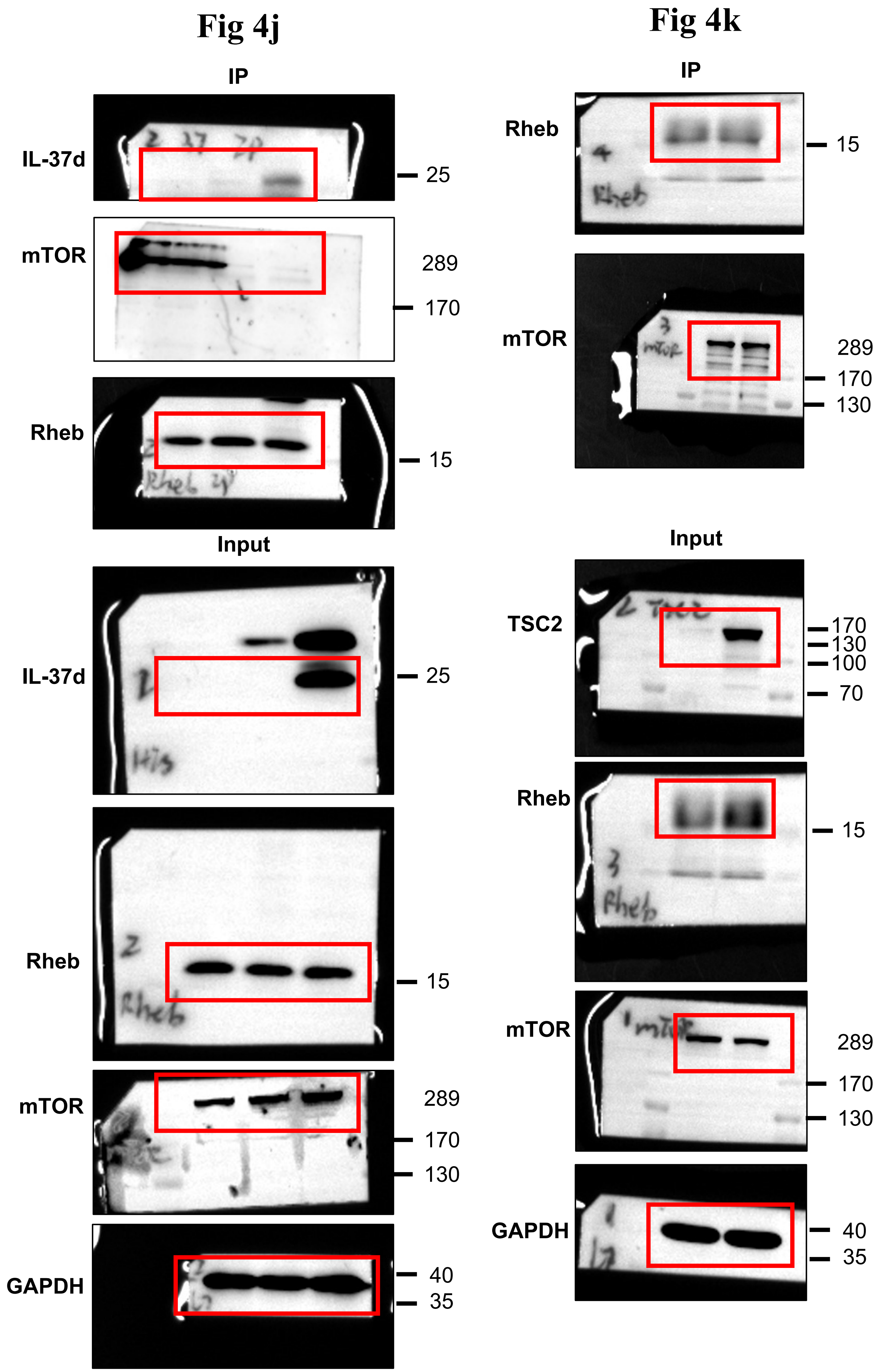

Supplemental Material to Fig 4l (original blots)

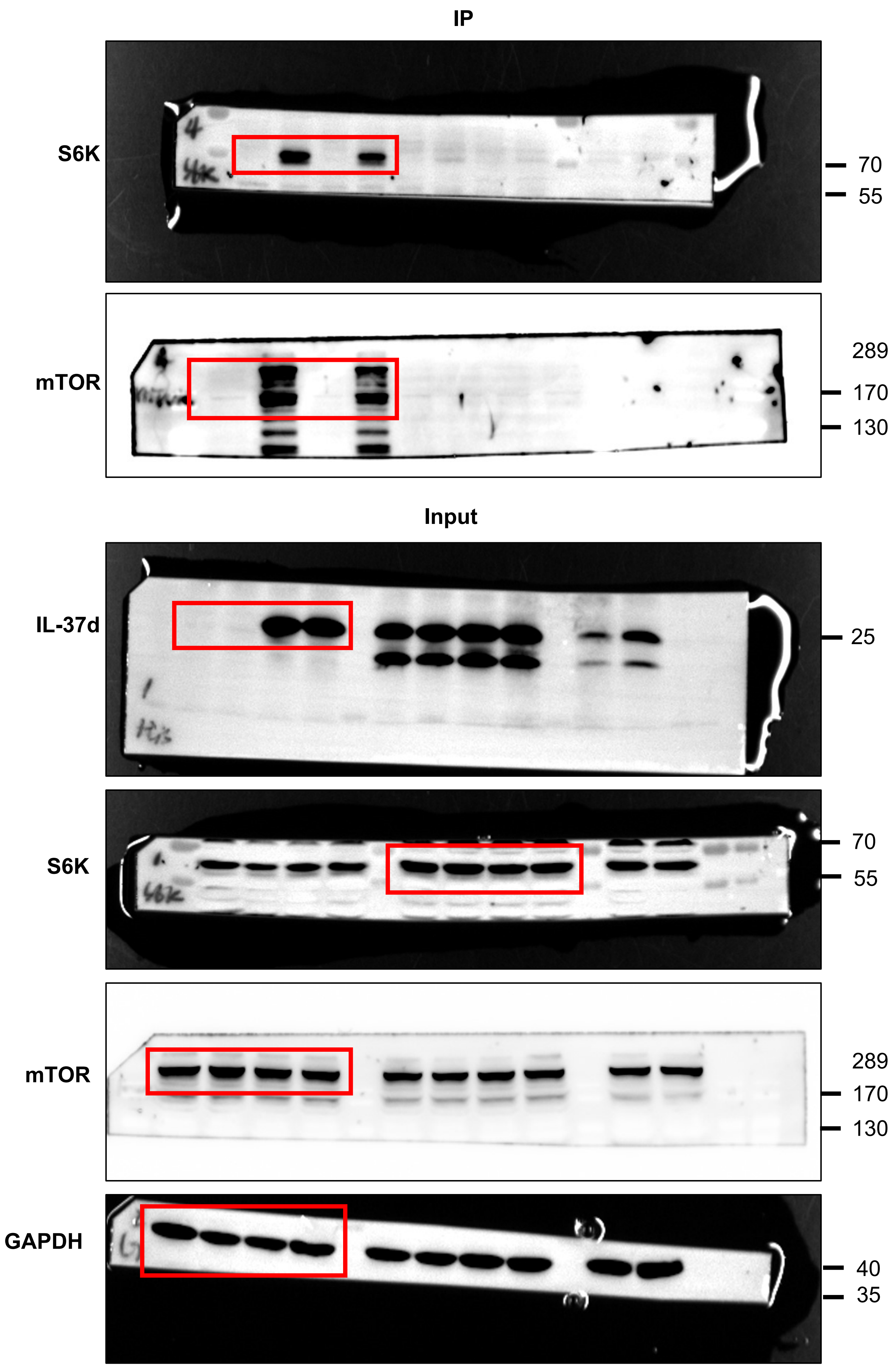

Supplemental Material to Fig 4m (original blots)

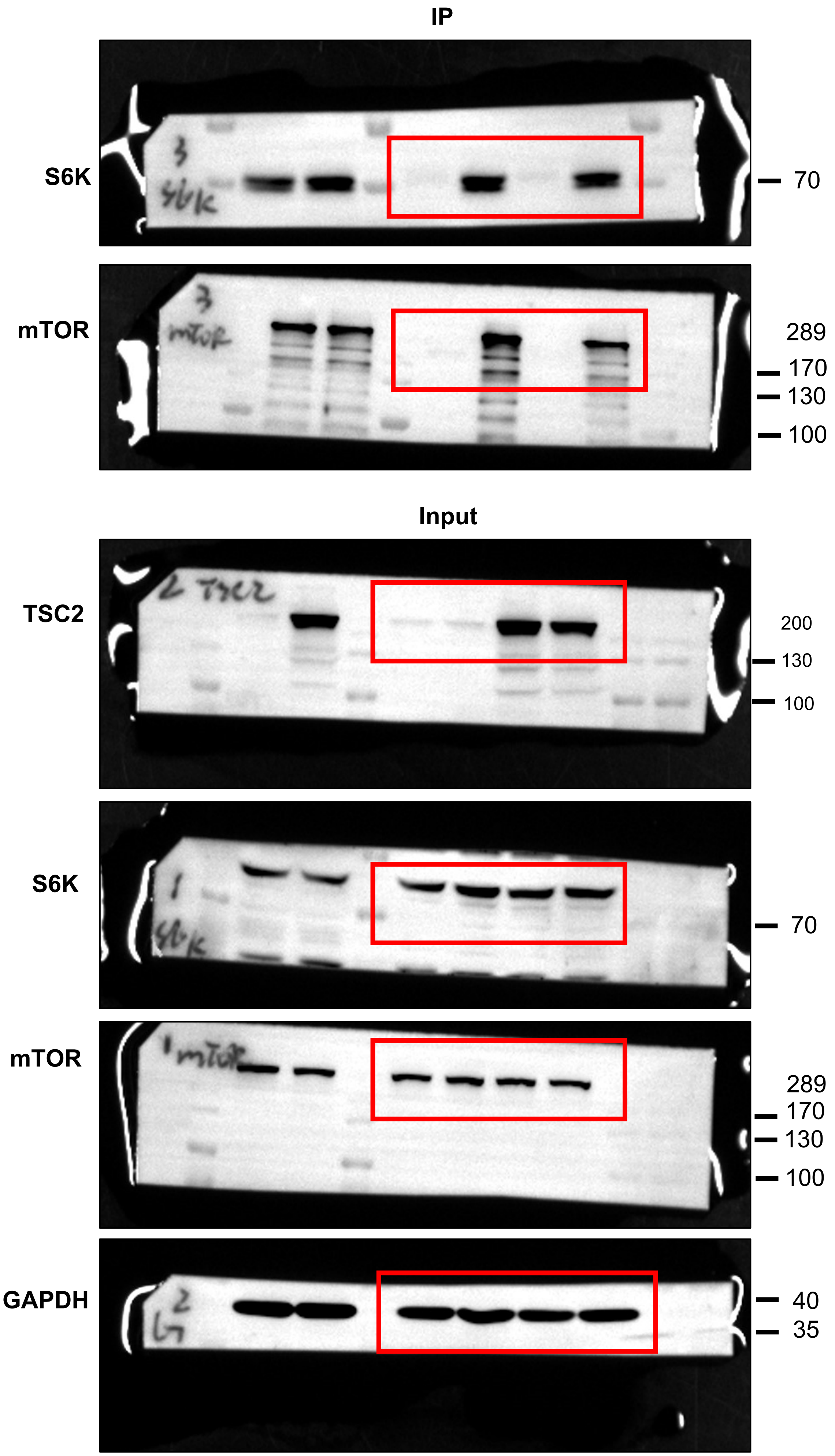

Supplemental Material to Fig 5a, d (original blots)

Fig 5a

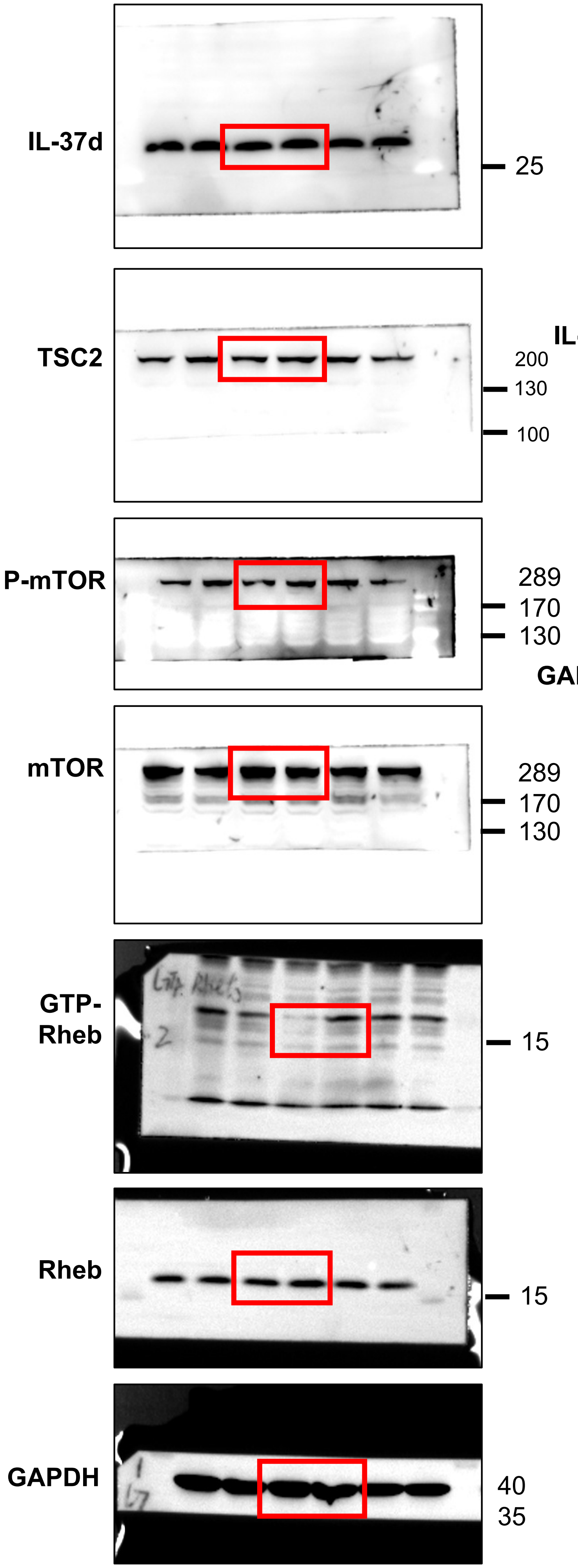

Fig 5d

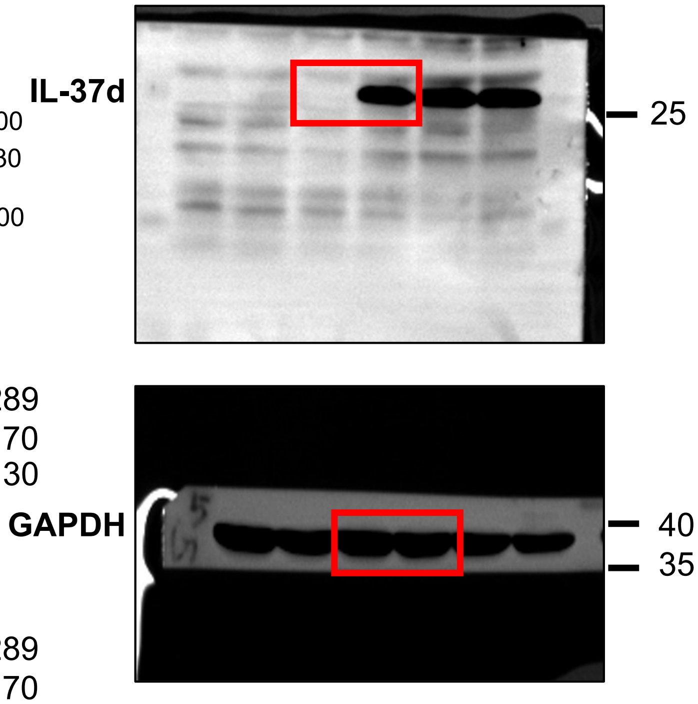

Supplemental Material to Fig 5b (original blots)

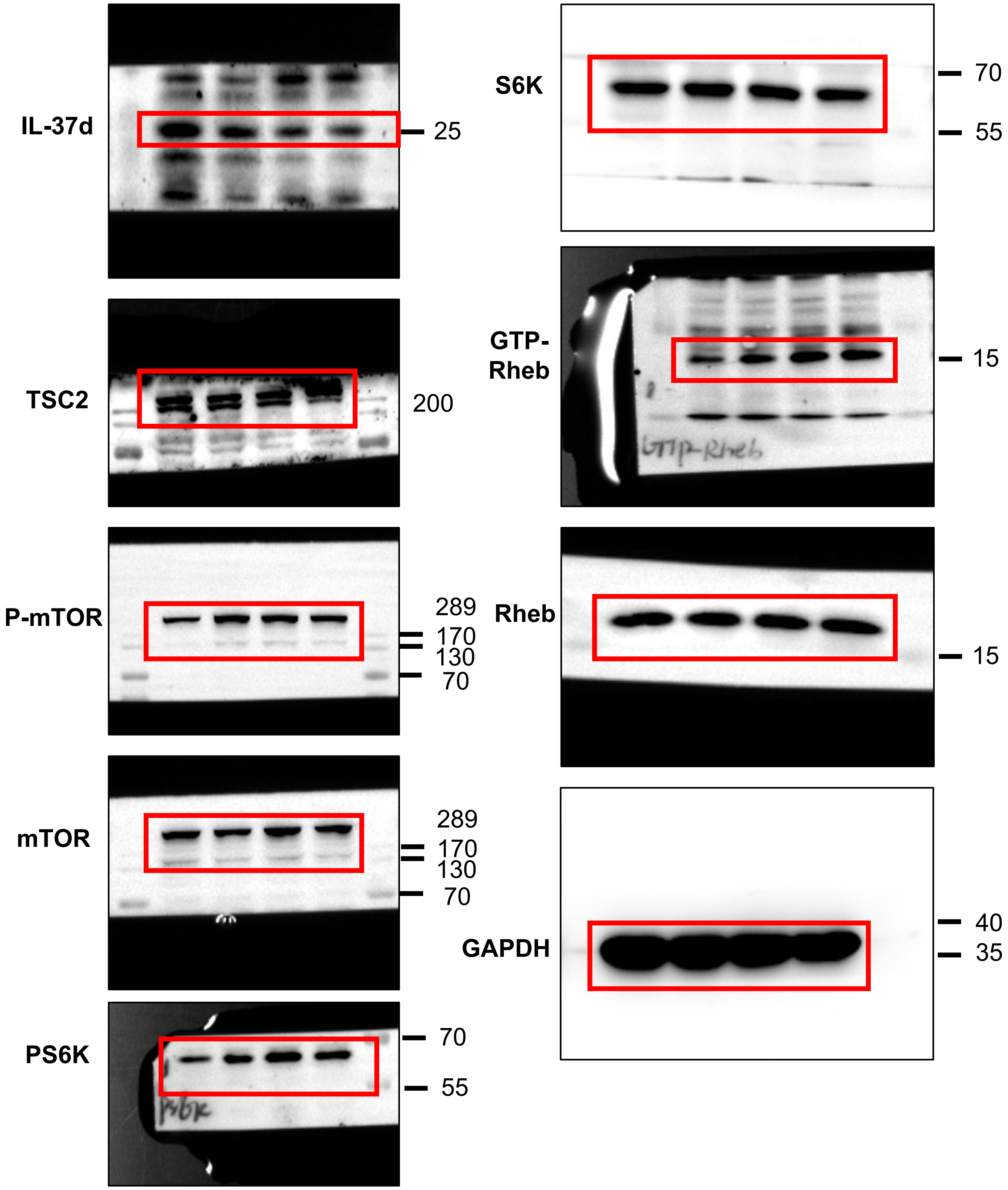

Supplemental Material to Fig 5e (original blots)

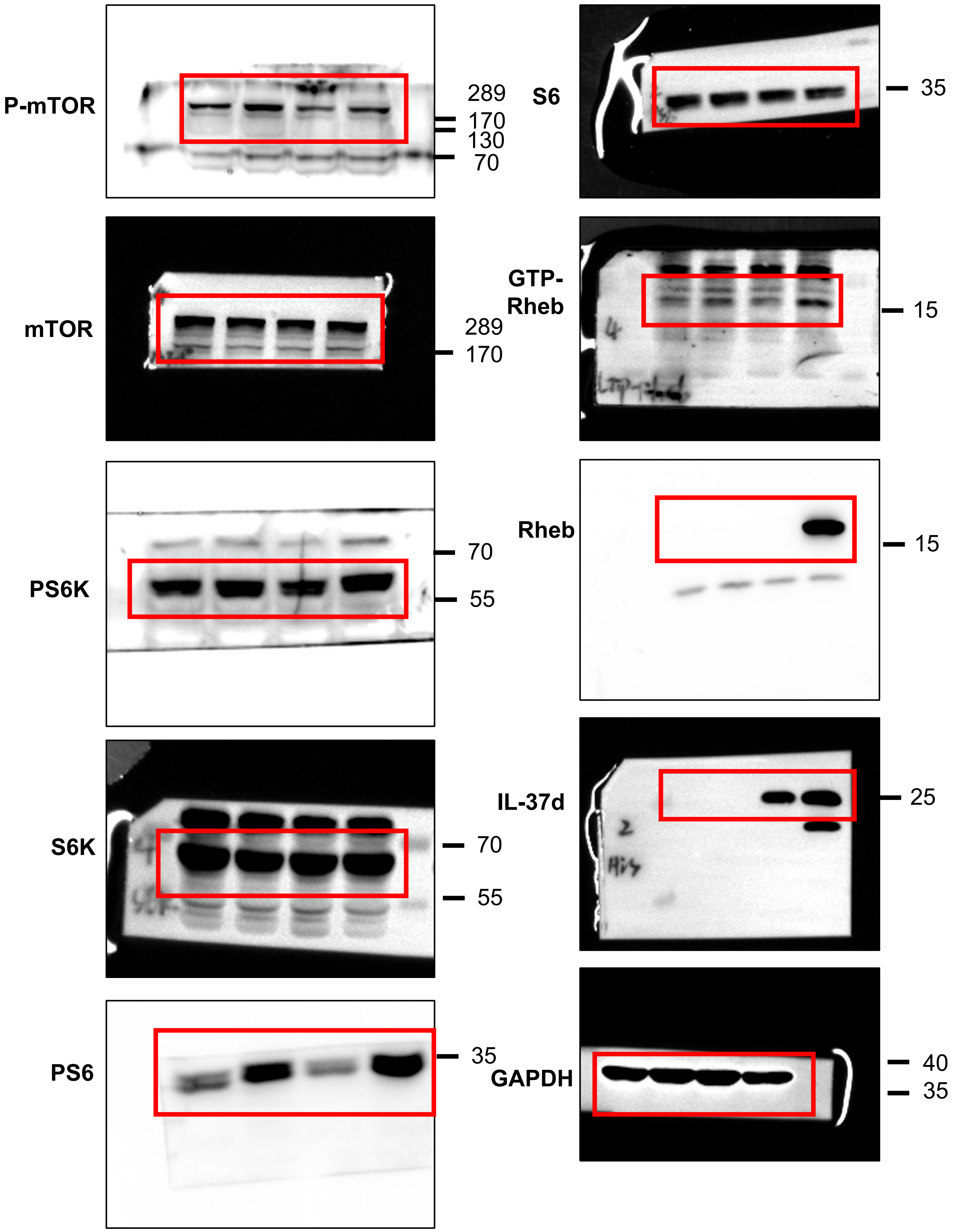

Supplemental Material to Fig 6k (original blots)

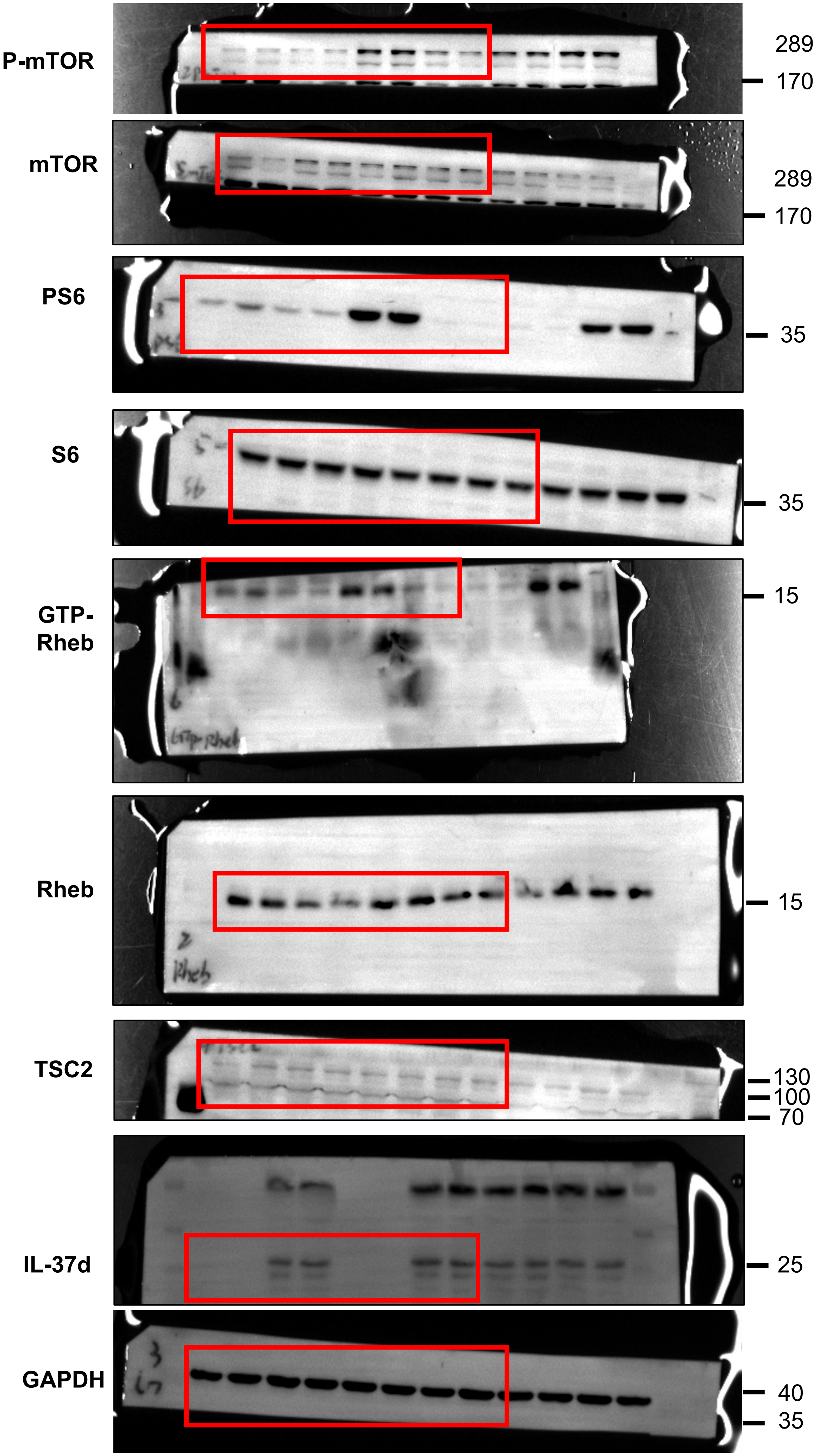

Supplemental Material to Fig 6l (original blots)

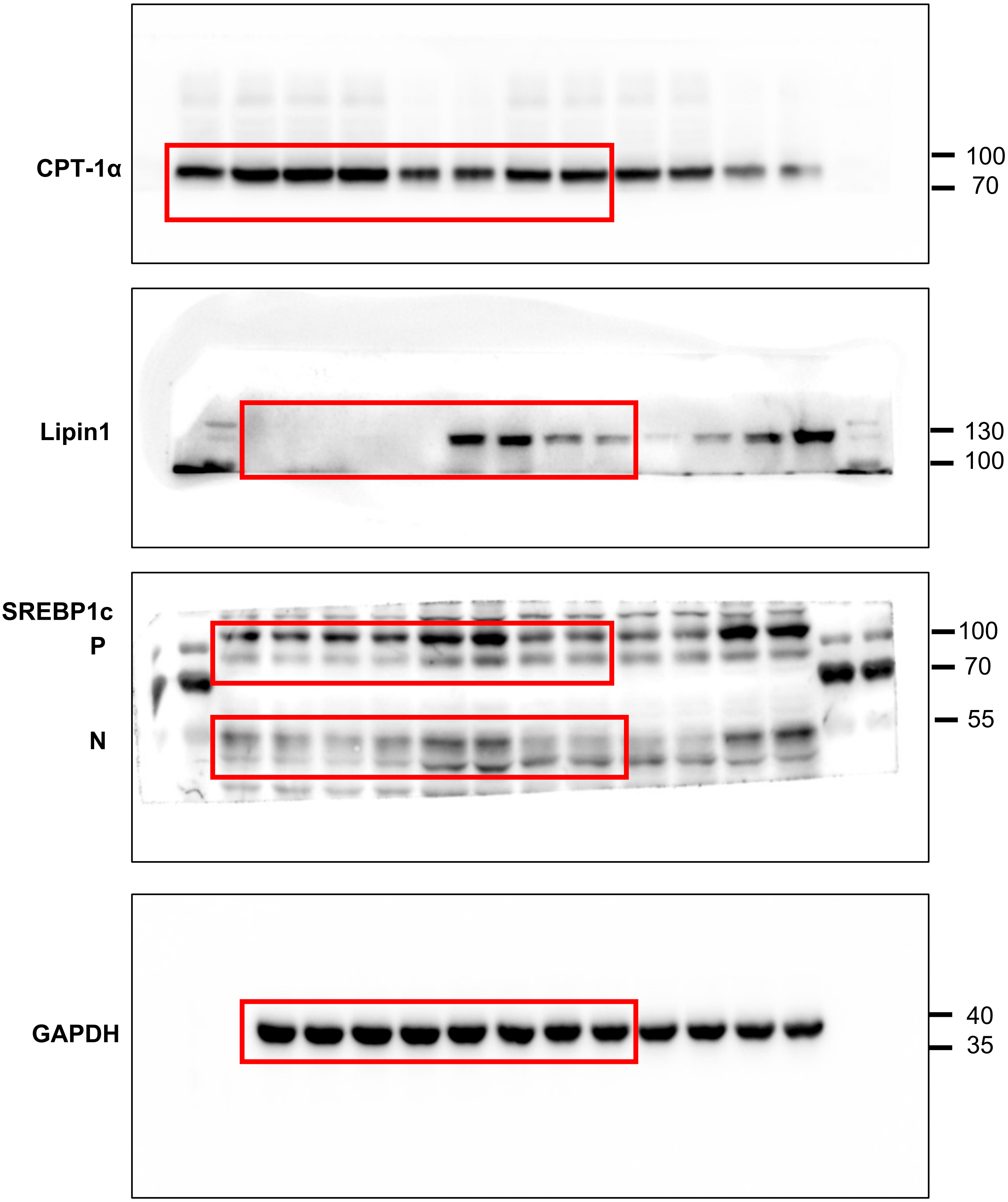

Supplemental Material to Fig 7k (original blots)

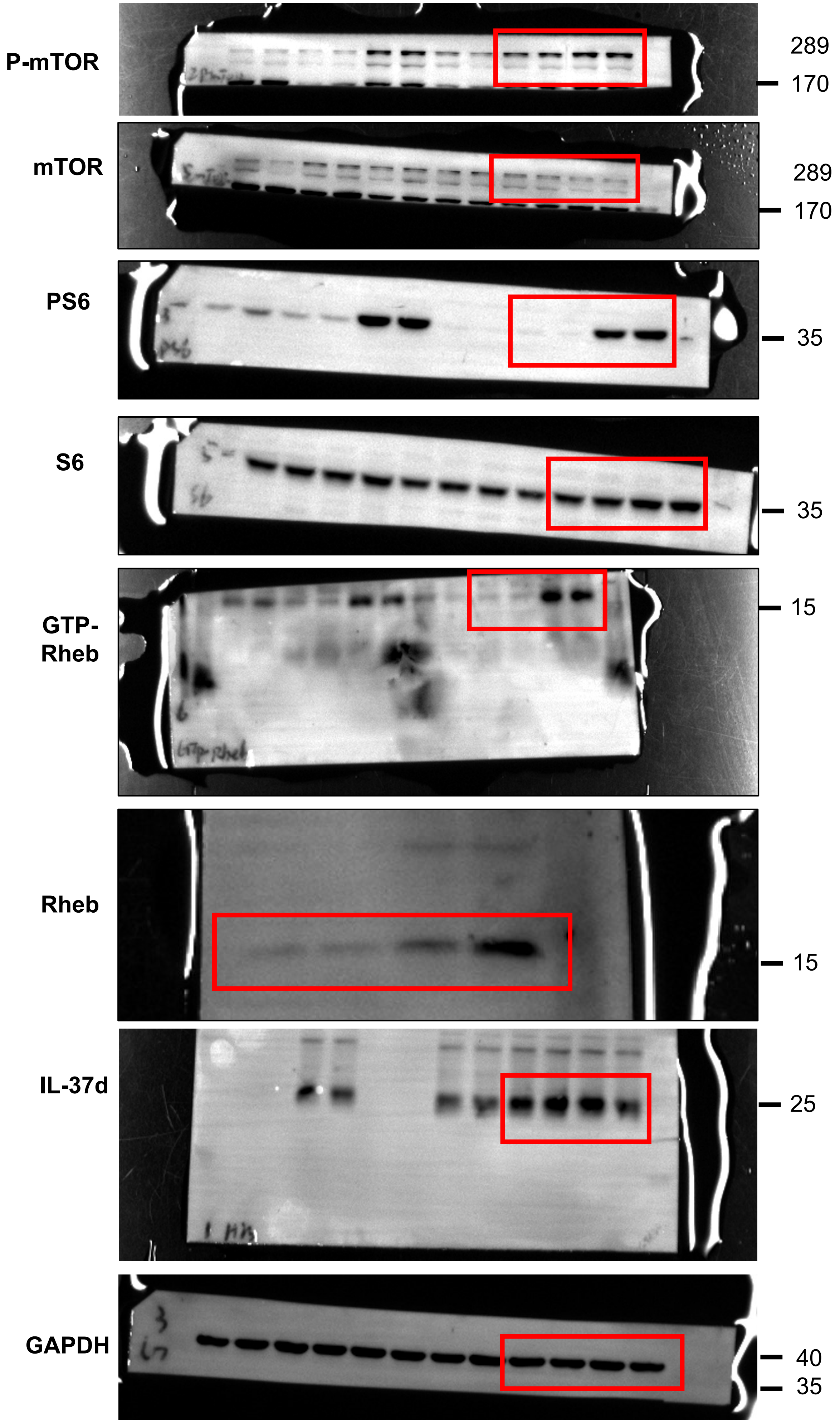

Supplemental Material to Fig 7l(original blots)

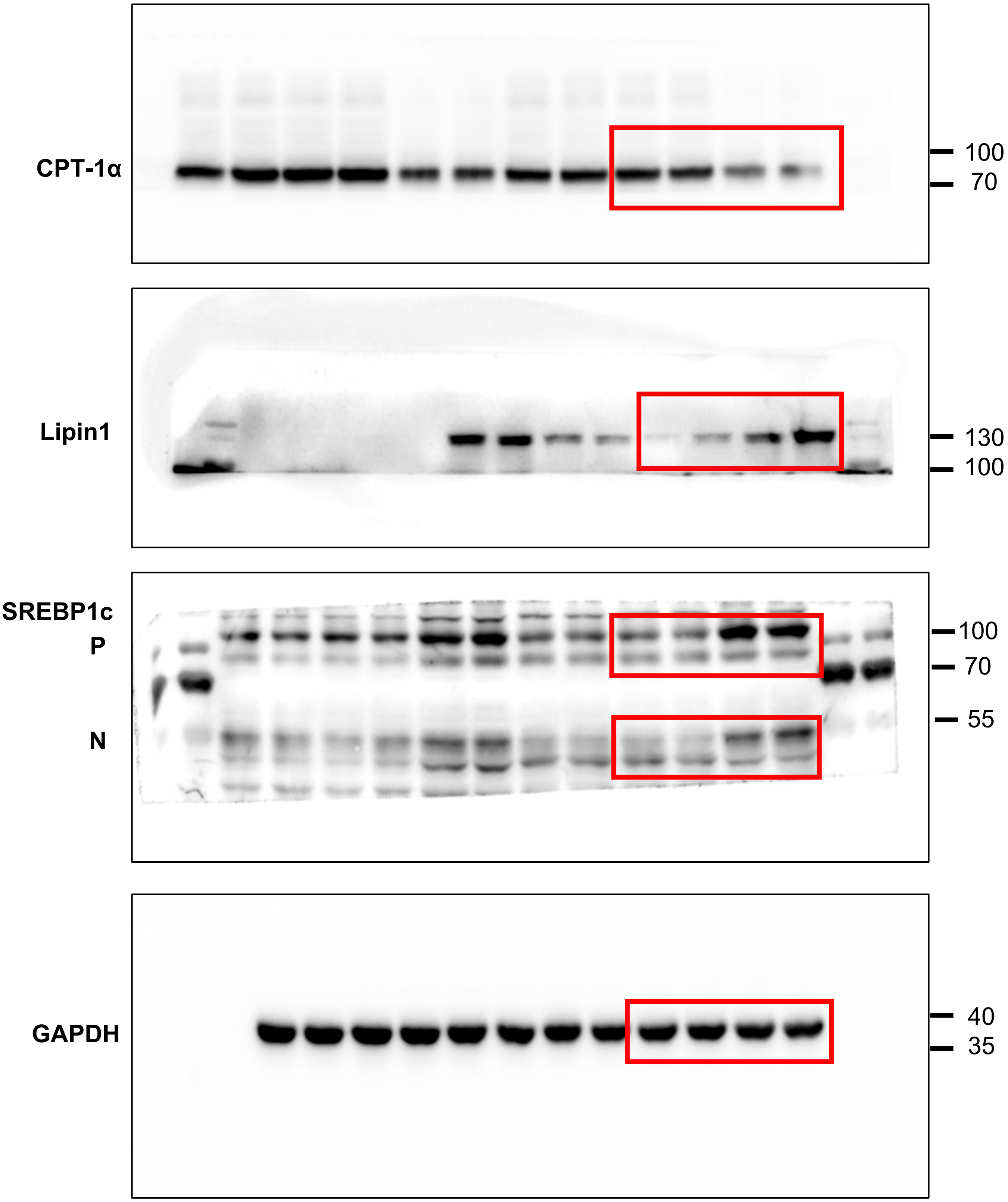

Supplemental Material to Fig 8k (original blots)

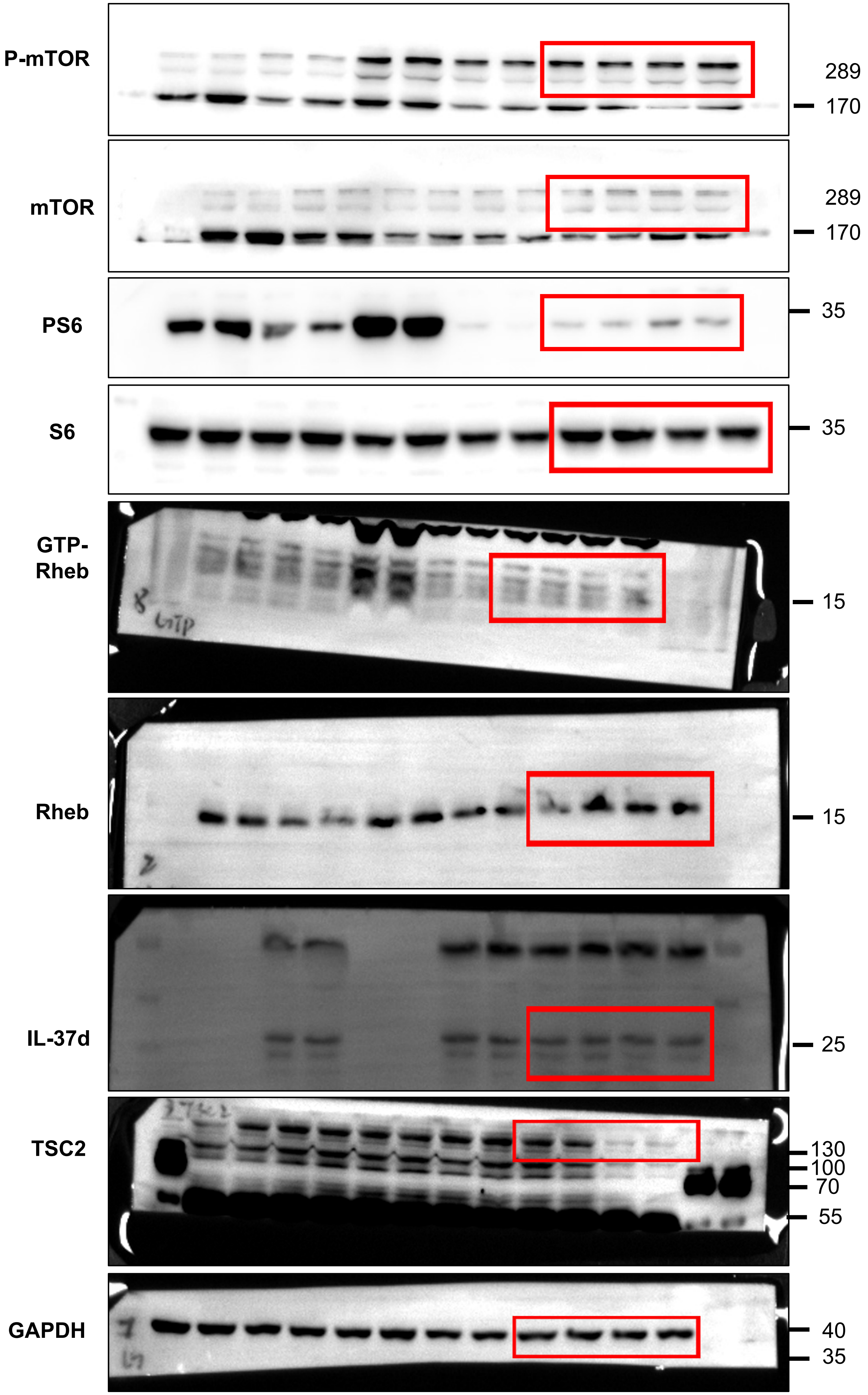

Supplemental Material to Fig 8l (original blots)

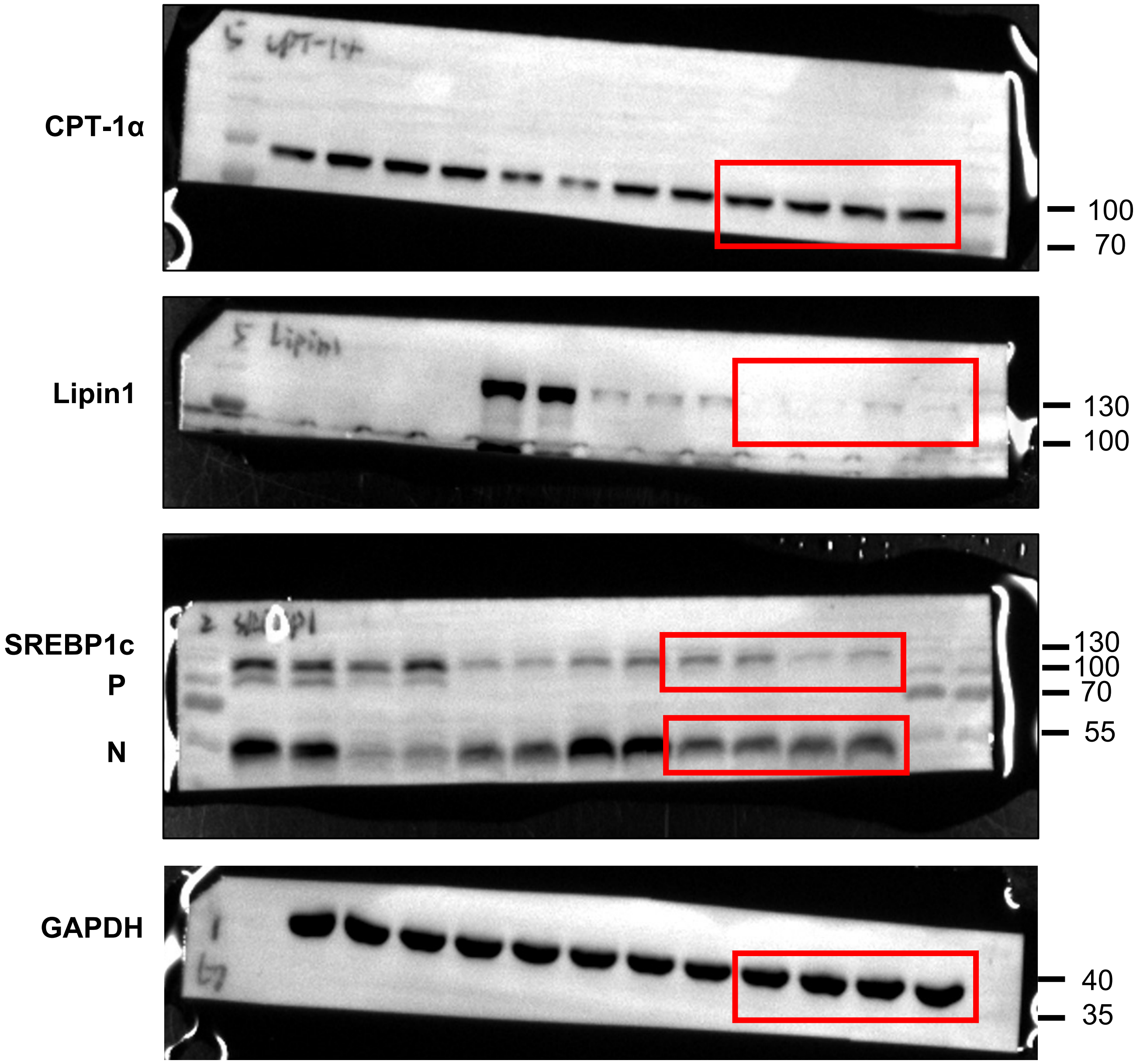

Supplemental Material to Fig S1a (original blots)

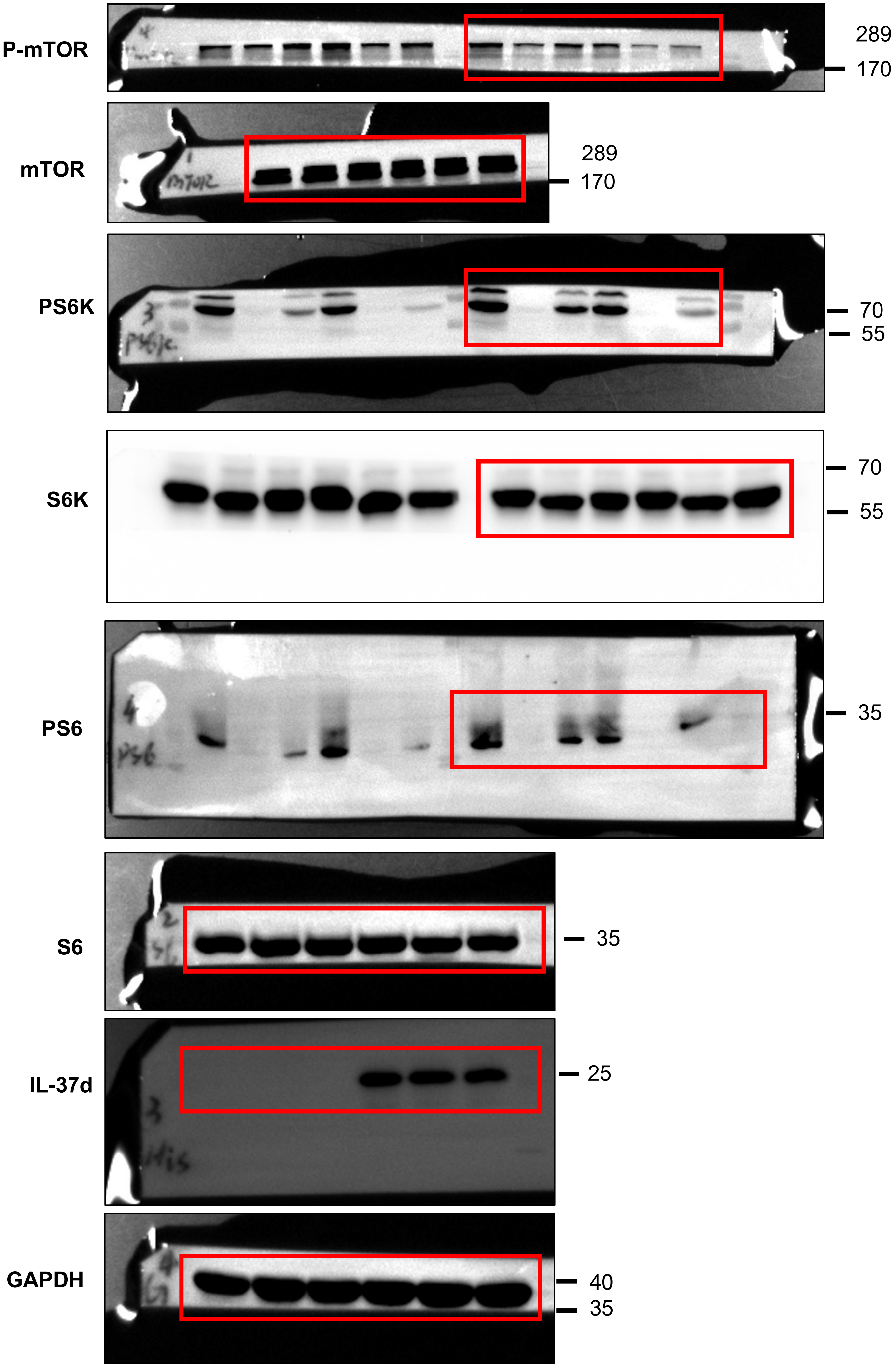

Supplemental Material to Fig S1d (original blots)

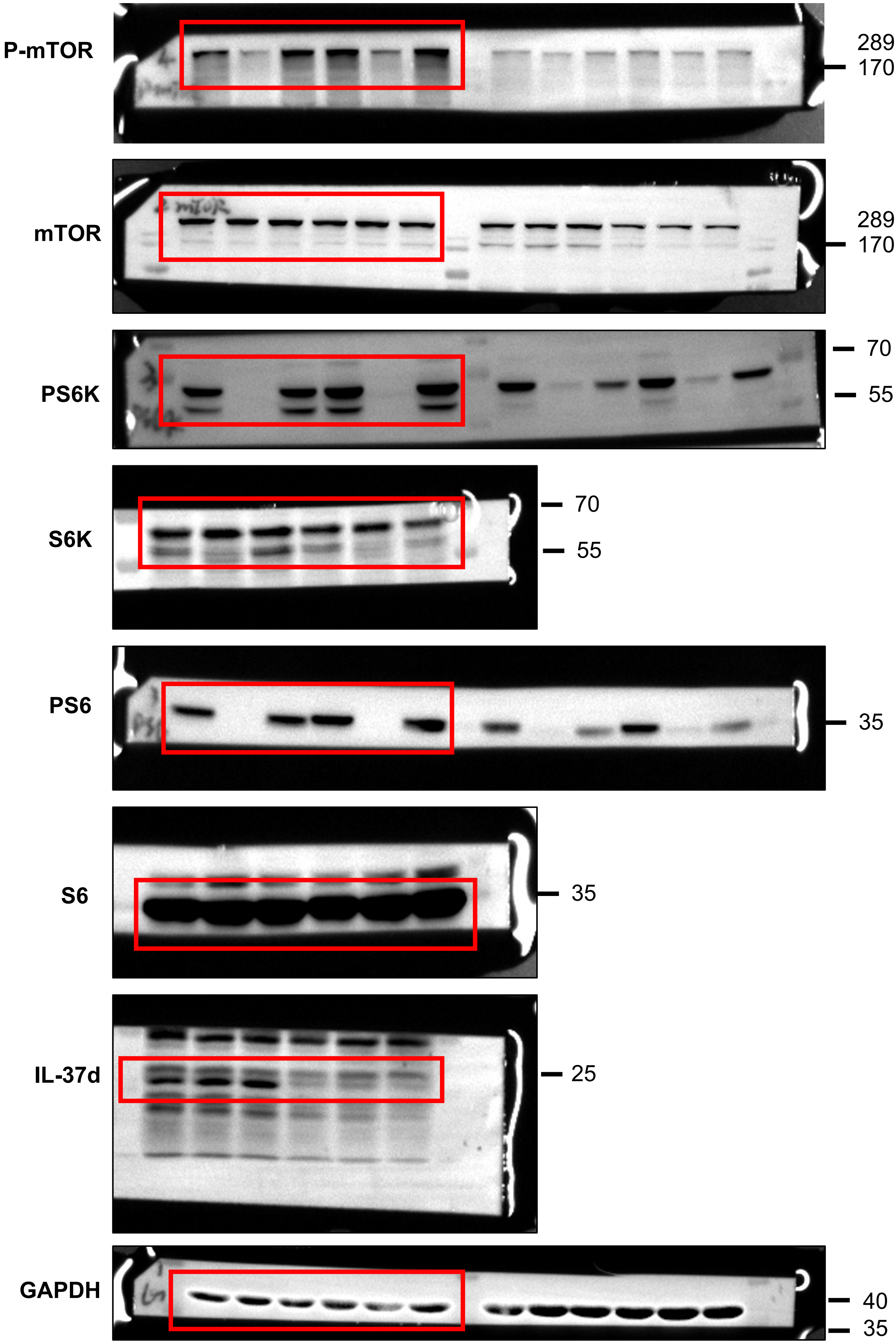

Supplemental Material to Fig S2a (original blots)

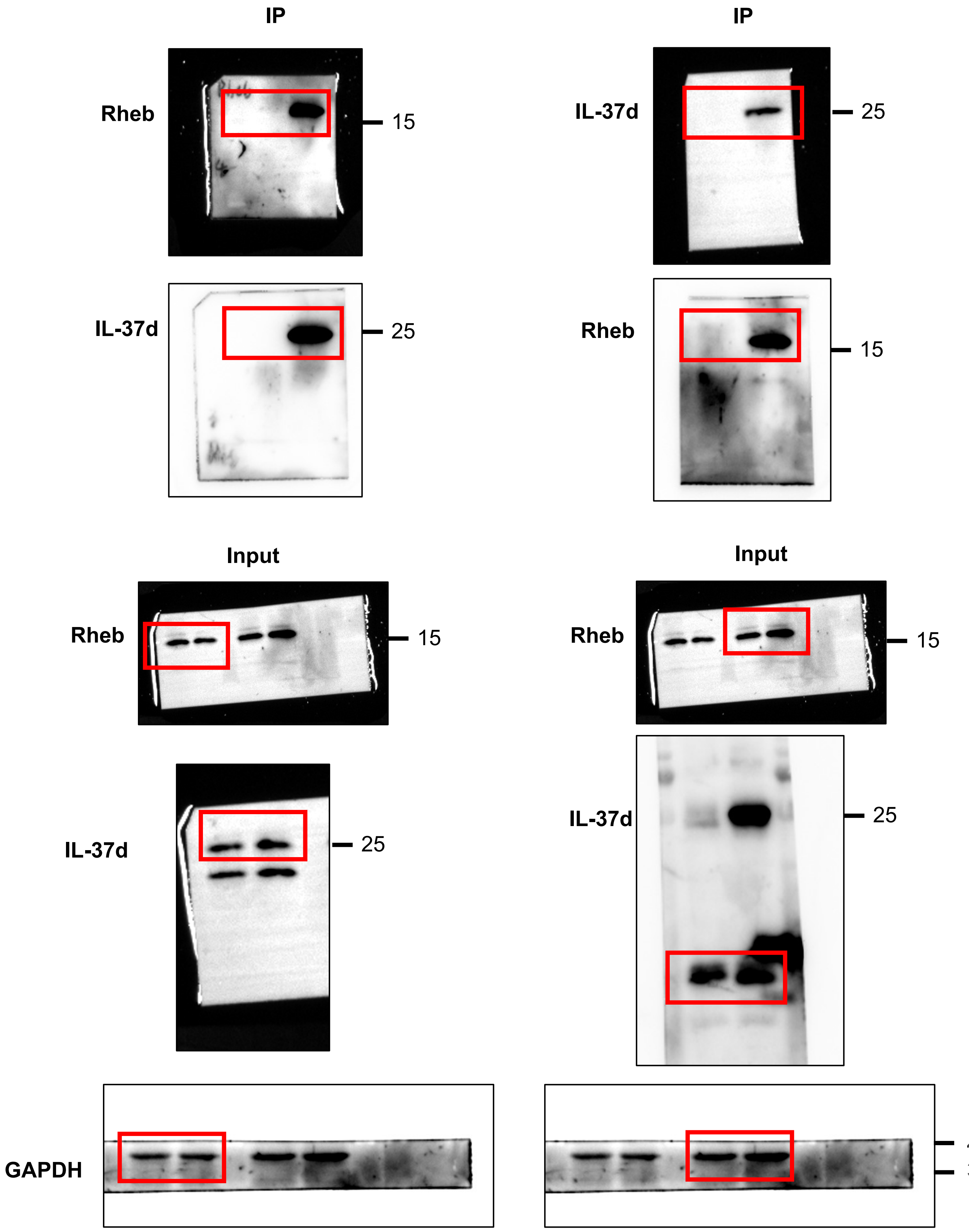

Supplemental Material to Fig S4b (original blots)

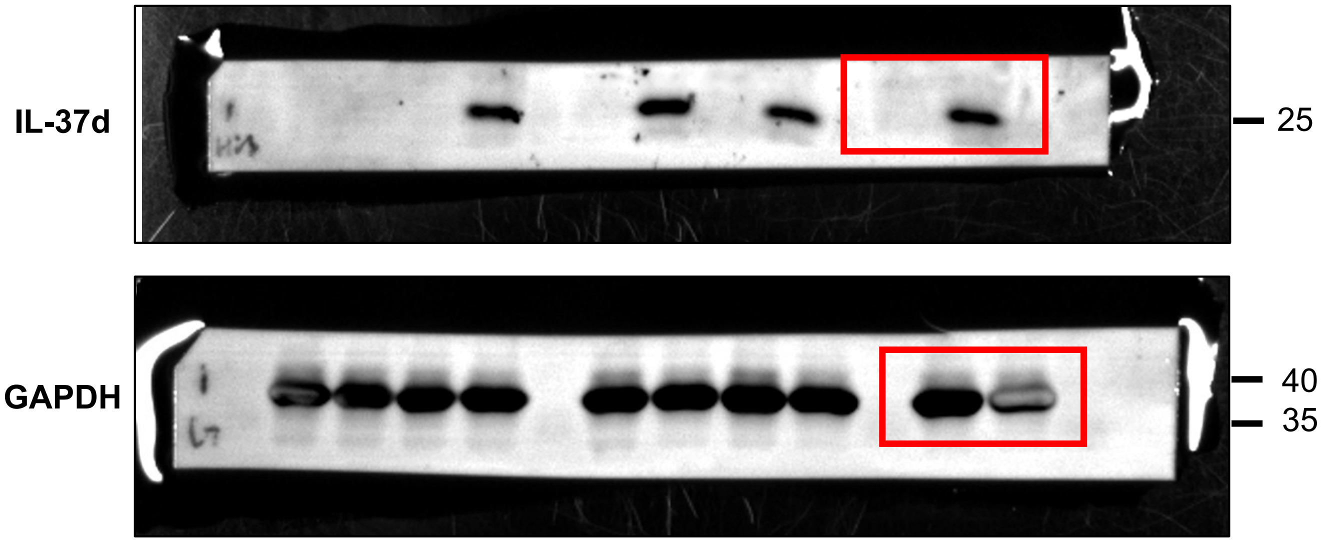

Supplemental Material to Fig S6a-d (original blots)

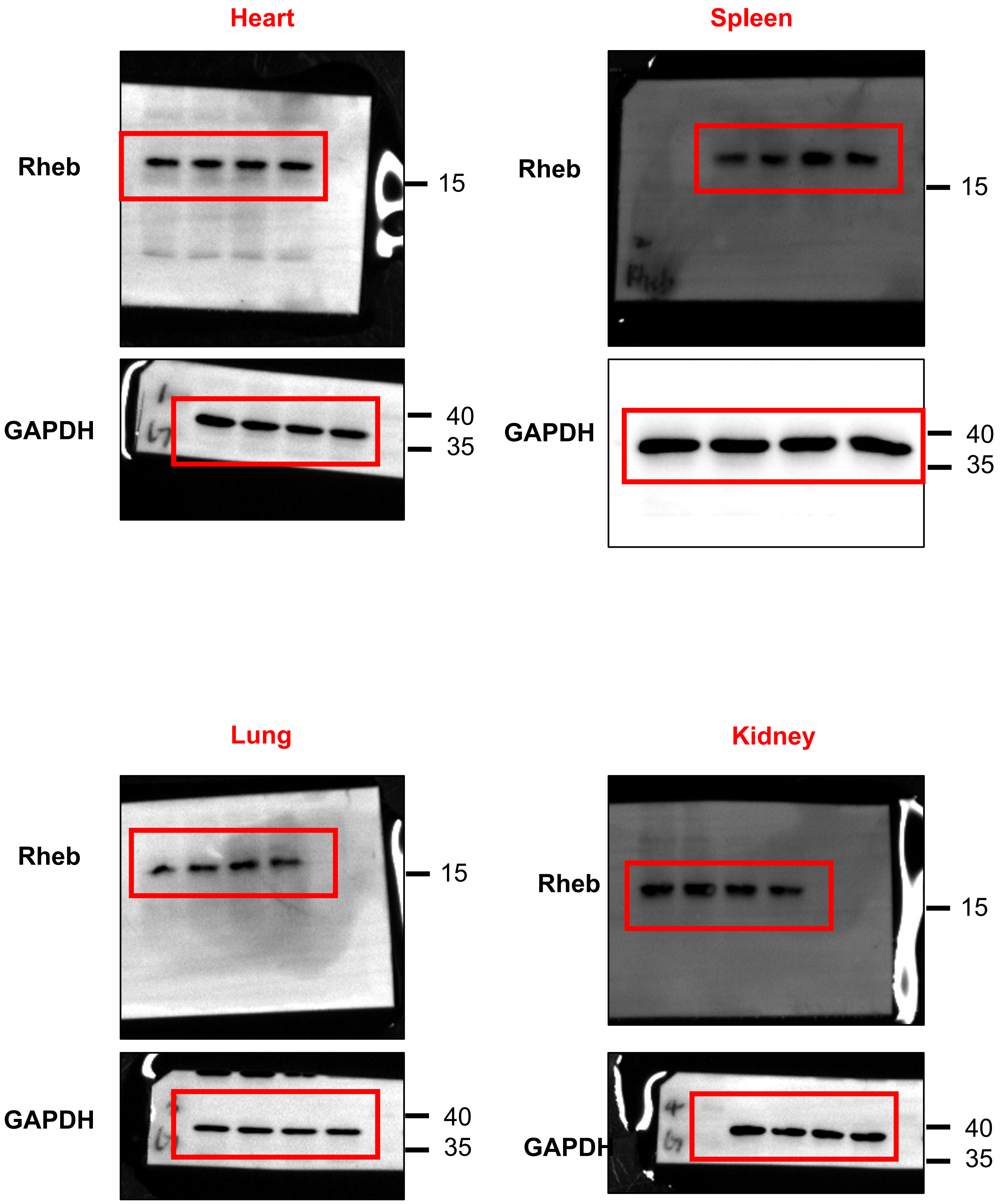

Supplemental Material to Fig S7a-d (original blots)

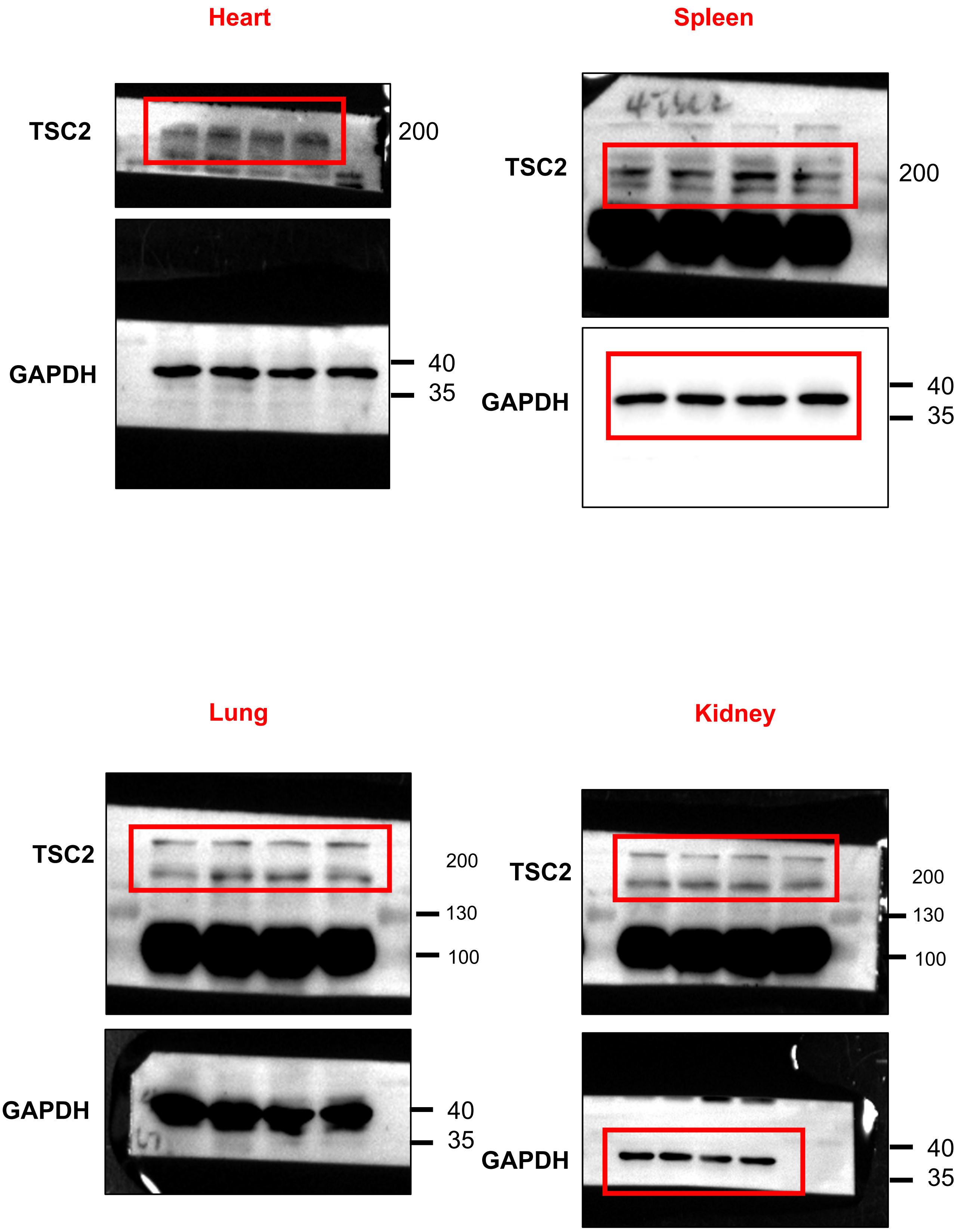

Table S1: List of key resources used in this study

REAGENT or RESOURCE	SOURCE	IDENTIFIER
**Antibodies**		
Anti-IL-37d	Invitrogen	PA5-30527
Anti-Rheb	New East	21098
Anti-Rheb-GTP	New East	26910
Anti-S6	CST	64108SF
Anti-p-S6	CST	81736SF
Anti-S6K	CST	9202S
Anti-p-S6K	CST	9204S
p-p70 S6 kinase α Alexa Fluor® 594	Santa Cruz	sc-8416 AF594
Anti-mTOR	CST	2983S
Anti-P-mTOR	CST	5536S
Anti-TSC2	Santa Cruz	sc-271314
Anti-SREBP-1	BD	557036
Anti-Lipin1	CST	14906
Anti-CPT-1α	Abcam	ab128568
Anti-LAMP1	Abcam	ab25245
Anti-LAMP2	Abcam	ab13524
Anti-GAPDH	ZSGB-Bio	TA-08
Anti-His	ZSGB-Bio	TA-02
Anti-Flag	Sigma-Aldrich	F7425
Alexa Fluor® 488	Abcam	ab150113
Alexa Fluor® 594	Abcam	ab150080
Alexa Fluor® 647	Abcam	ab150167
**Chemical Regents**		
Anti-Flag magnetic beads	Bimake	B26101
Anti-His magnetic beads	Sigma-Aldrich	H9914
Ni-NTA His bind	Sigma-Aldrich	70666-4
IPTG	Sangon Biotech	B300845-0005
Edible alcohol	N/A	N/A

Liquid Ethanol Diet	Nanjing Cellnuo Biotechnology	#10303
Protease inhibitor cocktail	Bimake	B14002
Protein A/G-Sepharose	Santa Cruz	sc-2003
Lipofectamine 2000	Invitrogen	Cat#11668019
Ethanol absolute	HUSHI	10009218
Oil Red O staining solution	Servicebio	G1015
Blue cell viability dye	Sigma-Aldrich	T8154
Cell Counting Kit-8	APEXBIO	K1018
eBioscience™ Fixable Viability Dye eFluor™ 660	Invitrogen	65-0864-14
Phenylmethylsulfonyl fluoride (PMSF)	Sigma-Aldrich	Cat#52332
DAPI	Invitrogen	Cat#S36964
**Critical Commercial Assays**		
Lysosome isolation Kit	Bestbio	Cat #BB31452
Endo-free plasmid Midi-Kit	CWBio	Cat#CW2105S
eECL Western Blot Kit	Millipore	Cat#69078
Alanine aminotransferase Assay Kit	Nanjing Jiancheng Bioengineering Institute	Cat#C009-2-1
Aspartate aminotransferase Assay Kit	Nanjing Jiancheng Bioengineering Institute	Cat#C010-2-1
Triglyceride Assay Kit	Nanjing Jiancheng Bioengineering Institute	Cat#A110-1-1
Total cholesterol assay kit	Nanjing Jiancheng Bioengineering Institute	Cat#A111-1-1
Rheb Pulldown Activation Assay kit	NewEast Biosciences	Cat＃81201
RHEB Human Recombinant Protein	OriGene	Cat＃TP300307
Switch 1(Biotin-DN-9)	GL Biochem (Shanghai)	Customized service
Switch 2(Biotin-GN-12)	GL Biochem (Shanghai)	Customized service
CAAX	GL Biochem (Shanghai)	Customized service
HVR-CAAX(Biotin-MS-11)	GL Biochem (Shanghai)	Customized service

**Experimental Models: Cell Lines**		
Cell line: HEK293T	Shanghai Cell Bank of Chinese Aca- demy of Sciences	GNHu17
Cell line: A549	China Center for Type Culture Collection(CCTCC)	GDC0063
Cell line: Huh7	China Center for Type Culture Collection(CCTCC)	
Cell line: HepG2	Shanghai Cell Bank of Chinese Academy of Sciences	SCSP-510
Cell line: MEF($P53^{-/-}$)		N/A
Cell line: MEF($P53^{-/-}$ $TSC2^{-/-}$)		N/A
**Experimental Models: Organism**		
Mouse: C57BL/6J	Charles River	N/A
Bacteria: *Escherichia coli* DH5α	Zhuangmeng Bio	ZK206
Bacteria: *Escherichia coli* BL21(DE3)	Zhuangmeng Bio	ZK202
**RNA interference and QPCR primers**		
si-IL-37d-1：GGGUGACAGAUAAAUUUGATT	Boshang Bio	N/A
si-IL-37d-2：UCAAAUUUAUCUGUCACCCTT	Boshang Bio	N/A
si-TSC2-1：AAGGATTACCCTTCCAACGAA	Boshang Bio	N/A
si-TSC2-2：CGACGAGTCAAACAAGCCAAT	Boshang Bio	N/A
**Recombinant plasmid**		
pET-22b	HonorGene	HG-VYN0161
PET22b-TAT-His-IL37d	This study	N/A
pCDNA3.1-His-IL-37d	This study	N/A
pBiFC-bFosVC155	addgene	22013
pBiFC-bJunVN173	addgene	22012
pBiFC-VC155	Keyanyun Bio	N/A
pBiFC-VN173	Keyanyun Bio	N/A

pBiFC-VC155-Rheb	Keyanyun Bio	N/A
pCDNA3.1-RHEB-Q64L-Myc	Miaoling Bio	P1798
pCDNA3.1-RHEB-Q60K-Myc	Miaoling Bio	P1797
pBiFC-VN173-IL-37d	Keyanyun Bio	N/A
pBiFC-VN173-IL-37d Δβ4	Keyanyun Bio	N/A
pBiFC-VN173-IL-37d Δβ5	Keyanyun Bio	N/A
pBiFC-VN173-IL-37d Δβ6	Keyanyun Bio	N/A
pBiFC-VN173-IL-37d Δα1+2	Keyanyun Bio	N/A
pBiFC-VN173-IL-37d Δβ10+11	Keyanyun Bio	N/A
pBiFC-VN173-IL-37d-A123P	Keyanyun Bio	N/A
pBiFC-VN173-IL-37d-G153D	Keyanyun Bio	N/A
pBiFC-VN173-IL-37d-C160	Keyanyun Bio	N/A
pBiFC-VN173-IL-37d-N161S	Keyanyun Bio	N/A
Rheb	Weizhen Bio	CH881265
Myc-N153T-RHEB	This study	N/A
TSC2	Weizhen Bio	CH826127
pEnCMV-RHOA(human)-3×HA	Miaoling Bio	P21122
pEnCMV-HRAS(human)-FLAG-SV40-Neo	Miaoling Bio	P28783
pEnCMV-RAB5A(human)-HA-SV40-Neo	Miaoling Bio	P31809
**Software**		
Image J		https://imagej.en.softonic.com
Zeiss Zen	Zeiss	https://www.zeiss.com
ZDOCK-Studio Discovery	Dassault Systems BIOVIA	San Diego
GraphPad Prism	GraphPad	https://www.graphpad.com/

Table S2-The Interface Interactions of GDP-Rheb and IL-37d

ResidueName	A:ASP73	A:SER74	A:ASN76	A:GLN128	A:HIS130	A:PRO131	A:TRP175	A:CYS178	A:THR179
A:ARG15	-	-	N	N	-	-	-	-	-
A:SER16	-	-	-	N	N	-	-	-	-
A:ASP33	-	-	-	-	N	-	-	-	-
A:SER34	-	-	-	-	H	-	N	-	-
A:TYR35	-	-	-	-	Pi	-	N	-	-
A:ASP36	-	-	-	-	-	-	-	-	-
A:PRO37	-	-	-	-	N	N	Pi	-	-
A:THR38	-	-	-	-	-	-	-	H	N
A:ILE39	-	-	-	-	-	-	-	N	N
A:GLU40	-	-	-	-	-	-	-	-	N
A:ASN41	-	-	-	-	-	-	-	-	-
A:ALA62	-	-	-	-	-	-	-	-	-
A:GLY63	-	-	-	-	-	-	-	-	-
A:GLN64	-	-	-	-	-	-	-	-	-
A:ASP65	-	-	N	-	-	-	-	-	-
A:TYR67	N	N	N	-	-	-	-	-	-
A:SER68	N	-	-	-	-	-	-	-	-
A:ILE69	N	-	-	-	-	-	-	-	-
A:PRO71	-	-	-	-	-	-	-	-	-
A:THR73	-	-	-	-	-	-	-	-	-
A:TYR74	-	-	-	-	-	-	-	-	-

ResidueName	A:SER180	A:CYS181	A:ASN182	A:CYS183	A:ASN184	A:GLU185	A:PRO186	A:VAL187	A:GLY188
A:ARG15	-	-	-	-	-	-	N	-	-
A:SER16	-	-	-	-	-	-	-	-	-
A:ASP33	-	-	-	-	-	-	-	-	-
A:SER34	-	-	-	-	-	-	-	-	-
A:TYR35	-	-	-	-	-	-	-	-	-
A:ASP36	-	-	-	-	-	-	-	-	-
A:PRO37	-	-	-	-	-	-	-	-	-
A:THR38	N	N	-	-	-	-	N	N	N
A:ILE39	N	N	-	-	-	-	-	-	-
A:GLU40	H	H	H	-	-	N	-	-	-
A:ASN41	-	N	-	-	-	-	-	-	-
A:ALA62	N	-	N	-	-	N	N	-	-
A:GLY63	-	-	-	-	-	N	H	-	-
A:GLN64	-	-	-	-	-	N	N	-	-
A:ASP65	-	-	-	-	-	-	N	-	-
A:TYR67	-	-	-	-	-	-	-	-	-
A:SER68	-	-	-	-	N	N	N	-	-
A:ILE69	-	-	-	N	H	-	-	-	-
A:PRO71	-	-	N	N	N	N	-	-	-
A:THR73	-	-	H	N	-	-	-	-	-
A:TYR74	N	-	N	-	-	H	-	-	-

Table S2-The Interface Interactions of GDP-Rheb and IL-37d

ResidueName	A:VAL189	A:THR190	A:ASP191	A:LYS192	A:GLU194	A:ASN195	A:HIS198
A:ARG15	-	-	-	-	-	-	-
A:SER16	-	-	-	-	-	-	-
A:ASP33	-	-	-	-	-	-	-
A:SER34	-	-	N	H	-	-	-
A:TYR35	-	-	N	N	N	-	-
A:ASP36	N	N	N	N	-	-	-
A:PRO37	H	N	N	-	-	-	-
A:THR38	N	N	-	-	-	-	-
A:ILE39	-	N	-	-	N	N	N
A:GLU40	-	-	-	-	N	-	-
A:ASN41	-	-	-	-	N	-	-
A:ALA62	-	-	-	-	-	-	-
A:GLY63	-	-	-	-	-	-	-
A:GLN64	-	-	-	-	-	-	-
A:ASP65	-	-	-	-	-	-	-
A:TYR67	-	-	-	-	-	-	-
A:SER68	-	-	-	-	-	-	-
A:ILE69	-	-	-	-	-	-	-
A:PRO71	-	-	-	-	-	-	-
A:THR73	-	-	-	-	-	-	-
A:TYR74	-	-	-	-	-	-	-

Table S2-The AminoAcid composition of GDP-Rheb and IL-37d

ResidueTypes	Percentages All	Percentages at Interface
A	6.13	2.17
C	1.53	6.52
D	4.6	10.87
E	7.06	6.52
F	5.21	0
G	5.52	4.35
H	2.45	4.35
I	7.67	4.35
K	8.9	2.17
L	8.59	0
M	1.53	0
N	4.29	10.87
P	3.99	8.7
Q	4.6	4.35
R	3.07	2.17
S	9.82	10.87
T	3.37	8.7
V	7.36	4.35
W	0.92	2.17
Y	3.37	6.52
X	0	0

Table S2-The PI Interactions of GDP-Rheb and IL-37d

ReceptorResidue	LigandResidue	Interaction Constituents	Distance	Type
A:TYR35	A:HIS130	A:TYR35:HN - A:HIS130	2.9582	Pi-Donor
A:PRO37	A:TRP175	A:TRP175 - A:PRO37	5.3495	Pi-Alkyl

Table S2-The Hydrogen Bonds of GDP-Rheb and IL-37d

ReceptorResidue	LigandResidue	Interaction Constituents	Distance	Type
A:GLU40	A:CYS181	A:CYS181:SG - A:GLU40:O	2.9678	Conventional
A:GLU40	A:ASN182	A:ASN182:HD21 - A:GLU40:OE1	2.253	Conventional
A:ILE69	A:ASN184	A:ASN184:HD21 - A:ILE69:O	2.1117	Conventional
A:THR38	A:CYS178	A:THR38:HG1 - A:CYS178:SG	2.3014	Conventional
A:GLU40	A:SER180	A:GLU40:HN - A:SER180:OG	2.6431	Conventional
A:THR73	A:ASN182	A:THR73:HG1 - A:ASN182:OD1	2.116	Conventional
A:TYR74	A:GLU185	A:TYR74:HH - A:GLU185:OE2	2.3509	Conventional
A:GLY63	A:PRO186	A:PRO186:CD - A:GLY63:O	3.1582	Carbon
A:SER34	A:LYS192	A:LYS192:CE - A:SER34:O	3.161	Carbon
A:SER34	A:HIS130	A:SER34:CA - A:HIS130:NE2	3.1057	Carbon
A:PRO37	A:VAL189	A:PRO37:CD - A:VAL189:O	3.0611	Carbon

Table S2-The Contact Surface Area of GDP-Rheb and IL-37d

Residue	Contact Surface Area	Polar Contact Surface Area	Nonpolar Contact Surface Area
A:ASP73	26.1082	23.061399	3.04682
A:SER74	21.469601	10.3903	11.0794
A:ASN76	20.4515	14.0809	6.37063
A:GLN128	12.237	12.237	0
A:HIS130	34.4533	22.266001	12.1873
A:PRO131	3.80133	3.80133	0
A:TRP175	21.3277	0	21.3277
A:CYS178	16.9855	2.78764	14.1979
A:THR179	6.73034	5.06844	1.6619
A:SER180	26.7679	15.9656	10.8024
A:CYS181	26.6586	4.50622	22.152399
A:ASN182	48.653999	39.2365	9.41745
A:CYS183	15.0236	6.84239	8.18123
A:ASN184	31.464001	20.107599	11.3563
A:GLU185	44.3368	38.5201	5.81666
A:PRO186	14.0497	3.80133	10.2484
A:VAL187	0.276985	0	0.276984
A:GLY188	10.034	4.77129	5.26269
A:VAL189	15.7471	15.7471	0
A:THR190	12.5826	10.6437	1.93889
A:ASP191	15.7122	15.7122	0
A:LYS192	33.756699	6.61227	27.1444
A:GLU194	26.400801	21.969	4.43174
A:ASN195	1.32536	1.32536	0
A:HIS198	1.59044	1.59043	0
A:ARG15	3.71101	3.71101	0
A:SER16	12.8947	4.30817	8.5865
A:ASP33	8.86976	8.86976	0
A:SER34	38.183998	33.198299	4.98571
A:TYR35	24.312901	16.003401	8.30951
A:ASP36	30.013599	15.3335	14.6801
A:PRO37	30.1912	0	30.1912
A:THR38	52.096901	30.492201	21.6047
A:ILE39	21.881701	0	21.881701
A:GLU40	48.033798	32.245701	15.7881
A:ASN41	15.0628	11.739	3.32381
A:ALA62	20.1483	1.59043	18.557899
A:GLY63	18.7882	18.7882	0
A:GLN64	0.276986	0	0.276983
A:ASP65	13.4785	12.9245	0.553967
A:TYR67	46.097599	10.6437	35.453899
A:SER68	5.14488	1.26711	3.87777
A:ILE69	29.318001	17.130699	12.1873
A:PRO71	40.439602	0	40.439602
A:THR73	12.7889	11.404	1.38492
A:TYR74	20.2439	11.6574	8.5865

Table S3-The Interface Interactions of GTP-Rheb and IL-37d

ResidueName	A:SER61	A:VAL71	A:LEU72	A:ASP73	A:SER74	A:VAL80	A:ASN84	A:TYR85	A:ILE86
A:ILE11	-	-	-	-	-	-	-	-	-
A:TYR14	N	-	-	-	-	-	-	-	-
A:ARG15	N	-	-	-	-	-	-	-	N
A:SER16	-	-	-	-	-	-	-	-	-
A:LYS19	-	-	-	-	-	-	-	-	-
A:SER20	-	-	-	-	-	-	-	-	-
A:ASP33	-	-	-	-	-	-	-	-	-
A:TYR35	-	-	N	-	-	-	-	-	-
A:ASP36	-	-	N	H	H	-	-	-	-
A:PRO37	-	N	N	-	-	-	-	-	-
A:THR38	-	-	-	-	-	-	-	N	N
A:ILE39	-	-	-	-	-	N	-	N	N
A:ASP60	-	-	-	-	-	-	-	-	-
A:THR61	-	-	-	-	-	-	-	N	-
A:ALA62	-	-	-	-	-	-	N	N	N
A:GLY63	-	-	-	-	-	-	N	N	N
A:GLN64	-	-	-	-	-	-	N	N	N
A:TYR67	-	-	-	-	-	-	-	-	-
A:SER68	-	-	-	-	-	-	H	-	-
A:ILE69	-	-	-	-	-	-	H	-	-
A:PRO71	-	-	-	-	-	-	N	N	-
A:TYR74	-	-	-	-	-	-	-	N	-
A:SER89	-	-	-	-	-	-	-	-	-
A:LYS91	-	-	-	-	-	-	-	-	-
A:LYS120	-	-	-	-	-	-	-	-	-

ResidueName	A:ARG87	A:PRO88	A:GLU89	A:ILE90	A:SER114	A:LYS115	A:GLU117	A:GLN204	A:PRO205
A:ILE11	N	-	-	-	-	-	-	-	-
A:TYR14	-	-	-	N	-	-	-	-	H
A:ARG15	N	H	-	N	-	-	-	N	N
A:SER16	N	N	-	N	-	N	-	-	-
A:LYS19	N	-	-	-	-	-	-	-	-
A:SER20	H	-	-	-	-	-	-	-	-
A:ASP33	-	-	-	-	-	-	N	-	-
A:TYR35	N	H	N	N	H	N	-	-	-
A:ASP36	N	-	-	-	N	-	-	-	-
A:PRO37	N	N	N	N	-	-	-	-	-
A:THR38	H	-	-	-	-	-	-	-	-
A:ILE39	N	-	-	-	-	-	-	-	-
A:ASP60	H	-	-	-	-	-	-	-	-
A:THR61	N	-	-	-	-	-	-	-	-
A:ALA62	N	-	-	-	-	-	-	-	-
A:GLY63	N	N	-	-	-	-	-	-	-
A:GLN64	-	-	-	-	-	-	-	-	-
A:TYR67	-	-	-	-	-	-	-	N	-
A:SER68	-	-	-	-	-	-	-	-	-
A:ILE69	-	-	-	-	-	-	-	-	-
A:PRO71	-	-	-	-	-	-	-	-	-
A:TYR74	-	-	-	-	-	-	-	-	-
A:SER89	-	-	-	-	-	-	-	-	-
A:LYS91	-	-	-	-	-	-	-	-	-
A:LYS120	-	-	-	-	-	N	-	-	-

Table S3-The Interface Interactions of GTP-Rheb and IL-37d

ResidueName	A:VAL206
A:ILE11	-
A:TYR14	N
A:ARG15	N
A:SER16	-
A:LYS19	-
A:SER20	-
A:ASP33	-
A:TYR35	-
A:ASP36	-
A:PRO37	-
A:THR38	-
A:ILE39	-
A:ASP60	-
A:THR61	-
A:ALA62	-
A:GLY63	-
A:GLN64	-
A:TYR67	-
A:SER68	-
A:ILE69	-
A:PRO71	-
A:TYR74	-
A:SER89	N
A:LYS91	N
A:LYS120	-

Table S3-The AminoAcid composition of GTP-Rheb and IL-37d

ResidueTypes	Percentages All	Percentages at Interface
A	6.1	2.27
C	1.52	0
D	4.57	9.09
E	7.01	4.55
F	5.18	0
G	5.49	2.27
H	2.74	0
I	7.62	11.36
K	8.84	9.09
L	8.54	2.27
M	1.52	0
N	4.27	2.27
P	4.27	9.09
Q	4.57	4.55
R	3.05	4.55
S	9.76	15.91
T	3.35	4.55
V	7.32	6.82
W	0.91	0
Y	3.35	11.36
X	0	0

Table S3-The Salt Bridges of GTP-Rheb and IL-37d

ReceptorResidue	LigandResidue	Interaction Constituents	Distance
A:ASP60	A:ARG87	A:ARG87:HH12 - A:ASP60:OD2	1.93

Table S3-The Hydrogen Bonds of GTP-Rheb and IL-37d

ReceptorResidue	LigandResidue	Interaction Constituents	Distance	Type
A:ASP36	A:SER74	A:SER74:HN - A:ASP36:OD2	2.2496	Conventional
A:THR38	A:ARG87	A:ARG87:HN - A:THR38:O	1.9495	Conventional
A:SER20	A:ARG87	A:ARG87:HH11 - A:SER20:OG	2.2256	Conventional
A:THR38	A:ARG87	A:ARG87:HH11 - A:THR38:OG1	2.0801	Conventional
A:ASP60	A:ARG87	A:ARG87:HH22 - A:ASP60:OD1	1.95	Conventional
A:TYR35	A:SER114	A:SER114:HG - A:TYR35:O	2.9866	Conventional
A:TYR14	A:PRO205	A:TYR14:HH - A:PRO205:O	2.6119	Conventional
A:TYR35	A:PRO88	A:TYR35:HH - A:PRO88:O	2.0706	Conventional
A:ILE69	A:ASN84	A:ILE69:HN - A:ASN84:OD1	2.4126	Conventional
A:ASP36	A:ASP73	A:ASP73:CA - A:ASP36:OD2	3.2253	Carbon
A:ARG15	A:PRO88	A:ARG15:CD - A:PRO88:O	3.7756	Carbon
A:SER68	A:ASN84	A:SER68:CB - A:ASN84:OD1	3.122	Carbon

Table S3-The Contact Surface Area of GTP-Rheb and IL-37d

Residue	Contact Surface Area	Polar Contact Surface Area	Nonpolar Contact Surface Area
A:SER61	9.79496	7.85608	1.93889
A:VAL71	6.92459	0	6.92459
A:LEU72	37.626598	24.3314	13.2952
A:ASP73	6.5709	4.07805	2.49285
A:SER74	5.83158	5.83158	0
A:VAL80	4.98571	0	4.98571
A:ASN84	80.941704	64.0457	16.896
A:TYR85	47.0056	18.753201	28.2523
A:ILE86	32.519402	13.9615	18.557899
A:ARG87	140.535995	101.759003	38.777699
A:PRO88	38.801899	17.7512	21.0508
A:GLU89	33.0434	24.179899	8.86348
A:ILE90	22.7871	4.50622	18.280899
A:SER114	13.3251	8.61634	4.70873
A:LYS115	11.133	11.133	0
A:GLU117	0.506836	0.506843	0
A:GLN204	1.52054	1.52053	0
A:PRO205	7.60265	7.60265	0
A:VAL206	35.2953	10.6437	24.6516
A:ILE11	2.2808	2.2808	0
A:TYR14	26.4203	16.7258	9.69443
A:ARG15	47.879601	19.350201	28.529301
A:SER16	10.0306	5.32186	4.70872
A:LYS19	18.0784	4.50622	13.5722
A:SER20	8.83796	8.56097	0.276983
A:ASP33	0.253418	0.253422	0
A:TYR35	62.916	34.3867	28.529301
A:ASP36	26.385201	23.061399	3.32381
A:PRO37	56.970001	4.34312	52.6269
A:THR38	33.041302	26.3937	6.64761
A:ILE39	39.108601	20.273701	18.8349
A:ASP60	10.7851	9.12318	1.6619
A:THR61	13.4314	13.4314	0
A:ALA62	36.505699	13.2391	23.2666
A:GLY63	29.985701	26.384899	3.60079
A:GLN64	11.538	2.12058	9.41745
A:TYR67	1.10792	0	1.10793
A:SER68	7.20158	0	7.20158
A:ILE69	24.8568	14.3314	10.5254
A:PRO71	10.4658	1.32536	9.14046
A:TYR74	9.33524	6.84239	2.49285
A:SER89	0.253425	0.253421	0
A:LYS91	13.8492	0	13.8492
A:LYS120	6.09665	6.09666	0

Table S4: List of oligo primers used in this study

QPCR primer	Forward (5'to3')	Reverse (5'to3')
**m-SREBP-1c**	GGAGCCATGGATTGCACATT	GGCCCGGGAAGTCACTGT
**m-ACC1**	TGACAGACTGATCGCAGAGAAAG	TGGAGAGCCCCACACACA
**m-FAS**	GCTGCGGAAACTTCAGGAAAT	AGAGACGTGTCACTCCTGGACTT
**m-SCD1**	TTCTTCTCTCACGTGGGTTG	CGGGCTTGTAGTACCTCCTC
**m-PPARα**	GGGCAGAGCAAGTCATCTTC	CCTCTGGAAGCACTGAGGAC
**m-CPT-1α**	CCAGGCTACAGTGGGACATT	GAACTTGCCCATGTCCTTGT
**m-PGC-1α**	AAGAGCGCCGTGTGATTTAC	ACGGTGCATTCCTCAATTTC
**m-GAPDH**	TGCGACTTCAACAGCAACTC	CTTGCTCAGTGTCCTTGCTG
**h-IL-37d**	TGAACCCCAGTGCTGCTTAG	CCCAGAGTCCAGGACCAGTA
**h-β-actin**	AGCCTCGCCTTTGCCGA	CTGGTGCCTGGGGCG
