# Supplementary material for: IL-37d suppresses Rheb-mTORC1 axis independently of TCS2 to alleviate alcoholic liver disease
Source: Commun Biol. 2024 Jun 21;7:756. doi: 10.1038/s42003-024-06427-8 (PMC11192940; doi:10.1038/s42003-024-06427-8)
Supplement: Supplementary file 4 — Reporting Summary [file 42003_2024_6427_MOESM4_ESM.pdf]

nature portfolio

Corresponding author(s): DBPR, COMMSBIO-23-4646

Last updated by author(s): May 16, 2024

Reporting Summary

Nature Portfolio wishes to improve the reproducibility of the work that we publish. This form provides structure for consistency and transparency in reporting. For further information on Nature Portfolio policies, see our Editorial Policies and the Editorial Policy Checklist.

Statistics

For all statistical analyses, confirm that the following items are present in the figure legend, table legend, main text, or Methods section.

n/a	Confirmed	
☐	☒	The exact sample size (n) for each experimental group/condition, given as a discrete number and unit of measurement
☐	☒	A statement on whether measurements were taken from distinct samples or whether the same sample was measured repeatedly
☒	☐	The statistical test(s) used AND whether they are one- or two-sided<br>*Only common tests should be described solely by name; describe more complex techniques in the Methods section.*
☐	☒	A description of all covariates tested
☒	☐	A description of any assumptions or corrections, such as tests of normality and adjustment for multiple comparisons
☐	☒	A full description of the statistical parameters including central tendency (e.g. means) or other basic estimates (e.g. regression coefficient) AND variation (e.g. standard deviation) or associated estimates of uncertainty (e.g. confidence intervals)
☒	☐	For null hypothesis testing, the test statistic (e.g. F, t, r) with confidence intervals, effect sizes, degrees of freedom and P value noted<br>*Give P values as exact values whenever suitable.*
☒	☐	For Bayesian analysis, information on the choice of priors and Markov chain Monte Carlo settings
☒	☐	For hierarchical and complex designs, identification of the appropriate level for tests and full reporting of outcomes
☒	☐	Estimates of effect sizes (e.g. Cohen's d, Pearson's r), indicating how they were calculated

Our web collection on statistics for biologists contains articles on many of the points above.

Software and code

Policy information about availability of computer code

Data collection	N/A
Data analysis	N/A

For manuscripts utilizing custom algorithms or software that are central to the research but not yet described in published literature, software must be made available to editors and reviewers. We strongly encourage code deposition in a community repository (e.g. GitHub). See the Nature Portfolio guidelines for submitting code & software for further information.

Data

Policy information about availability of data

All manuscripts must include a data availability statement. This statement should provide the following information, where applicable:

- Accession codes, unique identifiers, or web links for publicly available datasets
- A description of any restrictions on data availability
- For clinical datasets or third party data, please ensure that the statement adheres to our policy

Data from this study are available on request.

Research involving human participants, their data, or biological material

Policy information about studies with human participants or human data. See also policy information about sex, gender (identity/presentation), and sexual orientation and race, ethnicity and racism.

Reporting on sex and gender	N/A
Reporting on race, ethnicity, or other socially relevant groupings	N/A
Population characteristics	N/A
Recruitment	N/A
Ethics oversight	N/A

Note that full information on the approval of the study protocol must also be provided in the manuscript.

Field-specific reporting

Please select the one below that is the best fit for your research. If you are not sure, read the appropriate sections before making your selection.

☒ Life sciences ☐ Behavioural & social sciences ☐ Ecological, evolutionary & environmental sciences

For a reference copy of the document with all sections, see nature.com/documents/nr-reporting-summary-flat.pdf

Life sciences study design

All studies must disclose on these points even when the disclosure is negative.

Sample size	In the Material and Methods section on line 146-166, and Figure legends section.
Data exclusions	N/A
Replication	Each experiment was repeated 3 times and the sample size of each group in animal experiments was n≥3
Randomization	In the Material and Methods section on line 154-166, and Figure legends section
Blinding	In the Material and Methods section on line 154-166

Reporting for specific materials, systems and methods

We require information from authors about some types of materials, experimental systems and methods used in many studies. Here, indicate whether each material, system or method listed is relevant to your study. If you are not sure if a list item applies to your research, read the appropriate section before selecting a response.

Materials & experimental systems

n/a	Involved in the study	
☐	☒	Antibodies
☐	☒	Eukaryotic cell lines
☒	☐	Palaeontology and archaeology
☐	☒	Animals and other organisms
☒	☐	Clinical data
☒	☐	Dual use research of concern
☒	☐	Plants

Methods

n/a	Involved in the study	
☒	☐	ChIP-seq
☐	☒	Flow cytometry
☒	☐	MRI-based neuroimaging

Antibodies

Antibodies used	A list of the antibodies used in the article is listed in the "KEY RESOURCES TABLE" section of the article.
Validation	A list of the antibodies used in the article is listed in the "KEY RESOURCES TABLE" section of the article.

Eukaryotic cell lines

Policy information about cell lines and Sex and Gender in Research

Cell line source(s)	In the Material and Methods section on line 203-214.
Authentication	All cell lines used in this research were purchased from Chinese Academy of Sciences (Shanghai,China) or China Center for Type Culture Collection (Wuhan, China) and are commercialized and proven.
Mycoplasma contamination	All cell lines tested negative for mycoplasma.
Commonly misidentified lines (See ICLAC register)	N/A

Animals and other research organisms

Policy information about studies involving animals; ARRIVE guidelines recommended for reporting animal research, and Sex and Gender in Research

Laboratory animals	Eight- to ten-week-old male mice with body weight more than 20 g were used in our experiments.
Wild animals	In the Material and Methods section on line 146-166.
Reporting on sex	Male mice were used in our study .
Field-collected samples	The mice were housed in specific pathogen-free conditions on a 12 h light/dark cycle at 18-22°C and allowed free access to sterilized food and water.
Ethics oversight	All animal experiments have been approved by the Ethics Committee of Basic Science of Medical School, Shandong University.

Note that full information on the approval of the study protocol must also be provided in the manuscript.

Plants

Seed stocks	N/A
Novel plant genotypes	N/A
Authentication	N/A

Flow Cytometry

Plots

Confirm that:

- [x] The axis labels state the marker and fluorochrome used (e.g. CD4-FITC).
- [x] The axis scales are clearly visible. Include numbers along axes only for bottom left plot of group (a 'group' is an analysis of identical markers).
- [x] All plots are contour plots with outliers or pseudocolor plots.
- [x] A numerical value for number of cells or percentage (with statistics) is provided.

Methodology

Sample preparation	In the Material and Methods section and Figure legends section.
Instrument	In the Material and Methods section and Figure legends section.
Software	In the Material and Methods section and Figure legends section.
Cell population abundance	In the Material and Methods section and Figure legends section.

Gating strategy

In the Material and Methods section and Figure legends section.

☒ Tick this box to confirm that a figure exemplifying the gating strategy is provided in the Supplementary Information.
